# Freezing-Activated Covalent Organic Frameworks for Precise Fluorescence Cryo-Imaging of Cancer Tissue

**DOI:** 10.1021/jacs.4c13848

**Published:** 2025-02-27

**Authors:** Farah Benyettou, Gobinda Das, Maylis Boitet, Sabu Varghese, Mostafa Khair, Akshaya Kumar Das, Zineb Matouk, Thirumurugan Prakasam, Philippe Bazin, Sudhir Kumar Sharma, Sneha Thomas, Yao He, Rainer Straubinger, Bikash Garai, Ramesh Jagannathan, Felipe Gándara, Mohamad El-Roz, Ali Trabolsi

**Affiliations:** † Chemistry Program, New York University Abu Dhabi, Abu Dhabi 129188, United Arab Emirates; ‡ Core Technology Platforms, New York University Abu Dhabi, 129188 Abu Dhabi, United Arab Emirates; § Technology Innovative Institute, Abu Dhabi 9639, United Arab Emirates; ∥ Normandie Univ, ENSICAEN, UNICAEN, CNRS, LCS, Caen 14000, France; ⊥ Engineering Program, New York University Abu Dhabi, Abu Dhabi 129188, United Arab Emirates; # Instituto de Ciencia de Materiales de Madrid-CSIC, C. Sor Juana Inés de la Cruz 3, 28049 Madrid, Spain

## Abstract

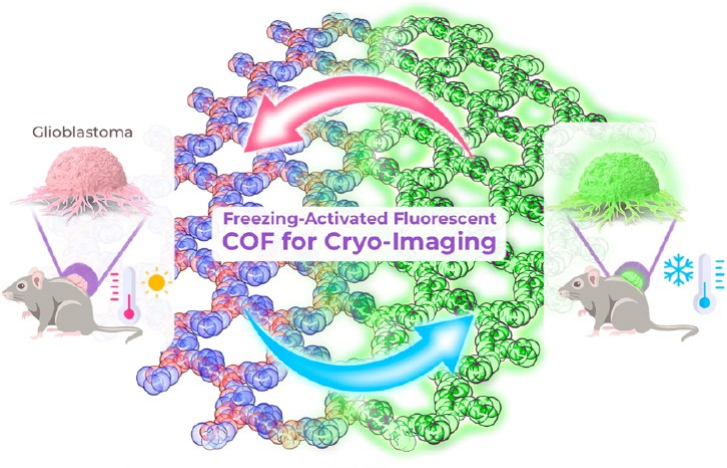

Cryosurgery represents a transformative approach in the treatment of resistant tumors, utilizing extreme cold to selectively ablate malignant tissue. However, the clinical success of this technique is constrained by the limited ability of current imaging techniques to differentiate effectively between cancerous and healthy tissues with high spatial resolution. To overcome this challenge, we present a nanoscale Covalent Organic Framework, nTG-DFP-COF, specifically designed to enhance fluorescence-guided cryo-imaging. This framework exhibits a unique temperature-dependent luminescence, that results in enhanced fluorescence emission under cryogenic conditions, enabling precise tissue differentiation during surgical procedures. Engineered for biocompatibility and water dispersibility, nTG-DFP-COF demonstrates minimal cytotoxicity and exceptional specificity toward cancer cells. Comprehensive in vitro, in vivo, and ex vivo evaluations confirm its structural stability and functional efficacy under cryogenic conditions. This innovation not only enhances the precision and safety of cryosurgical procedures but also advances the integration of diagnostic and therapeutic functionalities into a unified platform. By substantially improving tumor targeting accuracy, the use of nTG-DFP-COF will reduce the need for repeat surgeries, facilitate faster recovery, and minimize healthcare costs, thus setting a new standard in oncologic imaging and intervention.

## Introduction

Cryosurgery is a pivotal advancement in the minimally invasive treatment of cancers resistant to conventional therapies. It uses extreme cold, typically between −20 and −40 °C, to effectively eradicate malignant tissue.^1^ This technique offers numerous benefits, including reduced pain, minimal bleeding, and lower morbidity rates, and is particularly beneficial for tumors that do not respond to radiotherapy.^2−5^ The less invasive nature of cryosurgery enhances patient comfort and promotes faster recovery and fewer surgical complications. However, the efficacy and safety of cryosurgery depend critically on accurate real-time visualization of the target tissue.^2,6^ Accurate imaging is essential to minimize damage to surrounding healthy tissue and ensure comprehensive removal of cancer cells to optimize the therapeutic outcomes of cryosurgery. Current imaging techniques such as ultrasound, computed tomography (CT), and magnetic resonance imaging (MRI) are essential for modern cryosurgery, but due to their limited resolution at the molecular level, they are often unable to determine the extent of frozen tissue reliably.^1^

Noninvasive optical fluorescence imaging has emerged as a promising approach to improve tumor visualization during surgical interventions.^7^ This method leverages fluorescent dyes to provide direct, real-time insights at the molecular level, which is very helpful in accurately differentiating cancerous from healthy tissue during diagnosis and therapy. Despite its potential, the application of fluorescence imaging in real-time guided cryosurgery remains largely unexplored. This is mainly due to the challenges of tumor-specific visualization under freezing conditions and the necessary sensitivity to intracellular ice formation, which is crucial for effectively destroying cancer cells. Traditional organic luminescent materials often suffer from reduced stability and sensor sensitivity at low temperatures, which can affect performance and reliability due to issues such as aggregation-induced quenching and low photostability. In response to these challenges, He et al. have developed a novel strategy that utilizes the aggregation-induced emission (AIE) of fluorogens.^8^ This innovative approach significantly enhances real-time monitoring capabilities in cryosurgery by activating fluorescent probes in cold environments to improve surgical precision and effectively differentiate between malignant and benign tissue. This development underscores the urgent need for advanced imaging technologies that can adapt to the unique requirements of cryogenic conditions, redefining the effectiveness and safety of cryosurgical cancer treatment.

Recent advances in materials science have highlighted the potential of Covalent Organic Frameworks (COFs)^9^ in biomedical fields.^10−16^ These frameworks are characterized by their highly tunable structure and porosity, which can be precisely tailored to meet specific clinical requirements.^10−16^ The inherent crystallinity of COFs provides consistent and predictable behavior, essential for diverse medical applications. Moreover, their adjustable pore sizes enable the encapsulation of various therapeutic agents, ranging from small-molecule drugs to larger biologicals, ensuring controlled release that can be fine-tuned by modifying the framework. These properties, combined with exceptional chemical stability and biocompatibility, make COFs ideal candidates for targeted drug delivery, diagnostic applications, and scaffolds for tissue engineering.^13−22^

Compared to conventional fluorescent organic solids, fluorescent COFs possess rigid chemical structures that inherently minimize the intramolecular rotation, vibration, and motion typically observed in standard organic molecules and polymers.^23^ This stabilization effectively restricts bond motion and blocks nonradiative pathways, which enhances fluorescence by conserving excited-state energy and reducing energy decay.^9^ We have pioneered the field of bioimaging using COFs through the development of fluorescent covalent organic nanosheets capable of selective intracellular localization in the cellular nucleus, providing a unique approach for cancer diagnosis and therapy without the need for external targeting agents.^17^ Consequently, through careful design and synthesis, fluorescent COFs can outperform conventional dissolved organic dyes and compete with conventional organic solid-based luminescent materials, providing a robust platform for biomedical imaging.^23^

Previously, we synthesized a responsive COF with guanidinium and diformylpyridine linkers (TG-DFP-COF) that exhibits high emission sensitivity to changes in temperature and humidity.^24^ At low temperatures, the reduced molecular motion within the TG-DFP networks stabilizes the hydrogen bonds, which increases the emission intensity by reducing the nonradiative relaxation pathways. Conversely, these hydrogen bonds are weakened at high temperatures, leading to a significant decrease in emission intensity and a substantial bathochromic shift. Developing materials that perform well over a wide temperature range, especially under extreme conditions, is challenging as organic luminescent materials often exhibit reduced stability or low sensor sensitivity at low temperatures, which can affect performance and reliability.

The field of image-guided cryosurgery is currently facing major challenges, including the lack of fluorescent probes capable of specifically and sensitively distinguishing tumor tissue from normal tissue in a frozen state—an essential factor in achieving precise surgical margins. Moreover, most existing probes do not work effectively when intracellular ice forms, a crucial process to induce cancer cell death during cryoablation (Table S1 for the limited literature). Additionally, there is an urgent need to develop nontoxic, nanoscale materials that can circulate in the bloodstream and target tumors without causing adverse effects. The integration of nanoscale COFs with temperature-dependent fluorescence properties could revolutionize image-guided cryosurgery and improve both the safety and efficacy of the procedure.

We introduce nTG-DFP-COF, a nanoscale Covalent Organic Framework (COF), as a robust and biocompatible fluorescent probe designed specifically for real-time cryo-imaging. Its ability to maintain enhanced fluorescence at low temperatures, combined with superior biocompatibility and high specificity for cancer tissue, makes it an ideal tool for the precise detection of tumors while sparing neighbouring healthy tissue. Additionally, nTG-DFP-COF retains its fluorescent properties in the presence of intracellular ice, enabling effective real-time monitoring during cryo-imaging. Our comprehensive in vitro, in vivo, and ex vivo studies confirm the structural integrity and functional efficacy of the COF, underpinning its potential for clinical use. This development underscores the versatility and transformative potential of COFs in medical imaging, particularly in addressing the unique challenges of oncologic surgery.

## Results and Discussion

### Exfoliation of TG-DFP-COF for Real-Time Cryo-Imaging Applications

**Figure 1:**
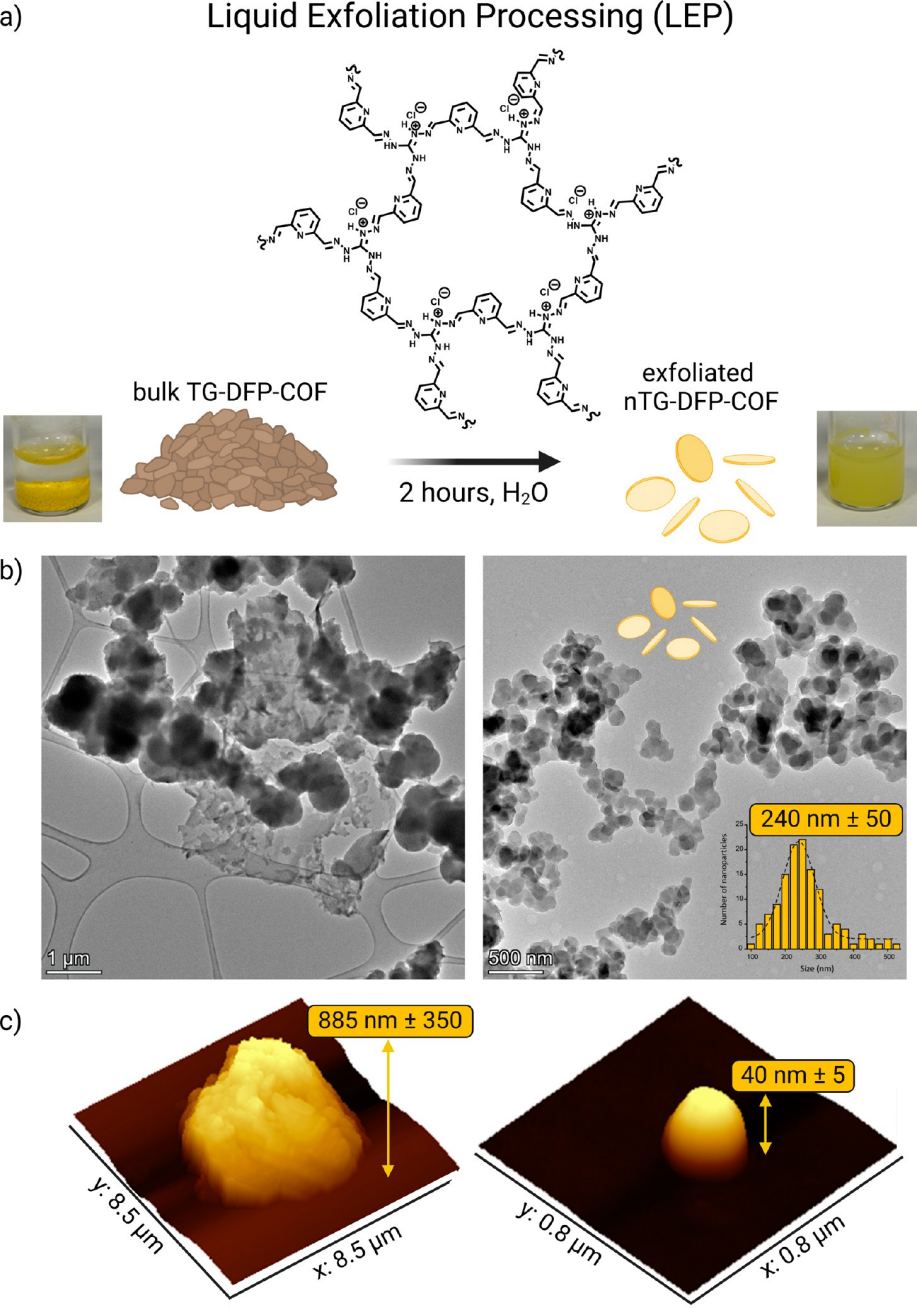
Structure and Characterization of nTG-DFP-COF. (a) Diagram illustrating the liquid exfoliation processing (LEP) used to convert bulk TG-DFP-COF to nanoscale nTG-DFP-COF. The process involves a 2 h treatment in water, transitioning from a bulky to a finely exfoliated structure. Digital images of TG-DFP-COF solution before (left) and after (right) liquid exfoliation (2 mg/mL). (b) High-resolution transmission electron microscopy (HRTEM) images comparing the morphology of bulk TG-DFP-COF (left) with the exfoliated nTG-DFP-COF (right). The right panel includes a size distribution histogram, showing the uniformity and scale of the nanosheets. (c) Atomic force microscopy (AFM) images displaying the topographical contrast between the bulk material (left) and the nanostructured nTG-DFP-COF (right), with a distinct reduction in height and smoother surface profile.

Bulk TG-DFP-COF was synthesized according to previously established method, primarily by the condensation of triaminoguanidinium chloride (TGH.Cl, 8.46 mg, 0.06 mmol) with 2,6-diformylpyridine (DFP, 12.15 mg, 0.09 mmol) in H_2_O/1,4-dioxane (2 mL, Figure S1).^24^ In the FT-IR spectrum of TG-DFP-COF, the lack of a C=O stretching vibration band at 1723 cm^–1^ and a new band at 1629 cm^–1^ indicate the formation of a C=N bond. The absence of the N–H stretching vibration at 3185 cm^–1^, usually linked to the amino group in TGH, further confirms an imine bond formation (Figure S2). For biomedical applications, converting the TG-DFP-COF in bulk form into a nanoscale form while ensuring water solubility and stability was crucial.^17^ To this end, a liquid exfoliation process (LEP) by ultrasonication for 2 h was used to convert TG-DFP-COF in bulk form into nanosheets (nTG-DFP-COF, Figure 1a). This process resulted in 2D nanosheets with an average size of approximately 240 nm ± 50, as verified by TEM (Figures 1b and S3). AFM analysis showed that after liquid exfoliation, the thickness of the sheets was reduced from micrometers to 40 nm, creating uniformly thin and shaped nanosheets (Figures 1c and S4). The resulting nTG-DFP-COF was dispersible in water, making it suitable for biological applications. The translucent solution shows a pronounced Tyndall effect (Figure S5), confirming the presence of highly monodispersed ultrathin nanosheets suspended in water.^17,25^

**Figure 2:**
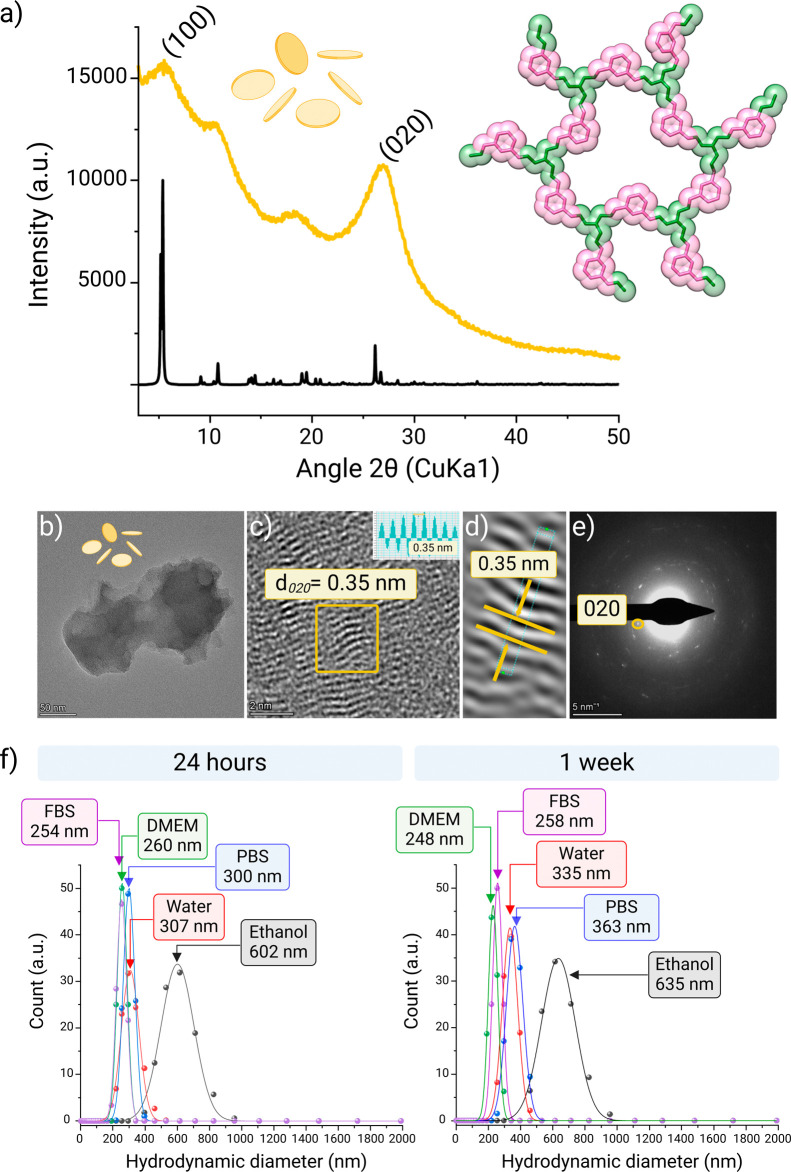
Characterization of nTG-DFP-COF. (a) Experimental (yellow) and calculated (black) PXRD patterns of nTG-DFP-COF, showing the primary diffraction peaks at 2θ = 5.56° (100) and a wide-angle reflection at 2θ = 26.89° (020) indicating the long-range order and π–π stacking of the 2D layers. The inset shows a top view of the space-filling model of the simulated structure observed in the monoclinic space group *P*3. (b) High-resolution transmission electron microscopy (HRTEM) of nTG-DFP-COF with nanosheets of about 240 nm in size. (c) Lattice fringes HRTEM image and (d) their reconstruction: The images show continuous and consistent lattice fringes with a *d*-spacing of 0.35 nm and are in close agreement with the wide-angle PXRD data. (e) Selected area electron diffraction (SAED) shows the crystalline nature of nTG-DFP-COF with distinct electron diffraction spots corresponding to the d_003_ plane with a *d*-spacing of 0.34 nm. (f) Stability analysis of nTG-DFP-COF in various solutions over time. Hydrodynamic diameter distributions of nTG-DFP-COF nanosheets suspended in different solvents: ethanol, water, phosphate-buffered saline (PBS), fetal bovine serum (FBS), and Dulbecco’s modified eagle medium (DMEM). Measurements were performed at two intervals, 24 h (left panel) and 1 week (right panel).

Powder X-ray diffraction (PXRD) measurements were recorded to confirm the crystallinity of nTG-DFP-COF (Figure 2a). The PXRD pattern shows the most intense diffraction peaks at 2θ = 5.56° (100), indicating the long-range order in the framework. In the wide-angle region, the reflection at 2θ = 26.89° associated with the (020) plane suggests the adjacent π–π stacking of the 2D layers. Following the principles of reticular chemistry, crystal structure models were constructed based on the geometry of the building blocks; the COF structure was observed in the monoclinic space group *P*3, following a honeycomb (**hcb**) layered arrangement. The completed Pawley refinement supports the obtained unit cell with lattice parameters of *a* = 19.419 Å, *b* = 6.809 Å, and *c* = 19.493 Å (beta = 122.47°).

The crystalline nature of nTG-DFP-COF was further evidenced by lattice-resolution TEM images, which showed consistent and continuous lattice fringes throughout the COF structure (Figure 2b,c). With lattice spacings of 0.35 nm, these fringes corresponded well to the *d*_020_ plane interlayer distances and the π–π stacking, closely aligning with the experimental wide-angle PXRD data at 0.34 nm (Figure 2d). Furthermore, the selected area electron diffraction (SAED, Figure 2e) demonstrates the well-crystallized feature of nTG-DFP-COF and has distinct electron diffraction spots that fit well with the *d*_020_ plane with a *d*-spacing of 0.34 nm.

Given that our material is designed for use at extremely low temperatures, we evaluated possible structural changes in nTG-DFP-COF under freezing conditions using TEM, AFM, and Variable Temperature (VT)-PXRD. TEM and AFM analyses showed no changes in the morphology or dimensions of nTG-DFP-COF nanosheets after freezing to −40 °C, confirming the robust structural stability (Figure S6). Additionally, VT-PXRD experiments conducted before, during, and after freezing showed consistent peak positions and intensities at different temperatures, indicating that the crystal lattice remains intact without any changes in lattice parameters (Figure S7). Overall, these results confirm the structural integrity and stability of nTG-DFP-COF and emphasize its suitability for cryogenic applications.

The aqueous solutions of the exfoliated nTG-DFP-COF nanosheets show stability and dispersibility, with no signs of precipitation over time. At a neutral pH of 7.4 in water, the hydrodynamic diameter of these nanosheets was measured to be 295 nm with a low polydispersity index (PDI) of 0.1 compared to the bulk material, indicating a reduced and uniform particle size distribution (Figure S8). Larger particle sizes are generally observed in DLS than in TEM (240 nm) due to hydration layers and the potential for aggregation in solution, reflecting the interaction of the nanosheets with the solvent environment.^26,27^ The improved stability compared to the bulk material can be attributed to the significant decrease in the ζ-potential of nTG-DFP-COF, which shifted from −4.8 ± 1.8 mV in the bulk material to −15.6 ± 0.3 mV for the exfoliated nanosheets. The stronger negative charge on the nanosheets promotes better interparticle repulsion and thus contributes to their stability. We then measured the hydrodynamic diameter of the nanosheets in different solvents, including ethanol, PBS, FBS, and DMEM, after 24 h and 1 week, as shown in Figure 2f. Initially, the stability in the different solvents varied, with DMEM showing the highest stability and ethanol having the lowest stability. The size generally increases slightly over 1 week, except in DMEM, where it decreases slightly, indicating strong colloidal stability. These results show that the nanosheets aggregate only minimally and are very stable, especially in physiological environments, emphasizing their suitability for biomedical applications.

### Temperature-Dependent Optical and Fluorescence Properties of nTG-DFP-COF for Enhanced Cryo-Imaging Applications

To utilize nTG-DFP-COF for cryo-imaging, we studied the electronic properties of the exfoliated nanosheets by UV–visible and steady-state fluorescence spectroscopy as a function of temperature.

**Figure 3:**
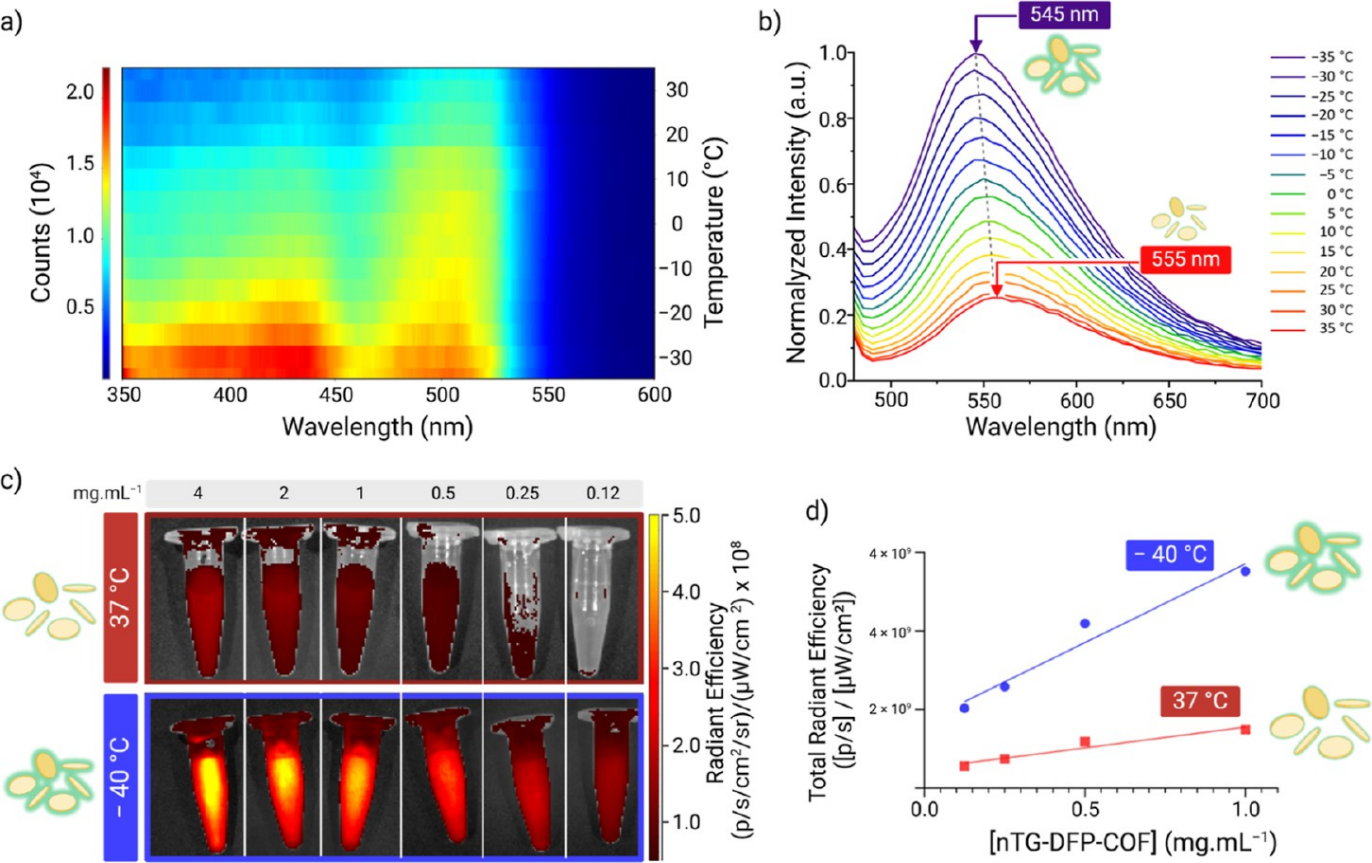
Temperature-dependent optical and fluorescence properties of nTG-DFP-COF. (a) Two-dimensional heatmap of variable temperature solid-state DR-UV–visible spectra of nTG-DFP-COF: this heatmap shows the variation in absorption of nTG-DFP-COF over a spectrum of wavelengths (350 to 600 nm) and temperatures (from −30 to 30 °C). (b) Variable temperature solid-state fluorescence spectra of nTG-DFP-COF over a wide temperature range from −35 to 35 °C, showing the thermally modulated fluorescence intensity of the material (λ_ex/em_ = 400/540 nm). Fluorescent phantom imaging at different concentrations and temperatures: (c) IVIS-based images showing the fluorescence of nTG-DFP-COF at different concentrations, compared at physiological temperature (37 °C) and in the frozen state (−40 °C), to illustrate the changes in fluorescence intensity (λ_ex/em_ = 465/540 nm). (d) Quantitative analysis of fluorescence intensity: Plots of fluorescence image intensities versus concentrations of nTG-DFP-COF used to calculate the weight radiant efficiencies, highlighting the relationship between concentration and fluorescence efficiency under different thermal conditions.

We measured the absorbance wavelength change on nTG-DFP-COF suspended in acetonitrile in a temperature range from −30 to 30 °C. The data presented in Figure 3a indicate that absorbance increases as the temperature decreases, reaching a peak intensity at 420 nm when the temperature is at −30 °C. These data provide important insights into the thermal stability and responsive behavior of nTG-DFP-COF under various thermal conditions, underscoring its potential for temperature-sensitive applications. We also measured the solid-state luminescence of nTG-DFP-COF over a broad temperature range from −35 to 35 °C. At –35 °C, nTG-DFP-COF exhibits strong green fluorescence (λ_em_ = 545 nm, Figures 3b, S9 and S10) when excited at 400 nm, highlighting its robust photophysical properties. The inherent fluorescence of nTG-DFP-COF is enhanced at lower temperatures, likely due to the stabilization of hydrogen bonds within the COF networks, which reduces the nonradiative relaxation pathways and increases the emission intensity. As the temperature increases, the disruption of the weak hydrogen bonds leads to a significant reduction in emission intensity, decreasing by a factor of 5.^24,28^ The results demonstrate that the photoluminescence behavior is strongly temperature-dependent, suggesting that higher temperatures may increase structural flexibility, thereby promoting nonradiative relaxation processes.

To evaluate the optical properties of nTG-DFP-COF as fluorescent bioimaging probes in water, we used the IVIS spectrum imaging system. Phantom imaging was performed with nTG-DFP-COF samples diluted in water at different concentrations, kept at 37 °C and in a frozen state (−40 °C, verified with a thermal imaging camera, Figure S11). A 465 nm bandpass excitation filter and a 540 nm emission filter were used to selectively capture fluorescence images (Figure 3c). The analysis revealed that the fluorescence intensities of the nTG-DFP-COF samples increase with concentration. All fluorescence intensities were normalized to photons/second/centimeter^2^/steradian (p/s/cm^2^/sr), with background intensities subtracted for accuracy. At 37 °C, the increase in fluorescence intensity was moderate and linear with concentration. In contrast, the increase was more pronounced in the frozen state and followed a linear trend. Using the data shown in Figure 3d, the average radiant efficiencies were quantified at 37 °C and −40 °C. The slopes of the radiant efficiency curves were 1.9 × 10^9^ and 6.0 × 10^9^ p/s/cm^2^/sr per mg for the samples at 37 °C and in the frozen state, respectively. This significant increase in efficiency in the presence of ice crystals suggests that ice formation does not affect the fluorescence intensity of the probe at low temperatures but can even enhance it. These results are consistent with our steady-state fluorescence studies (Figure 3a,b), which confirm that the structural integrity and fluorescence response of nTG-DFP-COF are maintained or even improved under frozen conditions.

To investigate the effect of temperature on the binding affinity of water molecules to the nTG-DFP-COF, we performed molecular dynamics (MD) simulations on a segment of the framework at two distinct temperatures, −40 and 20 °C. These simulations used the xTB-GFN2 method, which combines semiempirical and tight binding quantum mechanical approaches, along with an implicit water solvent model.^29^ To further investigate the hydrogen bonding interactions, we introduced 15 explicit water molecules into our model, enabling a detailed analysis of the interactions between the water molecules and the nTG-DFP-COF (Figure S13a). The simulation system is confined to a sphere with a radius of 10.5 Å by a repulsive potential to prevent the escape of water molecules. The MD simulations are carried out for 200 ps with a time step of 2 fs using an extended tight binding (XTB) program,^30^ and the trajectories are collected every 10 fs. The first 10 ps of the trajectory was discarded for equilibration, and the final 190 ps trajectory was used for analysis. We computed the radial distribution function (RDF) between the oxygen atoms of water and the hydrogen atoms of nTG-DFP-COF to illustrate different binding affinity at different temperatures (Figure S13b). In the radial distribution function (RDF), we find that the peak intensity is significantly higher at −40 °C than at 20 °C, indicating that the binding affinity between water molecules and nTG-DFP-COF increases as the temperature decreases (Figure S13b). Moreover, the peak position shifts by 0.07 Å at 20 °C compared to −40 °C, indicating stronger interactions between water molecules and nTG-DFP-COF at the lower temperature (Figure S13b). Integration of the RDFs reveals that at −40 °C, there are, on average, 2.26 water molecules in the first solvation shell (within a 3 Å distance), whereas at 20 °C, there are only 1.45 water molecules. These results show that temperature significantly affects the binding affinity of water molecules to nTG-DFP-COF.

### Mechanisms of Temperature-Dependent Fluorescence Enhancement in nTG-DFP-COF

The fluorescence enhancement mechanism of nTG-DFP-COF at low temperatures was investigated using variable-temperature (VT) techniques, including NMR, FTIR, and XPS.

**Figure 4:**
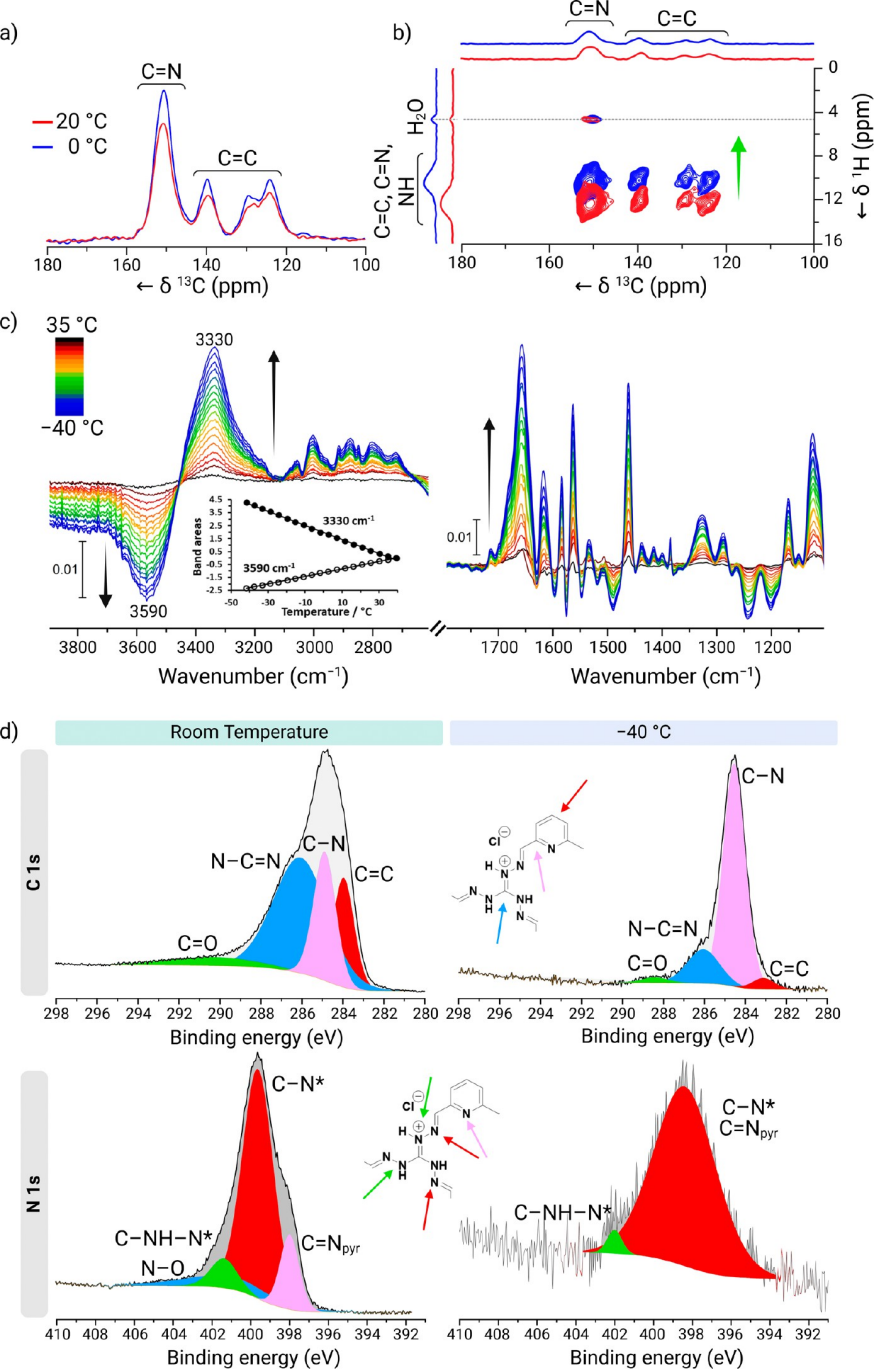
Analysis of nTG-DFP-COF Across Variable Temperatures. (a) ^13^C CP/MAS solid-state NMR Spectra: overlay of the one-dimensional ^13^C CP/MAS solid-state NMR spectra of nTG-DFP-COF at 20 °C (red lines) and at 0 °C (blue lines), showing the stability of the chemical shifts upon temperature reduction. (b) ^1^H–^13^C HETCOR solid-state NMR spectra: overlay of two-dimensional ^1^H–^13^C HETCOR solid-state NMR spectra of nTG-DFP-COF at 20 °C (red contours) and 0 °C (blue contours). Notable upfield changes in proton chemical shifts are indicated by a green arrow, suggesting stronger hydrogen bonding at lower temperatures. (c) Temperature-dependent IR spectra across a temperature range from −40 to 35 °C: evolution of the IR spectra of nTG-DFP-COF with temperature changes. Spectra were recorded for nTG-DFP-COF (1 wt % in KBr) before and after activation under vacuum at 40 °C for 12 h to remove residual adsorbed water. The spectra subtracted from the baseline recorded at 40 °C highlight changes due to temperature variations. Inset shows the evolution of band areas centered at 3330 cm^–1^, assigned to N–H hydrogen bonding bands. (d) XPS Analysis at room temperature and −40 °C: XPS C 1s (top) and N 1s (bottom) spectra of nTG-DFP-COF comparing room temperature and −40 °C, illustrating the changes in the electronic environment and bonding properties under extreme temperature conditions.

Variable temperature solid-state NMR experiments were first performed. Figure 4a presents the one-dimensional ^13^C CP/MAS solid-state NMR spectra of nTG-DFP-COF at 20 °C (red lines) and 0 °C (blue lines). The spectra predominantly show peaks of imine (145 to 155 ppm) and aromatic carbons (115 to 145 ppm), with no significant shifts in carbon chemical shifts observed upon cooling. This stability in carbon chemical shifts can be attributed to the low sensitivity of ^13^C nuclei to noncovalent interactions and the inherently broad ^13^C signals in solid-state NMR, which may obscure subtle changes. However, the increased ^13^C CP/MAS signal at 0 °C indicates reduced molecular motion and increased rigidity of the framework compared to room temperature. The enhancement of ^13^C CP/MAS signal intensity at 0 °C reflects the increased efficiency of cross-polarization (CP), as molecular rigidity strengthens dipolar couplings between abundant nuclei (^1^H) and ^13^C nuclei. The reduction in dynamic disorder at lower temperatures allows for more effective magnetization transfer, leading to improved polarization transfer efficiency and enhanced signal intensity. These observations confirm that the nTG-DFP-COF framework becomes more rigid and exhibits reduced molecular motion at lower temperatures.^31,32^

Because ^1^H chemical shifts are more sensitive to hydrogen bonding, we monitored changes in proton chemical shifts by two-dimensional ^1^H–^13^C HETCOR (heteronuclear correlation) solid-state NMR experiments at 20 and 0 °C (Figure 4b). The spectra at both temperatures show correlation peaks between aromatic, imine, and amine protons with imine and aromatic carbons. Additionally, peaks corresponding to water molecules (∼4.6 ppm) interacting with imine carbon atoms (∼150 ppm) are observed, indicating the close proximity of water molecules to the guanidine moiety of the imine carbon atoms. At 0 °C, significant upfield chemical shifts (∼2 ppm) for protons were observed, suggesting that noncovalent interactions become stronger and more ordered as the temperature decreases.

The nTG-DFP-COF framework, consisting of planar, electron-rich guanidine units with multiple nitrogen atoms, exhibits exceptional hydrogen bonding capabilities.^33^ As the temperature decreases, these noncovalent interactions become stronger, leading to increased proton shielding and corresponding upfield shifts in solid-state NMR.^31^ It has been shown that at low temperatures, the cationic guanidinium groups in arginine can self-assemble through hydrophobic interactions, forming stacked pair configurations. This assembly helps to offset the anticipated Coulomb repulsion.^32^ The resulting reduction in molecular vibrations and rotations at lower temperatures leads to a more rigid structure that enhances the shielding effects for protons involved in noncovalent interactions such as π–π stacking or hydrogen bonding. As a result, nTG-DFP-COF shows enhanced fluorescence at lower temperatures due to the reduced nonradiative decay pathways. At higher temperatures, molecular vibrations and rotations dissipate energy nonradiatively, reducing fluorescence intensity. At lower temperatures, suppression of these processes allows more energy to be emitted as fluorescence. Additionally, enhanced noncovalent interactions improve electronic conjugation within the COF framework, further boosting fluorescence.

Low-temperature transmission FT-IR analysis was also performed on nTG-DFP-COF in a temperature range of −40 to 35 °C, with the sample diluted in KBr to prevent the saturation of the structural bands (Figure 4c). The self-supported pellet was first activated under vacuum at 40 °C for 12 h to remove residual adsorbed water. The results show that at low temperatures, the mobility within the nTG-DFP-COF structure decreases, leading to stronger hydrogen bonding between the N–H groups and other electronegative atoms, such as nitrogen, within the COF structure. This increased hydrogen bonding leads to a contraction of the COF framework, effectively densifying the vibrational modes and decreasing the flexibility of the structure.^34−36^ This densification is reflected by an increase in the intensity of the structural vibrational bands in the range of 3100–2700 cm^–1^ and 1700–1100 cm^–1^. Furthermore, stronger hydrogen bonding elongates the N–H bond,^37^ which reduces its bond strength and consequently lowers the vibrational energy. This change is manifested by the presence of an isosbestic wavenumber at 3445 cm^–1^, demonstrating a shift of the N–H bond vibration from 3590 to 3300 cm^–1^ with a symmetrical evolution of both vibrational bands with the temperature (Figure 4c). The densification of the structure explains the enhancement of both absorption/excitation and photoluminescence (PL) of nTG-DFP COF with decreasing temperature. The enhanced PL efficiency of nTG-DFP-COF at lower temperatures may be due to the COF’s reduced structural flexibility, which limits nonradiative relaxation pathways. This results from improved π–π interactions, reduced nonradiative decay, stabilized excited states, and confined excitons, leading to increased PL intensity as the temperature drops.

The X-ray photoelectron spectroscopy (XPS) analysis of C 1s and N 1s levels in nTG-DFP-COF provides critical insights into its chemical structure and bonding environment, highlighting its significant temperature-dependent properties (Figure 4d and Table S2). At room temperature, the C 1s spectrum features distinct peaks at 283.95 eV (C=C), 284.90 eV (C–N), and 286.13 eV (N–C=N), constituting 19.0%, 24.0%, and 51.25% of the spectrum, respectively. These peaks underscore a highly ordered aromatic backbone essential for the COF’s structural integrity, ensuring robustness under ambient conditions.^38^ Additionally, the carbon–nitrogen bonds not only enhance the electronic properties but also contribute to the COF’s fluorescence capabilities.^39^ The low residual standard deviation of 0.9 across the material suggests a consistent and uniform chemical environment.^40^ At –40 °C, the primary peak at 284.55 eV increases to 77.19%, indicating the aromatic backbone’s stability in colder conditions. Minor shifts and slight broadening of the secondary peak at 286.05 eV, accounting for 16.87% of the spectrum, suggest enhanced hydrogen bonding and reduced thermal vibrations, thus reinforcing the structure.^41^ The increased residual standard deviation to 1.3 at this temperature points to a more complex electronic environment, likely a result of cryogenic stabilization.^42^ In the N 1s spectrum, the room temperature profile decomposes into three primary peaks at 397.99 eV (C=N Pyridine), 399.64 eV (C–N*), and 401.4 eV (C–NH–N*), contributing 13.6%, 68.11%, and 10.8%, respectively (Figure 4d and Table S3).^43^ These peaks play a vital role in the electronic stability and fluorescence enhancement of the COF.^39,44^ An additional weaker peak around 401.2 eV, likely representing protonated or oxidized nitrogen species, indicates environmental interactions.^45^ However, at −40 °C, the spectrum simplifies significantly; the C=N Pyridine and C–N* peaks merge into a broad peak at 398.45 eV, representing 96.94% of the spectrum, signifying enhanced nitrogen stabilization under cryogenic conditions. The diminished secondary peak at about 402.1 eV, contributing only 3.06%, underscores stronger hydrogen bonding and reduced thermal vibrations.^41,46^ The absence of the peak for protonated or oxidized nitrogen at this temperature suggests stabilization of these species within the cryogenic framework.^47^

This comprehensive analysis using solid-state NMR, VT-FTIR, and XPS demonstrates the exceptional stability and functional adaptability of nTG-DFP-COF across various temperature ranges. The COF retains its structural integrity even under cryogenic conditions, exhibiting significant enhancements in hydrogen bonding and electronic stabilization. The fluorescence enhancement observed in nTG-DFP-COF at cryogenic temperatures arises from several interrelated factors that influence its photophysical properties. At low temperatures, the significant reduction in thermal energy decreases molecular vibrations and motions within the COF matrix. This reduction in molecular motion facilitates the formation and stabilization of hydrogen bonds, leading to a more rigid molecular structure. The increased rigidity effectively minimizes the nonradiative relaxation pathways, which are typically driven by molecular motions such as rotations and vibrations. With these nonradiative pathways becoming less active, the material predominantly favors radiative decay processes, resulting in increased photon emission rather than energy dissipation as heat. This shift significantly enhances fluorescence intensity at lower temperatures. This direct link between increased molecular rigidity and a shift toward predominantly radiative relaxation processes underlines the unique photophysical characteristics of nTG-DFP-COF in cryogenic conditions.

### In Vitro Evaluation of nTG-DFP-COF as a Fluorescent Probe in Cancer and Normal Cell Lines

In vitro studies were performed on three different cell lines—HEK-293 (noncancerous), HeLa (cervical cancer), and U251-MG (glioblastoma)—to evaluate nTG-DFP-COF as a biocompatible fluorescent probe for cryo-imaging. HEK-293 cells were used as a control to assess the probe’s response in normal cells, while HeLa and U251-MG cell lines were used to evaluate the efficacy and specificity of the probe in different cancer types. These experiments validate the suitability of nTG-DFP-COF for real-time cryo-imaging in both normal and cancerous cells.

The material showed minimal cytotoxicity in all cell lines at concentrations up to 1 mg/mL after 48 h of incubation, confirming its excellent biocompatibility and potential for cellular labeling (Figure S14). Additionally, cytotoxicity assessments were further confirmed through MTS and lactate dehydrogenase (LDH) assays conducted after 24 h of exposure to different concentrations of nTG-DFP-COF (Figure S15). The MTS assay indicated normal mitochondrial function, suggesting healthy cellular metabolism, while the LDH assay showed minimal release of LDH, indicating that cell membranes were largely intact. Long-term toxicity and cell proliferation studies tracked the growth of HeLa, U251-MG, and HEK-293 cells over a three-day period and analyzed daily cell counts and population doubling times (PDTs), which revealed no significant differences between treated and control groups, confirming that nTG-DFP-COF does not negatively affect cell proliferation (Figure S14 and Table S4). To evaluate the biocompatibility and immunotoxicity of our material designed for bloodstream penetration, we conducted a hemolysis assay on human erythrocytes.^48−50^ The hemolysis rates (HR) were below 3%, indicating nonhemolytic properties as per ASTM F 756-08 standards, which set a threshold of <5% (Figure S16). These results confirm that nTG-DFP-COF is biocompatible and does not induce immunotoxicity in human blood erythrocytes, likely due to the negatively charged surface of our nanoparticles that reduces hemolytic effects.^51−54^ Altogether, these results underscore the probe’s suitability for long-term cellular studies and its potential for safe biomedical applications.

**Figure 5:**
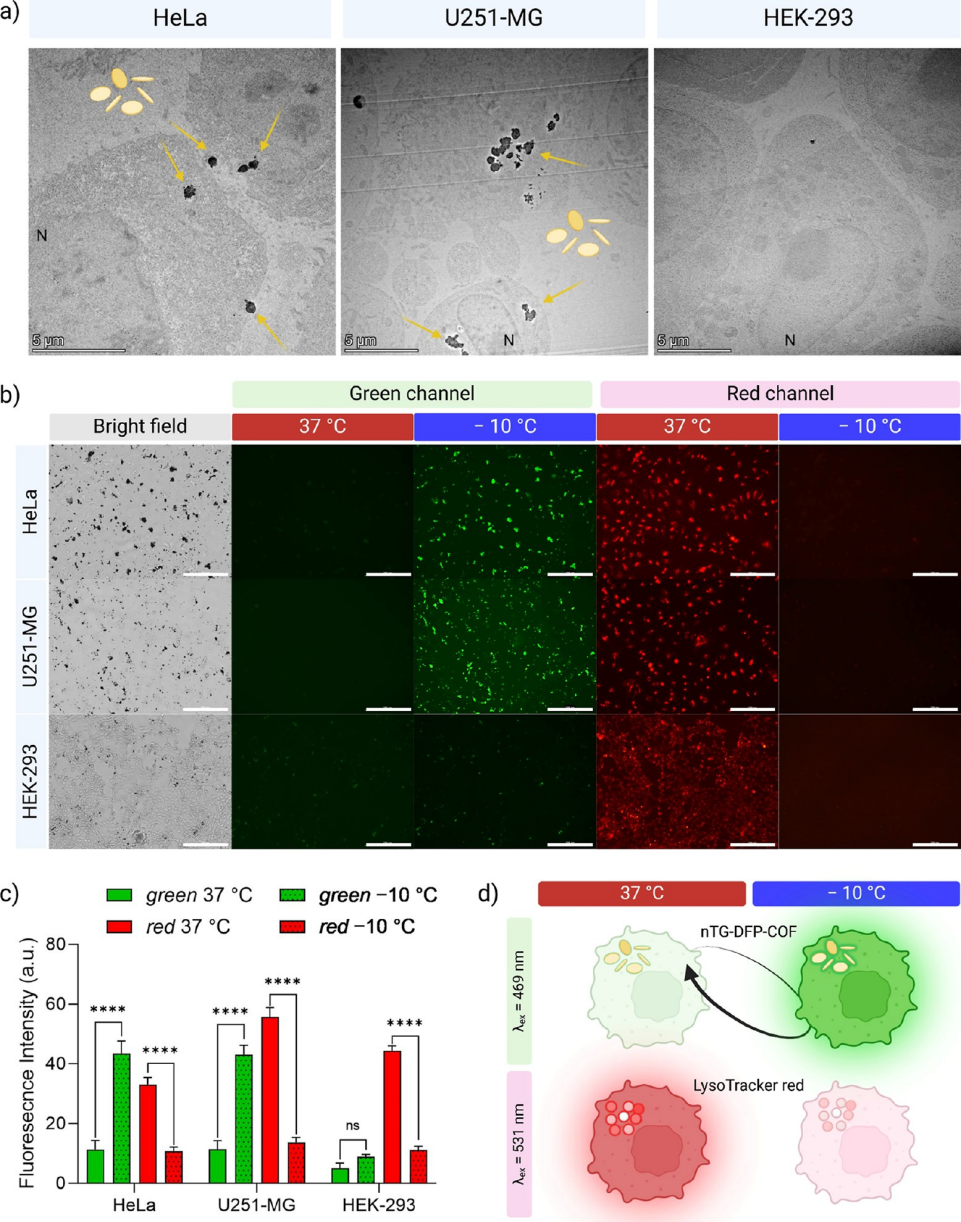
Intracellular uptake and fluorescence response of nTG-DFP-COF in cancerous and noncancerous cell lines. (a) Transmission electron microscopy (TEM) images of HeLa, U251-MG, and HEK-293 cells after 24 h of incubation with nTG-DFP-COF (yellow arrows) at 10 μg/mL. The images show preserved cellular integrity with minimal debris, highlighting the biocompatibility of nTG-DFP-COF. (b) Bright-field and Fluorescence microscopy images of HeLa, U251-MG, and HEK-293 cells costained with nTG-DFP-COF and LysoTracker red, under 469 and 531 nm light, at temperatures of 37 and −10 °C. Scale bar: 200 μm (c) graph depicting the fluorescence intensity changes for HeLa, U251-MG, and HEK-293 cells at 37 and −10 °C. At −10 °C, the increase in green fluorescence intensity of nTG-DFP-COF in HeLa and U251-MG is contrasted with the decrease in red fluorescence of LysoTracker red, illustrating the different stability and response to temperature changes. Significance levels: *p < 0.05, **p < 0.01, ***p < 0.001, ****p < 0.0001, ns: not significant. (d) Schematic representation of the fluorescence activation mechanism of nTG-DFP-COF at different temperatures and its cellular localization in green and red channels, showing the selective activation of nTG-DFP-COF in cancer cells (HeLa, U251-MG), highlighting its potential for improved specificity of cryo-imaging.

We investigated the intracellular uptake of nTG-DFP-COF by HeLa, U251-MG, and HEK-293 cells using TEM after 24 h of incubation at a concentration of 10 μg/mL (Figure 5a). TEM analyses showed that the structural integrity of all cell lines was largely preserved with minimal evidence of cell debris, indicating that nTG-DFP-COF is nontoxic and does not affect cell function (see Figure S17 for comparison with control cells). In HeLa and U251-MG cells, significant amounts of nTG-DFP-COF were detected both inside the cells and on their surface, indicating active uptake (Figures 5a and S18). Initially, the nTG-DFP-COF nanosheets come into contact with the cell membrane, followed by their engulfment. This progresses into deeper invaginations of the cell membrane, indicating early endosomal formation. Subsequently, the nanosheets are transported into large vacuoles, primarily located near the perinuclear region and in the cytoplasm. This sequence from plasma membrane engagement to endosomal localization highlights the active internalization process of nTG-DFP-COF in these cancer cell lines (Figures 5a and S18). Conversely, HEK-293 cells showed lower uptake of nTG-DFP-COF, suggesting it selectively interacts with cancer cells rather than noncancerous cells.^15,55−57^ nTG-DFP-COF nanosheets target cancer cells effectively by exploiting their altered physiological pathways.^58,59^ These nanosheets use their size and surface charge to enhance entry into cells through endocytosis, a process more pronounced in cancer cells because of their higher metabolic activity.^60−65^ This leads to more internalization in cancer cells than in normal cells, potentially improving therapeutic outcomes. nTG-DFP-COF is preferentially taken up by cancer cells over HEK-293 cells, attributed to variations in cell surface charge, membrane thickness, and turnover rates, underscoring their selective targeting properties.

To distinguish passive from active internalization mechanisms of nTG-DFP-COF in HeLa and U251-MG cells, we analyzed the fluorescence signals under varying cell incubation temperatures (4 °C versus 37 °C). This approach takes advantage of the fact that nanomaterial entry into cells can occur via energy-independent passive mechanisms, such as direct translocation, or via energy-dependent active mechanisms, such as endocytosis (Figures S19 and S20).^66^ In cells incubated at 4 °C, fluorescence was significantly weaker, indicating minimal passive uptake. In contrast, fluorescence significantly increased at 37 °C (HeLa: 90.1%, U251-MG: 88.5%), indicating that active mechanisms, predominantly endocytosis, are responsible for the uptake of nTG-DFP-COF. Further, subcellular localization was assessed in HeLa and U251-MG cells treated with nTG-DFP-COF for 4 h. Confocal microscopy and costaining with far-red organelle-specific markers revealed significant colocalization of nTG-DFP-COF with cell membranes and lysosomes, suggesting active internalization and intracellular transport (Figures S21 and S22). This localization supports the hypothesis that nTG-DFP-COF enters cells primarily by endocytosis. These results, supported by TEM data, confirm the effective intracellular transport and localization of nTG-DFP-COF by endocytosis and highlight its potential for targeted bioimaging applications.

Next, we demonstrate that nTG-DFP-COF has a specific freezing-induced turn-on function in cancer cells. Fluorescence micrographs (Figure 5b) show HeLa, U251-MG, and HEK-293 cells incubated with nTG-DFP-COF (10 μg/mL, λ_ex/em_ = 469/525 nm) and LysoTracker Red (250 nM, λ_ex/em_ = 531/593 nm), both at room temperature and in frozen condition (−10 °C, using cold ethanol, Figure S12). LysoTracker Red was chosen for comparison because, as a traditional organic dye, it faces challenges under cryo-imaging conditions, such as dye aggregation and fluorescence quenching due to ice formation. In addition, although LysoTracker Red passively diffuses into cells and accumulates in the acidic organelles of all cell types, it does not selectively target cancer cells.^67^ By comparing the luminescence responses of LysoTracker and nTG-DFP-COF at different temperatures and in different cell types, we aim to evaluate the selective cancer-targeting and turn-on properties of nTG-DFP-COF.

At room temperature, HeLa and U251-MG cells costained with nTG-DFP-COF and LysoTracker red emit weak green fluorescence under 469 nm light and show strong red fluorescence under 531 nm light (Figure 5b). After freezing, the fluorescence intensity of nTG-DFP-COF in HeLa and U251-MG cells increases significantly. In contrast, the fluorescence intensity of LysoTracker red decreases considerably due to ice formation in the cells (Figure 5b).^8^ The fluorescence intensity of nTG-DFP-COF increases by 3.8- and 3.7-fold in HeLa and U251-MG cells, respectively, while that of LysoTracker red decreases by almost 3.0- and 3.7-fold in HeLa and U251-MG cells, respectively (Figure 5c). This decrease in fluorescence of the conventional dye is mainly attributed to aggregation-induced quenching and low photostability.^68,69^ These results clearly show that nTG-DFP-COF has a turn-on feature that contrasts with the turn-off property typical of conventional dyes (Figure 5d).^8,70^ This turn-on property of nTG-DFP-COF provides lower background interference and a higher signal-to-noise ratio, which improves real-time imaging capabilities.^71,72^

Another essential prerequisite for real-time imaging during cryosurgery is distinguishing tumor tissue from the surrounding normal tissue.^2^ Using the noncancerous HEK-293 cells, we investigated the imaging cancer specificity of nTG-DFP-COF. HEK-293 cells show weak green fluorescence at room temperature when stained with nTG-DFP-COF and strong red fluorescence when stained with LysoTracker Red. LysoTracker Red passively diffuses into cells, both malignant and nonmalignant.^67^ This dye does not selectively target cancer cells. In contrast, due to their higher endocytosis rate, HeLa and U251-MG cells show green fluorescence under the same conditions (Figure 5b).^55^ This demonstrates that nTG-DFP-COF selectively targets cancer cells. To further evaluate the utility of nTG-DFP-COF for selective imaging of cancer cells after freezing, we observed that green and red fluorescences are particularly weak in frozen HEK-293 cells. In contrast, frozen HeLa and U251-MG cells exhibit strong green emission (Figure 5b-c). The selective behavior of nTG-DFP-COF was confirmed with cancer cells at room temperature and subfreezing temperatures (Figures 5b,c), highlighting the excellent specificity of the probe for cancer cells. This clear contrast between normal and cancer cells enables precise cryo-imaging planning (Figure 5d).

### In Vivo Evaluation of nTG-DFP-COF Biosafety

**Figure 6:**
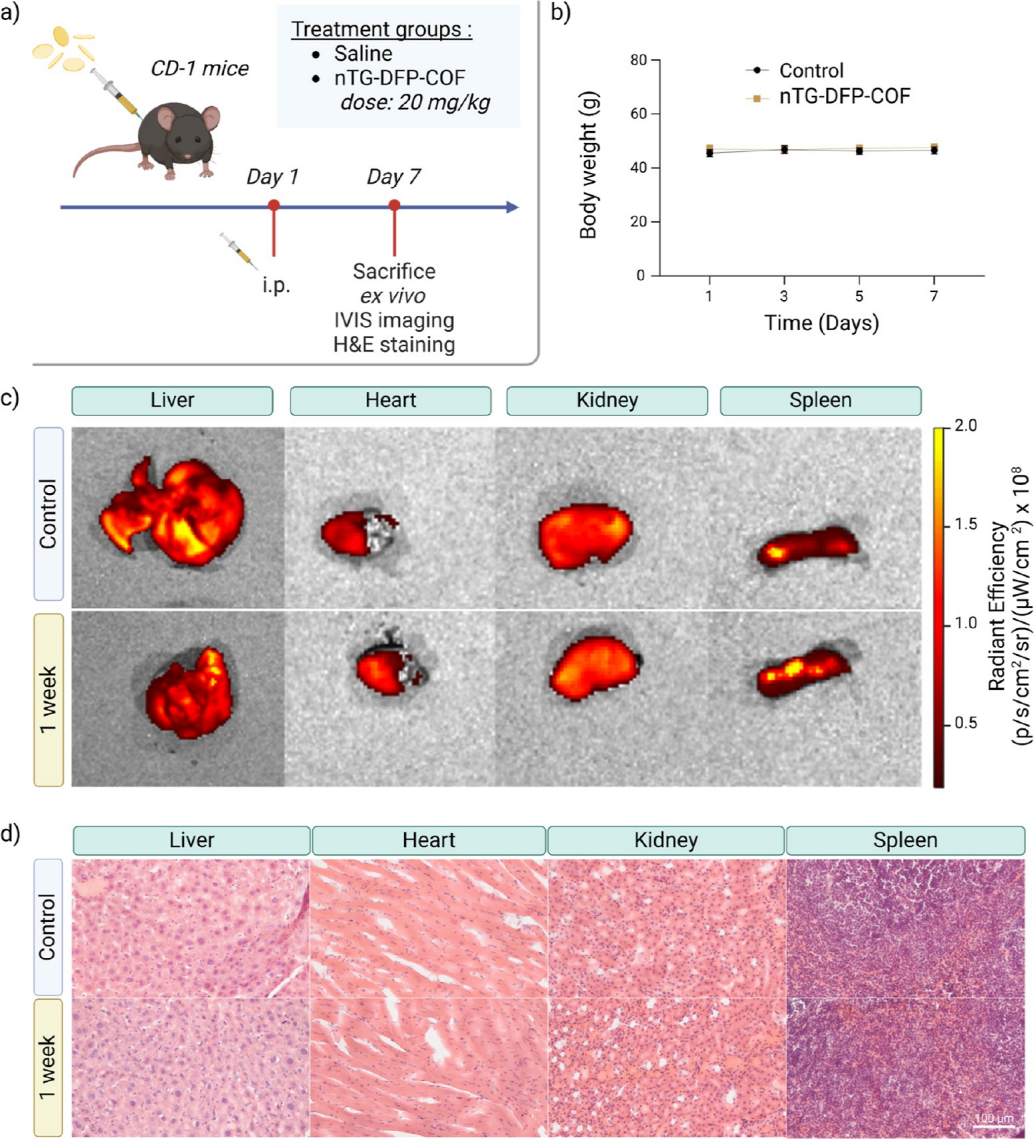
In vivo toxicity and biocompatibility assessment of nTG-DFP-COF in CD-1 Mice. (a) Experimental overview: schematic representation of administration of nTG-DFP-COF by a single intraperitoneal (i.p.) injection in CD-1 mice, followed by detailed analysis of organ tissues after 7 days. (b) Body weight of CD-1 mice treated with either saline (control) or nTG-DFP-COF at a dose of 20 mg/kg, monitored for 7 days postinjection. (c) Ex vivo Bioluminescence imaging: images of key organs (liver, heart, kidney, spleen) obtained using the IVIS spectrum imaging system one-week after administration of nTG-DFP-COF at a dose of 20 mg/kg. (d) Histopathological analysis: microscopic examinations of major organs (liver, heart, kidney, spleen) to assess cellular and structural integrity after administration of nTG-DFP-COF administration. These panels confirm the overall biocompatibility and safety of nTG-DFP-COF.

To assess the biosafety of nTG-DFP-COF in vivo, we performed comprehensive toxicity studies in CD-1 mouse model.^73^ Mice received a single intraperitoneal injection of nTG-DFP-COF at a dose of 20 mg/kg, while a saline-injected group served as a control (Figure 6a). Over an observation period of 7 days, there were no significant changes in body weight, behavior, or survival rates in the treated mice, indicating no acute toxicity (Figure 6b). The injection sites showed no signs of irritation, and behavioral monitoring confirmed no distress in the treated animals. Seven days after treatment, we sacrificed the animals and harvested major organs for analysis. Ex vivo bioluminescence imaging and subsequent pathohistological evaluations, including H&E staining of organs, showed no significant differences or morphological changes compared to controls (Figures 6c,d). These results indicate effective clearance of nTG-DFP-COF and confirm the absence of systemic toxicity, confirming the compound’s biocompatibility and safety in an acute toxicity model.^74^

### Ex Vivo Evaluation of nTG-DFP-COF for Tumor Cryo-Imaging

To assess the utility of nTG-DFP-COF for cryo-imaging, we conducted an ex vivo study using an orthotopic U251-MG glioblastoma model. Tumors in mice reached approximately 75–100 mm^3^ before the mice were humanely euthanized and the tumors excised for analysis. Each tumor was injected with either nTG-DFP-COF ([nTG-DFP-COF] = 20 mg/kg, 200 μL) or PBS as a control. Following imaging at 37 °C, the tumors were flash-frozen in liquid nitrogen for 10 min to mimic cryosurgery and assess the response of nTG-DFP-COF in a frozen state (−40 °C, verified with a thermal camera, Figure S25), the necessary temperature to destroy malignant cells in vivo.^75^

**Figure 7:**
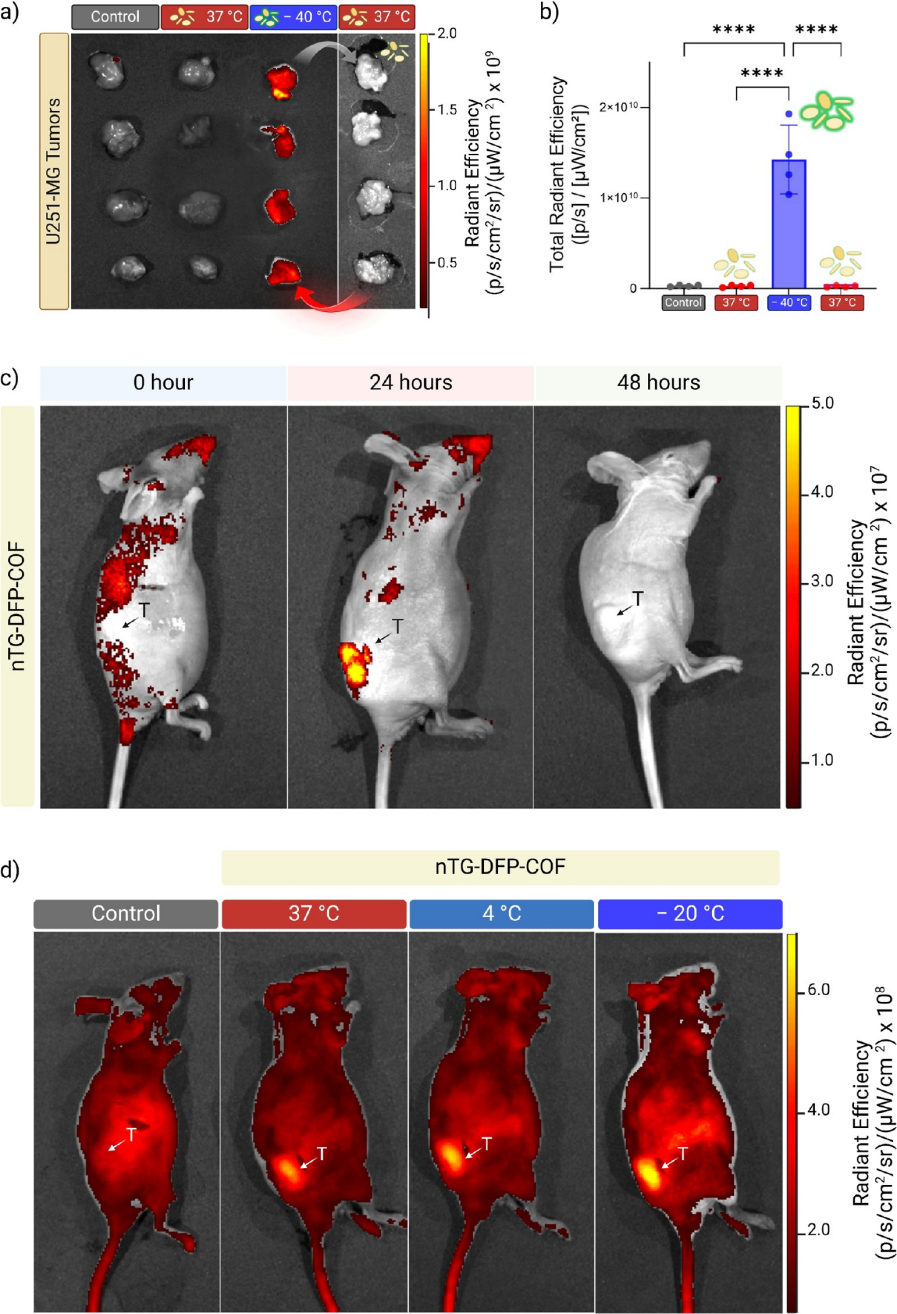
Evaluation of nTG-DFP-COF as targeted cryo-imaging probe in U251-MG Glioblastoma Models. (a) Ex vivo fluorescence imaging: U251-MG glioblastoma tumors imaged using the IVIS system at different temperatures. The sequence shows tumors at physiological temperature (37 °C), in cryogenic conditions (−40 °C), and subsequently returned to 37 °C, with control tumors included for comparison. Dose administered: 20 mg/kg (200 μL). (b) Quantitative analysis of radiant efficiency: this graph depicts the total radiant efficiency of nTG-DFP-COF in the U251-MG tumors, illustrating significantly stronger emissions in the frozen state compared to physiological temperature. This enhancement underscores the probe’s effectiveness under cryosurgical conditions. The marked decrease in fluorescence upon rewarming the tumors to 37 °C highlights the temperature-responsive properties of nTG-DFP-COF, reinforcing its potential as a dynamic cryo-imaging tool. Significance levels indicated by asterisks: *p < 0.05, **p < 0.01, ***p < 0.001, ****p < 0.0001. (c) In Vivo Targeting and Biodistribution of nTG-DFP-COF. Sequential fluorescence images of mice bearing subcutaneous U251-MG glioblastoma tumors treated with nTG-DFP-COF, captured at 0, 24, and 48 h postinjection using the IVIS Spectrum imaging system. These images illustrate the dynamic accumulation and subsequent clearance of nTG-DFP-COF at the tumor site. (d) Post-mortem fluorescence imaging of U251-MG Glioblastoma tumors: sequential fluorescence images of a control mouse treated with PBS and imaged at physiological temperature (37 °C) 24 h post-treatment; a mouse treated with nTG-DFP-COF (20 mg/kg, 200 μL) under the same conditions showing initial fluorescence distribution in the tumor; and the mouse after cryo-treatment at 4 °C and −20 °C. These images highlight the temperature-dependent “turn-on” property of nTG-DFP-COF. All animals were sacrificed 24 h post-treatment, and fluorescence imaging was conducted using an IVIS Spectrum system, demonstrating the probe’s enhanced imaging contrast and specificity under targeted temperature conditions.

Fluorescence imaging used the IVIS Spectrum to quantitatively analyze the emitted fluorescence of nTG-DFP-COF before and after the freezing process. At 37 °C, both the control tumors and the tumors treated with nTG-DFP-COF showed weak emissions. However, tumors injected with nTG-DFP-COF and subsequently frozen exhibited significantly increased fluorescence (Figure 7a). The fluorescence intensity decreased significantly when the tumor temperature was brought back to the physiological level of 37 °C. We quantified the total radiant efficiency of nTG-DFP-COF in tumors. We observed a substantial increase in emission in the frozen state compared to physiological temperature, underscoring the enhanced radiance efficiency of nTG-DFP-COF under cryosurgical conditions (Figure 7b). After rewarming to physiological value, the fluorescence intensity decreased significantly. This distinct variance in fluorescence between physiological and frozen states suggests that nTG-DFP-COF are promising probes for real-time cryo-imaging. This ex vivo approach has preliminarily validated the efficacy of nTG-DFP-COF for cryo-imaging, demonstrating its capability to activate fluorescence at cold temperatures—a key property for visualizing cancer tissues under cryo-surgical conditions.

### Post-Mortem Tumor Targeting and Cryo-Imaging in Tumor-Bearing Mice

Athymic NU/J nude mice bearing subcutaneous U251-MG glioblastoma tumors (75–100 mm^3^) were used to evaluate the tumor-targeting efficacy of nTG-DFP-COF. Mice received a single intraperitoneal injection of nTG-DFP-COF at a dose of 20 mg/kg (200 μL volume). Tumor-specific fluorescence was monitored at 0, 24, and 48 h using the IVIS Spectrum imaging system. For comparison, a control group received intraperitoneal injections of PBS solution. Animals were euthanized at three time points—immediately after injection and 24 and 48 h postinjection—to assess the biodistribution of a probe within the tumors using post-mortem whole-mouse IVIS Spectrum imaging (Figure 7c). The results quantified the radiance efficiency of nTG-DFP-COF at physiological temperature (37 °C). No fluorescence emission was observed in the control tumors, while the tumors treated with nTG-DFP-COF showed a progressive increase in fluorescence signal that reached its maximum after 24 h of injection. This peak indicates efficient accumulation and retention of nTG-DFP-COF in the tumor, demonstrating its potential for targeted tumor therapy. After 48 h, a decrease in fluorescence intensity was observed, indicating that the probe was being cleared from the tumor site.

Additionally, targeted cryo-imaging was conducted on living mice with implanted subcutaneous U251-MG glioblastoma tumors (Figure 7d). Mice received intraperitoneal injections of nTG-DFP-COF at a dose of 20 mg/kg, 24 h before imaging to ensure localization of nanoparticles in the tumor cells. Post-mortem IVIS imaging at 37 °C showed significantly stronger fluorescence in tumors treated with nTG-DFP-COF compared to controls, demonstrating effective passive accumulation and targeted delivery of the probe. When the temperature was lowered to 4 and −20 °C, the fluorescence intensity in the treated tumors increased by 10% and 20%, respectively. This temperature-sensitive fluorescence enhancement significantly improves the visibility of tumors, potentially facilitating more precise and effective cryogenic treatment.

## Conclusion

In this study, we successfully developed a nanoscale Covalent Organic Framework (nTG-DFP-COF) optimized for real-time cryo-imaging. By liquid exfoliation, we have prepared water-dispersible nanosheets of nTG-DFP-COF that are biocompatible and can selectively accumulate in cancer cells without inducing cytotoxic effects. These nanosheets exhibit turn-on fluorescence at lower temperatures, which significantly improves their ability to delineate cancer tissue precisely in cryogenic conditions. The lower thermal energy at these temperatures reduces molecular vibrations and promotes the formation and stabilization of hydrogen bonds. This leads to a more rigid molecular structure that reduces nonradiative relaxation pathways like rotations and vibrations, thereby enhancing the likelihood of radiative decay processes. The increased molecular rigidity and the dominance of radiative relaxation contribute to a significant increase in fluorescence intensity, which greatly improves the visibility of tumors during cryogenic procedures.

The robust structural integrity and functional efficacy of nTG-DFP-COF, as well as its proven biosafety, provide a solid foundation for its potential clinical applications. This innovative material has been extensively tested for biocompatibility and has shown minimal toxicity and adverse reactions in biological settings, which supports its use in clinical settings. This characteristic is critical to the further development of nTG-DFP-COF as a safe tool that significantly improves the accuracy and safety of surgical procedures by enabling targeted visualization of cancerous tissue in real-time, thereby minimizing the risk to adjacent healthy tissue.

Encouraged by these promising results, future studies will involve live animal models in actual cryoablation procedures to comprehensively validate the efficacy of nTG-DFP-COF in enhancing surgical precision and safety. These studies will bridge the gap from preliminary findings to practical clinical applications, confirming the utility of nTG-DFP-COF in real-world oncology practice. Our selection of excitation and emission wavelengths (400–450 nm for excitation and 520 nm for emission) is tailored to surface tumor imaging, where lower penetration depths are beneficial and sufficient for precise imaging. This method improves resolution and contrast in superficial tissues and provides clearer images of surface-level structures. In future research, we plan to integrate NIR probes into nTG-DFP-COF to improve penetration depth and enable imaging of deeper tumors.

The breakthrough properties of nTG-DFP-COF represent a significant advance in surgical oncology, particularly in the cryogenic treatment of resistant cancers. This advance not only holds the potential to improve surgical outcomes but also paves the way for integrating diagnostic and therapeutic functions, including drug delivery, in a single platform. This makes nTG-DFP-COF a transformative cryo-imaging tool that could revolutionize oncologic surgery and improve the effectiveness of cancer treatments.

## Supporting Information Available

General materials and methods, synthesis, Figures S1–S26, references (1–13) (PDF)

## References

[ref1] Kwak K.; Yu B.; Lewandowski R. J.; Kim D. H. Recent progress in cryoablation cancer therapy and nanoparticles mediated cryoablation. Theranostics 2022, 12 (5), 2175–2204. 10.7150/thno.67530. 35265206 PMC8899563

[ref2] Chu K. F.; Dupuy D. E. Thermal ablation of tumours: biological mechanisms and advances in therapy. Nat. Rev. Cancer 2014, 14 (3), 199–208. 10.1038/nrc3672. 24561446

[ref3] Rodríguez S. A.; Arias Fúnez F.; Bueno Bravo C.; Rodríguez-Patrón Rodríguez R.; Sanz Mayayo E.; Palacios V. H.; Burgos Revilla F. J. Cryotherapy for primary treatment of prostate cancer: intermediate term results of a prospective study from a single institution. Prostate Cancer 2014, 2014, 571576 10.1155/2014/571576. 24693437 PMC3945790

[ref4] Caviezel A.; Terraz S.; Schmidlin F.; Becker C.; Iselin C. E. Percutaneous cryoablation of small kidney tumours under magnetic resonance imaging guidance: Medium-term follow-up. Scand. J. Urol. Nephrol. 2008, 42 (5), 412–416. 10.1080/00365590801951632. 18609277

[ref5] Baust J. G.; Bischof J. C.; Jiang-Hughes S.; Polascik T. J.; Rukstalis D. B.; Gage A. A.; Baust J. M. Re-purposing cryoablation: a combinatorial ‘therapy’ for the destruction of tissue. Prostate Cancer Prostatic Dis. 2015, 18 (2), 87–95. 10.1038/pcan.2014.54. 25622539

[ref6] Saliken J. C.; Donnelly B. J.; Rewcastle J. C. The evolution and state of modern technology for prostate cryosurgery. Urology 2002, 60 (2), 26–33. 10.1016/s0090-4295(02)01681-3. 12206845

[ref7] Mondal S. B.; Gao S.; Zhu N.; Liang R.; Gruev V.; Achilefu S. Real-time fluorescence image-guided oncologic surgery. Adv. Cancer Res. 2014, 124, 171–211. 10.1016/B978-0-12-411638-2.00005-7. 25287689 PMC4245053

[ref8] He Z.; Liu P.; Zhang S.; Yan J.; Wang M.; Cai Z.; Wang J.; Dong Y. A Freezing-Induced Turn-On Imaging Modality for Real-Time Monitoring of Cancer Cells in Cryosurgery. Angew. Chem., Int. Ed. 2019, 58 (12), 3834–3837. 10.1002/anie.201813239. 30600879

[ref9] Wan S.; Guo J.; Kim J.; Ihee H.; Jiang D. A Photoconductive Covalent Organic Framework: Self-Condensed Arene Cubes Composed of Eclipsed 2D Polypyrene Sheets for Photocurrent Generation. Angew. Chem., Int. Ed. 2009, 48 (30), 5439–5442. 10.1002/anie.200900881. 19434640

[ref10] Côté A. P.; Benin A. I.; Ockwig N. W.; O’Keeffe M.; Matzger A. J.; Yaghi O. M. Porous, Crystalline, Covalent Organic Frameworks. Science 2005, 310 (5751), 1166–1170. 10.1126/science.1120411. 16293756

[ref11] Li S.; Zou J.; Tan L.; Huang Z.; Liang P.; Meng X. Covalent organic frameworks: from linkages to biomedical applications. Chem. Eng. J. 2022, 446, 137148 10.1016/j.cej.2022.137148.

[ref12] Scicluna M. C.; Vella-Zarb L. Evolution of Nanocarrier Drug-Delivery Systems and Recent Advancements in Covalent Organic Framework–Drug Systems. ACS Appl. Nano Mater. 2020, 3 (4), 3097–3115. 10.1021/acsanm.9b02603.

[ref13] Zhou L.-L.; Guan Q.; Dong Y.-B. Covalent Organic Frameworks: Opportunities for Rational Materials Design in Cancer Therapy. Angew. Chem., Int. Ed. 2024, 63 (8), e202314763 10.1002/anie.202314763. 37983842

[ref14] Khan N.; Slathia G.; Kaliya K.; Saneja A. Recent progress in covalent organic frameworks for cancer therapy. Drug Discovery Today 2023, 28 (6), 103602 10.1016/j.drudis.2023.103602. 37119962

[ref15] Benyettou F.; Das G.; Nair A. R.; Prakasam T.; Shinde D. B.; Sharma S. K.; Whelan J.; Lalatonne Y.; Traboulsi H.; Pasricha R.; Abdullah O.; Jagannathan R.; Lai Z.; Motte L.; Gándara F.; Sadler K. C.; Trabolsi A. Covalent Organic Framework Embedded with Magnetic Nanoparticles for MRI and Chemo-Thermotherapy. J. Am. Chem. Soc. 2020, 142 (44), 18782–18794. 10.1021/jacs.0c05381. 33090806

[ref16] Benyettou F.; Kaddour N.; Prakasam T.; Das G.; Sharma S. K.; Thomas S. A.; Bekhti-Sari F.; Whelan J.; Alkhalifah M. A.; Khair M.; Traboulsi H.; Pasricha R.; Jagannathan R.; Mokhtari-Soulimane N.; Gándara F.; Trabolsi A. In vivo oral insulin delivery via covalent organic frameworks. Chem. Sci. 2021, 12 (17), 6037–6047. 10.1039/D0SC05328G. 33995999 PMC8098678

[ref17] Das G.; Benyettou F.; Sharama S. K.; Prakasam T.; Gándara F.; de la Peña-O’Shea V. A.; Saleh N. i.; Pasricha R.; Jagannathan R.; Olson M. A.; Trabolsi A. Covalent organic nanosheets for bioimaging. Chem. Sci. 2018, 9 (44), 8382–8387. 10.1039/C8SC02842G. 30542586 PMC6243473

[ref18] Bai L.; Phua S. Z. F.; Lim W. Q.; Jana A.; Luo Z.; Tham H. P.; Zhao L.; Gao Q.; Zhao Y. Nanoscale covalent organic frameworks as smart carriers for drug delivery. Chem. Commun. 2016, 52 (22), 4128–4131. 10.1039/C6CC00853D. 26877025

[ref19] Liu S.; Hu C.; Liu Y.; Zhao X.; Pang M.; Lin J. One-Pot Synthesis of DOX@Covalent Organic Framework with Enhanced Chemotherapeutic Efficacy. Chem.—Eur. J. 2019, 25 (17), 4315–4319. 10.1002/chem.201806242. 30735271

[ref20] Vyas V. S.; Vishwakarma M.; Moudrakovski I.; Haase F.; Savasci G.; Ochsenfeld C.; Spatz J. P.; Lotsch B. V. Exploiting Noncovalent Interactions in an Imine-Based Covalent Organic Framework for Quercetin Delivery. Adv. Mater. 2016, 28 (39), 8749–8754. 10.1002/adma.201603006. 27545588

[ref21] Liu S.; Yang J.; Guo R.; Deng L.; Dong A.; Zhang J. Facile Fabrication of Redox-Responsive Covalent Organic Framework Nanocarriers for Efficiently Loading and Delivering Doxorubicin. Macromol. Rapid Commun. 2020, 41 (4), 1900570 10.1002/marc.201900570. 31894599

[ref22] Liao C.; Liu S. Tuning the physicochemical properties of reticular covalent organic frameworks (COFs) for biomedical applications. J. Mater. Chem. B 2021, 9 (31), 6116–6128. 10.1039/D1TB01124C. 34278394

[ref23] Qian Y.; Li J.; Ji M.; Li J.; Ma D.; Liu A.; Zhao Y.; Yang C. Fluorescent Covalent Organic Frameworks: A Promising Material Platform for Explosive Sensing. Front. Chem. 2022, 10, 943813 10.3389/fchem.2022.943813. 35910724 PMC9334568

[ref24] Das G.; Ibrahim F. A.; Khalil Z. A.; Bazin P.; Chandra F.; AbdulHalim R. G.; Prakasam T.; Das A. K.; Sharma S. K.; Varghese S.; Kirmizialtin S.; Jagannathan R.; Saleh N. i.; Benyettou F.; Roz M. E.; Addicoat M.; Olson M. A.; Rao D. S. S.; Prasad S. K.; Trabolsi A. Ionic Covalent Organic Framework as a Dual Functional Sensor for Temperature and Humidity. Small 2024, 20, 2311064 10.1002/smll.202311064. 38396219

[ref25] Zhang X.; Xie X.; Wang H.; Zhang J.; Pan B.; Xie Y. Enhanced Photoresponsive Ultrathin Graphitic-Phase C3N4 Nanosheets for Bioimaging. J. Am. Chem. Soc. 2013, 135 (1), 18–21. 10.1021/ja308249k. 23244197

[ref26] Souza T. G. F.; Ciminelli V. S. T.; Mohallem N. D. S. A comparison of TEM and DLS methods to characterize size distribution of ceramic nanoparticles. J. Phys.: Conf. Ser. 2016, 733 (1), 012039 10.1088/1742-6596/733/1/012039.

[ref27] Filippov S. K.; Khusnutdinov R.; Murmiliuk A.; Inam W.; Zakharova L. Y.; Zhang H.; Khutoryanskiy V. V. Dynamic light scattering and transmission electron microscopy in drug delivery: a roadmap for correct characterization of nanoparticles and interpretation of results. Mater. Horiz. 2023, 10 (12), 5354–5370. 10.1039/D3MH00717K. 37814922

[ref28] Sun Z.; Ni Y.; Prakasam T.; Liu W.; Wu H.; Zhang Z.; Di H.; Baldridge K. K.; Trabolsi A.; Olson M. A. The Unusual Photochromic and Hydrochromic Switching Behavior of Cellulose-Embedded 1,8-Naphthalimide-Viologen Derivatives in the Solid-State. Chem.—Eur. J. 2021, 27 (36), 9360–9371. 10.1002/chem.202100601. 33831265

[ref29] Bannwarth C.; Ehlert S.; Grimme S. GFN2-xTB—An Accurate and Broadly Parametrized Self-Consistent Tight-Binding Quantum Chemical Method with Multipole Electrostatics and Density-Dependent Dispersion Contributions. J. Chem. Theory Comput. 2019, 15 (3), 1652–1671. 10.1021/acs.jctc.8b01176. 30741547

[ref30] Bannwarth C.; Caldeweyher E.; Ehlert S.; Hansen A.; Pracht P.; Seibert J.; Spicher S.; Grimme S. Extended tight-binding quantum chemistry methods. Wiley Interdiscip. Rev. Comput. Mol. Sci. 2021, 11 (2), e1493 10.1002/wcms.1493.

[ref31] Samanta S.; Raval P.; Manjunatha Reddy G. N.; Chaudhuri D. Cooperative Self-Assembly Driven by Multiple Noncovalent Interactions: Investigating Molecular Origin and Reassessing Characterization. ACS Cent. Sci. 2021, 7 (8), 1391–1399. 10.1021/acscentsci.1c00604. 34471682 PMC8393228

[ref32] Zydziak N.; Iqbal M. H.; Chaumont A.; Combes A.; Wasielewski E.; Legros M.; Jierry L.; Lavalle P.; Boulmedais F.; Chan-Seng D. Unexpected aqueous UCST behavior of a cationic comb polymer with pentaarginine side chains. Eur. Polym. J. 2020, 125, 109528 10.1016/j.eurpolymj.2020.109528.

[ref33] Scott J. N.; Nucci N. V.; Vanderkooi J. M. Changes in Water Structure Induced by the Guanidinium Cation and Implications for Protein Denaturation. J. Phys. Chem. A 2008, 112 (43), 10939–10948. 10.1021/jp8058239. 18839935 PMC2646201

[ref34] Benzine O.; Pan Z.; Calahoo C.; Bockowski M.; Smedskjaer M. M.; Schirmacher W.; Wondraczek L. Vibrational disorder and densification-induced homogenization of local elasticity in silicate glasses. Sci. Rep. 2021, 11 (1), 24454 10.1038/s41598-021-04045-6. 34961778 PMC8712522

[ref35] Monaco A.; Chumakov A. I.; Monaco G.; Crichton W. A.; Meyer A.; Comez L.; Fioretto D.; Korecki J.; Rüffer R. Effect of Densification on the Density of Vibrational States of Glasses. Phys. Rev. Lett. 2006, 97 (13), 135501 10.1103/PhysRevLett.97.135501. 17026042

[ref36] Pena R. B.; Deschamps T.; Le Floch S.; Berthelot A.; Romeo E.; Cunha T. R.; Peitl O.; Rodrigues A. D.; Martinet C.; Pizani P. S. Cold- and hot-densification of a depolymerized glass: A multiscale vibrational investigation of PbSiO3. J. Non-Cryst. Solids 2024, 646, 123246 10.1016/j.jnoncrysol.2024.123246.

[ref37] Bagheri S.; Masoodi H. R.; Abadi M. N. Estimation of individual NH···X (X = N, O) hydrogen bonding energies in some complexes involving multiple hydrogen bonds using NBO calculations. Theor. Chem. Acc. 2015, 134 (11), 127 10.1007/s00214-015-1738-z.

[ref38] Auerbach S. M.; Carrado K. A.; Dutta P. K. Handbook of Zeolite Science and Technology; CRC Press, 2003.

[ref39] Bhambri H.; Khullar S.; Sakshi; Mandal S. K. Nitrogen-rich covalent organic frameworks: a promising class of sensory materials. Mater. Adv. 2022, 3 (1), 19–124. 10.1039/D1MA00506E.

[ref40] Guo H.; Zhang L.; Xue R.; Ma B.; Yang W. Eyes of covalent organic frameworks: cooperation between analytical chemistry and COFs. Rev. Anal. Chem. 2019, 38( (1), ). 10.1515/revac-2017-0023.

[ref41] Gagnon K. J.; Perry H. P.; Clearfield A. Conventional and unconventional metal-organic frameworks based on phosphonate ligands: MOFs and UMOFs. Chem. Rev. 2012, 112 (2), 1034–1054. 10.1021/cr2002257. 22126609

[ref42] Gao Q.; Li X.; Ning G.-H.; Leng K.; Tian B.; Liu C.; Tang W.; Xu H.-S.; Loh K. P. Highly photoluminescent two-dimensional imine-based covalent organic frameworks for chemical sensing. Chem. Commun. 2018, 54 (19), 2349–2352. 10.1039/C7CC09866A. 29435533

[ref43] Sarkar P.; Sarkar T.; Singh H.; Sutariya B.; Ray S.; Das A.; Pramanik S. K.; Karan S. Microporous poly(triaminoguanidinium-amide) nanofilms with sub-nm precision for ultra-low molecular weight cut-off in nanofiltration. J. Mater. Chem. A 2023, 11 (26), 14390–14403. 10.1039/D3TA01842C.

[ref44] Haug W. K.; Moscarello E. M.; Wolfson E. R.; McGrier P. L. The luminescent and photophysical properties of covalent organic frameworks. Chem. Soc. Rev. 2020, 49 (3), 839–864. 10.1039/C9CS00807A. 31957763

[ref45] Kato T.; Yamada Y.; Nishikawa Y.; Otomo T.; Sato H.; Sato S. Origins of peaks of graphitic and pyrrolic nitrogen in N1s X-ray photoelectron spectra of carbon materials: quaternary nitrogen, tertiary amine, or secondary amine?. J. Mater. Sci. 2021, 56 (28), 15798–15811. 10.1007/s10853-021-06283-5.

[ref46] Stevens J. S.; Byard S. J.; Seaton C. C.; Sadiq G.; Davey R. J.; Schroeder S. L. M. Proton transfer and hydrogen bonding in the organic solid state: a combined XRD/XPS/ssNMR study of 17 organic acid–base complexes. Phys. Chem. Chem. Phys. 2014, 16 (3), 1150–1160. 10.1039/C3CP53907E. 24292812

[ref47] Isaacs M. A.; Davies-Jones J.; Davies P. R.; Guan S.; Lee R.; Morgan D. J.; Palgrave R. Advanced XPS characterization: XPS-based multi-technique analyses for comprehensive understanding of functional materials. Mater. Chem. Front. 2021, 5 (22), 7931–7963. 10.1039/D1QM00969A.

[ref48] Dobrovolskaia M. A.; Aggarwal P.; Hall J. B.; McNeil S. E. Preclinical Studies To Understand Nanoparticle Interaction with the Immune System and Its Potential Effects on Nanoparticle Biodistribution. Mol. Pharmaceutics 2008, 5 (4), 487–495. 10.1021/mp800032f. PMC2613572 18510338

[ref49] Dobrovolskaia M. A.; McNeil S. E. Understanding the correlation between in vitro and in vivo immunotoxicity tests for nanomedicines. J. Controlled Release 2013, 172 (2), 456–466. 10.1016/j.jconrel.2013.05.025. PMC5831149 23742883

[ref50] Morera D.; MacKenzie S. A. Is there a direct role for erythrocytes in the immune response?. Vet. Res. 2011, 42 (1), 89 10.1186/1297-9716-42-89. 21801407 PMC3199785

[ref51] Agashe H. B.; Dutta T.; Garg M.; Jain N. K. Investigations on the toxicological profile of functionalized fifth-generation poly (propylene imine) dendrimer. J. Pharm. Pharmacol. 2006, 58 (11), 1491–1498. 10.1211/jpp.58.11.0010. 17132212

[ref52] Dutta T.; Agashe H. B.; Garg M.; Balasubramanium P.; Kabra M.; Jain N. K. Poly (propyleneimine) dendrimer based nanocontainers for targeting of efavirenz to human monocytes/macrophages in vitro. J. Drug Targeting 2007, 15 (1), 89–98. 10.1080/10611860600965914. 17365278

[ref53] Malik N.; Wiwattanapatapee R.; Klopsch R.; Lorenz K.; Frey H.; Weener J. W.; Meijer E. W.; Paulus W.; Duncan R. Dendrimers: relationship between structure and biocompatibility in vitro, and preliminary studies on the biodistribution of 125I-labelled polyamidoamine dendrimers in vivo. J. Controlled Release 2000, 65 (1–2), 133–148. 10.1016/S0168-3659(99)00246-1. 10699277

[ref54] Bosi S.; Feruglio L.; Da Ros T.; Spalluto G.; Gregoretti B.; Terdoslavich M.; Decorti G.; Passamonti S.; Moro S.; Prato M. Hemolytic Effects of Water-Soluble Fullerene Derivatives. J. Med. Chem. 2004, 47 (27), 6711–6715. 10.1021/jm0497489. 15615520

[ref55] Davis M. E.; Chen Z. G.; Shin D. M. Nanoparticle therapeutics: an emerging treatment modality for cancer. Nat. Rev. Drug Discovery 2008, 7 (9), 771–782. 10.1038/nrd2614. 18758474

[ref56] Benyettou F.; Fahs H.; Elkharrag R.; Bilbeisi R. A.; Asma B.; Rezgui R.; Motte L.; Magzoub M.; Brandel J.; Olsen J. C.; Piano F.; Gunsalus K. C.; Platas-Iglesias C.; Trabolsi A. Selective growth inhibition of cancer cells with doxorubicin-loaded CB[7]-modified iron-oxide nanoparticles. RSC Adv. 2017, 7 (38), 23827–23834. 10.1039/C7RA02693E.

[ref57] Benyettou F.; Alhashimi M.; O’Connor M.; Pasricha R.; Brandel J.; Traboulsi H.; Mazher J.; Olsen J. C.; Trabolsi A. Sequential Delivery of Doxorubicin and Zoledronic Acid to Breast Cancer Cells by CB[7]-Modified Iron Oxide Nanoparticles. ACS Appl. Mater. Interfaces 2017, 9 (46), 40006–40016. 10.1021/acsami.7b11423. 29035507

[ref58] Hillaireau H.; Couvreur P. Nanocarriers’ entry into the cell: relevance to drug delivery. Cell. Mol. Life Sci. 2009, 66 (17), 2873–2896. 10.1007/s00018-009-0053-z. 19499185 PMC11115599

[ref59] Lojk J.; Bregar V. B.; Rajh M.; Miš K.; Kreft M. E.; Pirkmajer S.; Veranič P.; Pavlin M. Cell type-specific response to high intracellular loading of polyacrylic acid-coated magnetic nanoparticles. Int. J. Nanomed. 2015, 10, 1449–1462. 10.2147/ijn.s76134. PMC4340463 25733835

[ref60] Shen C.; Gu M.; Song C.; Miao L.; Hu L.; Liang D.; Zheng C. The tumorigenicity diversification in human embryonic kidney 293 cell line cultured in vitro. Biologicals 2008, 36 (4), 263–268. 10.1016/j.biologicals.2008.02.002. 18378163

[ref61] Osaka T.; Nakanishi T.; Shanmugam S.; Takahama S.; Zhang H. Effect of surface charge of magnetite nanoparticles on their internalization into breast cancer and umbilical vein endothelial cells. Colloids Surf., B 2009, 71 (2), 325–330. 10.1016/j.colsurfb.2009.03.004. 19361963

[ref62] Aiken N. R.; Gillies R. J. Phosphomonoester metabolism as a function of cell proliferative status and exogenous precursors. Anticancer Res. 1996, 16 (3B), 1393–1397. 8694507

[ref63] Hirata E.; Sahai E. Tumor Microenvironment and Differential Responses to Therapy. Cold Spring Harbor Perspect. Med. 2017, 7 (7), a026781 10.1101/cshperspect.a026781. PMC5495051 28213438

[ref64] de Visser K. E.; Joyce J. A. The evolving tumor microenvironment: From cancer initiation to metastatic outgrowth. Cancer Cell 2023, 41 (3), 374–403. 10.1016/j.ccell.2023.02.016. 36917948

[ref65] Prabhakar U.; Maeda H.; Jain R. K.; Sevick-Muraca E. M.; Zamboni W.; Farokhzad O. C.; Barry S. T.; Gabizon A.; Grodzinski P.; Blakey D. C. Challenges and key considerations of the enhanced permeability and retention effect for nanomedicine drug delivery in oncology. Cancer Res. 2013, 73 (8), 2412–2417. 10.1158/0008-5472.CAN-12-4561. 23423979 PMC3916009

[ref66] Phair R. D.; Misteli T. High mobility of proteins in the mammalian cell nucleus. Nature 2000, 404 (6778), 604–609. 10.1038/35007077. 10766243

[ref67] Zhitomirsky B.; Farber H.; Assaraf Y. G. LysoTracker and MitoTracker Red are transport substrates of P-glycoprotein: implications for anticancer drug design evading multidrug resistance. J. Cell. Mol. Med. 2018, 22 (4), 2131–2141. 10.1111/jcmm.13485. 29377455 PMC5867146

[ref68] Li X.; Li M.; Chen Y.; Qiao G.; Liu Q.; Zhou Z.; Liu W.; Wang Q. Chemical sensing failed by aggregation-caused quenching? A case study enables liquid/solid two-phase determination of N2H4. Chem. Eng. J. 2021, 415, 128975 10.1016/j.cej.2021.128975.

[ref69] Chen M.; Wen Q.; Gu F.; Gao J.; Zhang C. C.; Wang Q. Mussel chemistry assembly of a novel biosensing nanoplatform based on polydopamine fluorescent dot and its photophysical features. Chem. Eng. J. 2018, 342, 331–338. 10.1016/j.cej.2018.02.099.

[ref70] Leung C. W.; Hong Y.; Chen S.; Zhao E.; Lam J. W.; Tang B. Z. A photostable AIE luminogen for specific mitochondrial imaging and tracking. J. Am. Chem. Soc. 2013, 135 (1), 62–65. 10.1021/ja310324q. 23244346

[ref71] Ding D.; Li K.; Liu B.; Tang B. Z. Bioprobes Based on AIE Fluorogens. Acc. Chem. Res. 2013, 46 (11), 2441–2453. 10.1021/ar3003464. 23742638

[ref72] Mei J.; Leung N. L. C.; Kwok R. T. K.; Lam J. W. Y.; Tang B. Z. Aggregation-Induced Emission: Together We Shine, United We Soar. Chem. Rev. 2015, 115 (21), 11718–11940. 10.1021/acs.chemrev.5b00263. 26492387

[ref73] Michael P. L.; Lam Y. T.; Hung J.; Tan R. P.; Santos M.; Wise S. G. Comprehensive Evaluation of the Toxicity and Biosafety of Plasma Polymerized Nanoparticles. Nanomaterials 2021, 11 (5), 1176 10.3390/nano11051176. 33947114 PMC8145910

[ref74] Kumar M.; Kulkarni P.; Liu S.; Chemuturi N.; Shah D. K. Nanoparticle biodistribution coefficients: A quantitative approach for understanding the tissue distribution of nanoparticles. Adv. Drug Delivery Rev. 2023, 194, 114708 10.1016/j.addr.2023.114708. 36682420

[ref75] Song K. D. Percutaneous cryoablation for hepatocellular carcinoma. Clin. Mol. Hepatol. 2016, 22 (4), 509–515. 10.3350/cmh.2016.0079. 28081593 PMC5266346

