# Supplementary material for: Freezing-Activated Covalent Organic Frameworks for Precise Fluorescence Cryo-Imaging of Cancer Tissue
Source: J Am Chem Soc. 2025 Feb 27;147(10):8188–204. doi: 10.1021/jacs.4c13848 (PMC11912341; doi:10.1021/jacs.4c13848)
Supplement: Supplementary file 1 — ja4c13848_si_001.pdf [file ja4c13848_si_001.pdf]

Supporting Information

Freezing-Activated Covalent Organic Frameworks for Precise Fluorescence Cryo-Imaging of Cancer Tissue

Farah Benyettou,[1]‡* Gobinda Das,[1]‡ Maylis Boitet,[2] Sabu Varghese,[2] Mostafa Khair,[2] Akshaya Kumar Das,[1] Zineb Matouk,[3] Thirumurugan Prakasam,[1] Philippe Bazin,[4] Sudhir Kumar Sharma,[5] Sneha Thomas,[2] Yao He,[2] Rainer Straubinger,[2] Bikash Garai,[1] Ramesh Jagannathan,[5] Felipe Gándara,[6] Mohamad El-Roz,[4] Ali Trabolsi[1]*

[1] Chemistry Program, New York University Abu Dhabi (NYUAD), Abu Dhabi 129188, United Arab Emirates

[2] Core Technology Platforms, New York University Abu Dhabi (NYUAD), 129188 Abu Dhabi, United Arab Emirates

[3]. Technology Innovative Institute, Abu Dhabi, 9639, United Arab Emirates.

[4]. Normandie Univ, ENSICAEN, UNICAEN, CNRS, LCS, Caen, 14000, France.

[5] Engineering Program, New York University Abu Dhabi (NYUAD), Abu Dhabi 129188, United Arab Emirates

[6]. Instituto de Ciencia de Materiales de Madrid-CSIC, C. Sor Juana Inés de la Cruz 3, 28049 Madrid, Spain

‡ equal contribution

*Corresponding author. at105@nyu.edu, fb51@nyu.edu

Contents

1 - General Materials and Methods 4

2. Synthesis 5

2.1. Synthesis of the linkers 5

2.2. Synthesis of TG-DFP-COF 5

3. Characterizations 6

3.1. Fourier Transform Infrared (FTIR) Spectroscopy 6

3.2. High-Resolution Transmission Electron Microscopy (HRTEM). 7

3.3. Atomic Force Microscopy (AFM) 8

3.4. Stability of nTG-DFP-COF After Freezing Process 10

3.5. Solid State Temperature-Dependent Luminescent Study 13

3.6. Thermal Imaging 14

3.7. *In vitro* Fluorescence Imaging of nTG-DFP-COF 15

3.8. Molecular Dynamics Simulations 16

3.9. Solid-State NMR Spectroscopy 16

3.10. In-Situ -FTIR Spectroscopy 16

3.11. X-Ray Photoelectron (XPS) Spectroscopy 17

4. *In vitro* Biological Studies 18

4.1. Cell Culture 18

4.2. *In vitro* Biocompatibility Assessment 18

4.3. Hemolysis Assay 21

4.4. Intracellular Distribution Study Using TEM 22

4.5. *In vitro* Internalization Study by Fluorescence Microscopy 24

4.6. *In vitro* organelle Co-Localization Study by Confocal Microscopy 26

4.7. Bioimaging Experiments 30

5. *In vivo* Biological Studies 32

5.1. *In Vivo* Toxicity and Biocompatibility Assessment of nTG-DFP-COF in CD-1 Mice 32

5.2. Tumor Model Establishment 32

5.3. *Ex vivo* Cryo-imaging 33

5.4. Local Cryotherapy in Tumor-Bearing Mice 34

5.5. Post-mortem Biodistribution and Cryo-Imaging Study on Tumor-Bearing Mice 35

5.6. Statistical Analysis. 35

References 36

Table S1. Comparative Analysis of Fluorescent Probes for Cryo-Imaging. This table presents a detailed comparison of various fluorescent probes highlighting their test conditions, models used, temperature sensitivity, temperature range, biocompatibility, specificity, cytotoxicity, and additional features.

Fluorescent Probe	Conditions	Excitation/Emission (nm)	Temperature Sensitivity	Biocompatibility	Specificity	Additional Features	ref
**nTG-DFP-COF**	*In vitro and in vivo*: HeLa, U251-MG, HEK-293 cells<br><br>Nude mice with tumors	469/ 525	High sensitivity at low temperatures −40 to 35 °C	High	High specificity to cancer tissues	Enhanced fluorescence at low temperatures, water-dispersible	This work
**TABD-Py**	*In vitro* NIH 3T3 and HeLa cells	365/ 450	Upon freezing	High	High specificity to cancer cells during freezing	Aggregation-induced emission, interacts with ice crystals	1
**Cold-Responsive Polymeric Nanoparticles**	*In vivo* cancer immunotherapy female mice with breast tumors	-	Enhanced drug and gene release at subzero temperatures −20°C to RT	High	High specificity for cancer cells due to chitosan coating	Cold-triggered release of chemotherapy and immunotherapy agents	2
**Polymeric Nanoparticles (TPE-based)**	*In vitro* HeLa cells	405/ 440, 520 and 610	Moderate: 25-45 °C	Moderate no cytotoxicity at 40 μg/mL	Moderate	Full-color tunable emission, reversible stimuli response	3
**AIE-based Nano-Thermometer**	*In vitro* HUVEC cells	375/ 490	Low 24-38 °C	Moderate <10% cell viability reduction at ≤10 μg/mL	High	Fluorescence lifetime imaging, simple and low-cost fabrication	4
**Carbon Dots**	*In vitro* HeLa cells	400/ 550	Moderate 20-43 °C	High 85% after 48h of incubation at 100 mg mL^{-1}	Moderate	Fluorescence thermal imaging	5

1 - General Materials and Methods

All reagents and starting materials were purchased from Sigma-Aldrich and used without further purification. Deionized water was used from Millipore Gradient Milli-Q water purification system. Thin-layer chromatography (TLC) was performed on silica gel 60 F254 (E. Merck). The plates were inspected under UV light. Column chromatography was performed on silica gel 60F (Merck 9385, 0.040–0.063 mm). Infrared spectra were recorded on an Agilent Technologies Cary 600 Series FTIR Spectrometer using the ATR mode. The samples' PXRD patterns were recorded using an X-ray Panalytical Empyrean diffractometer. High-resolution transmission electron microscopy (HRTEM) images were obtained using a Talos F200X Scanning/Transmission Electron Microscope (STEM) with a lattice-fringe resolution of 0.14 nm at an accelerating voltage of 200 kV equipped with CETA 16M camera. The high-resolution images of periodic structures were analyzed using Velox software. The topography of the samples was analyzed by atomic force microscopy (JPK Bio AFM; Bruker Nano GmbH, Berlin, Germany). AFM scans were collected using JPK NanoWizard® software in Q1™ advance imaging mode. In this mode, optimal image setting was achieved by autocalibration of cantilever and sample properties. Silicon cantilevers (NanosensorsTM, Neuchatel, Switzerland) with resonant frequencies of 250–300 kHz and force constants of 100–130 Nm^{-1} were used. The set point value of 80 nN was used for imaging. AFM scans were collected at 512 points/lines with a scan speed of 50 µm/s at a fixed scan angle of 0°. Scan artifacts were minimized by acquiring a typical scan at an angle of 90° under identical image acquisition parameters. After imaging, the sample data was loaded to JPK Data processing software and further post-processed using Gwyddion™ free software (version 2.47), an SPM data visualization and analysis tool. Dynamic light scattering (DLS) measurements were performed on a Malvern Zetasizer NanoSeries to determine the Zeta(ζ)-potential as well as the hydrodynamic size of the particles. Each experiment was performed in triplicate. *In vitro*, *ex vivo*, and *post-mortem* fluorescence imaging was performed using the IVIS Spectrum (Revvity/Perkin Elmer, USA), and images were analyzed with Living Image software. The IVIS Spectrum is equipped with a cooled CCD camera (− 90°C) featuring a 2048 x 2048 sensor and a pixel size of 13.5 µm. Cells images were acquired using a Lionheart FX automated microscope.

2. Synthesis

2.1. Synthesis of the linkers

2,6-diformylpyridine (DFP) was synthesized according to published procedures with no modification.[6]

Triaminoguanidinium chloride (TGH.Cl) was synthesized according to published procedures with no modification.[7]

2.2. Synthesis of TG-DFP-COF

TG-DFP-COF was synthesized according to a published procedure[8] by mixing an aqueous solution (0.5 mL) of triaminoguanidinium chloride (TGH.Cl, 8.46 mg, 0.06 mmol) with a solution of 2,6-diformylpyridine (DFP, 12.15 mg, 0.09 mmol) in 1,4-dioxane (2 mL), which was gradually added. The resulting mixture was then stirred and subjected to microwave irradiation at 100 °C for 30 minutes (Figure S1). After heating, the mixture was allowed to cool to room temperature, the yellow-colored product, TG-DFP-COF, was collected via centrifugation and washed sequentially with 1,4-dioxane, ethanol, and water.

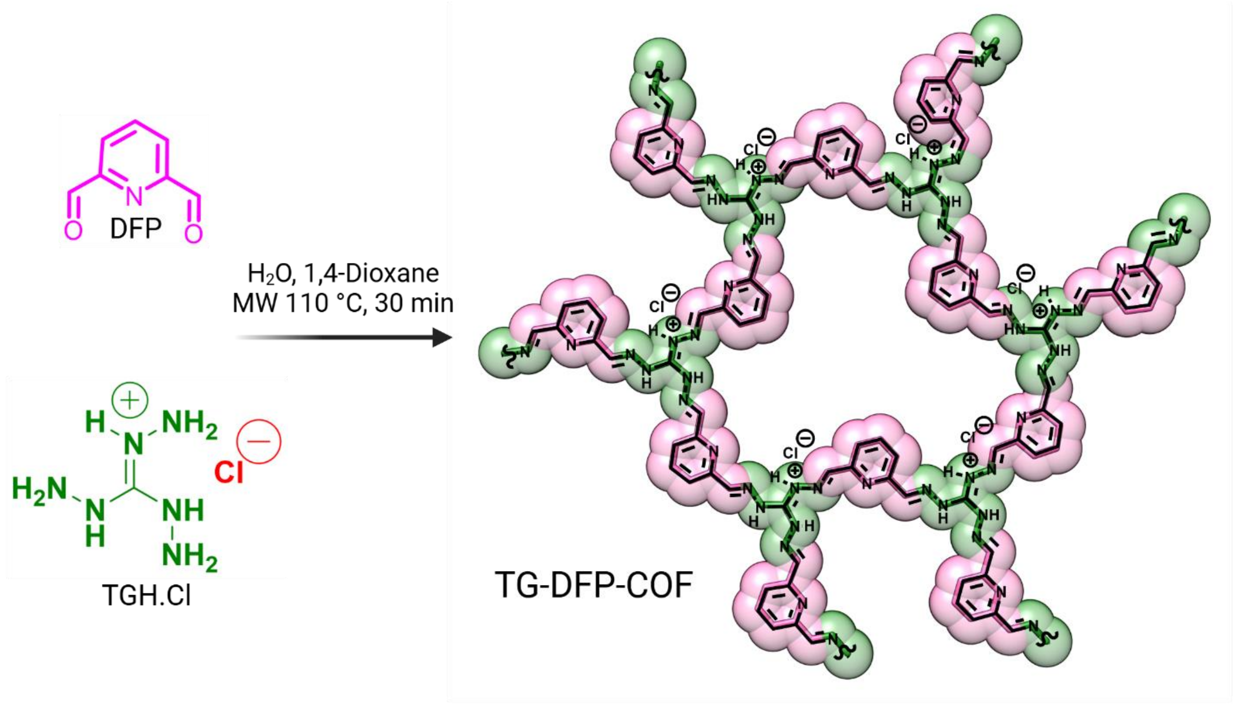

Figure S1. Microwave-assisted synthesis of TG-DFP-COF in bulk form using a mixture of 1,4-dioxane and water.

3. Characterizations

3.1. Fourier Transform Infrared (FTIR) Spectroscopy

In the FT-IR spectra of nTG-DFP-COF, the absence of the C=O stretching vibration band at 1723 cm^{-1} and the emergence of a new band at 1629 cm^{-1} confirm the formation of a C=N bond. Additionally, the disappearance of the N–H stretching vibration band at 3185 cm^{-1}, typically associated with the amino group in TGH, further substantiates the formation of imine bonds.

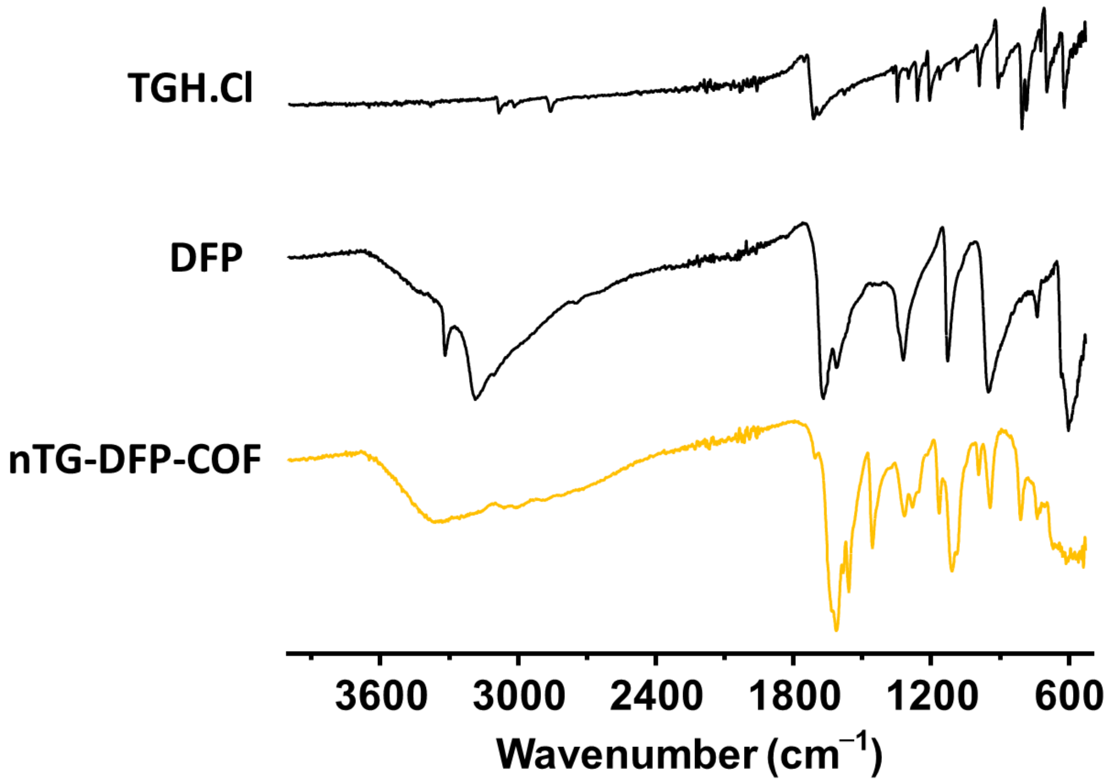

Figure S2. Stacked FTIR spectra of the nTG-DFP-COF (yellow), triamino guanidium hydrochloride salt (TGH.Cl, black), and 2,6-diformyl pyridine (DFP, black).

3.2. High-Resolution Transmission Electron Microscopy (HRTEM).

The samples were prepared on holey carbon film mounted on a copper grid. A drop of diluted particle solution was spotted on the grid and dried overnight at room temperature (298 K).

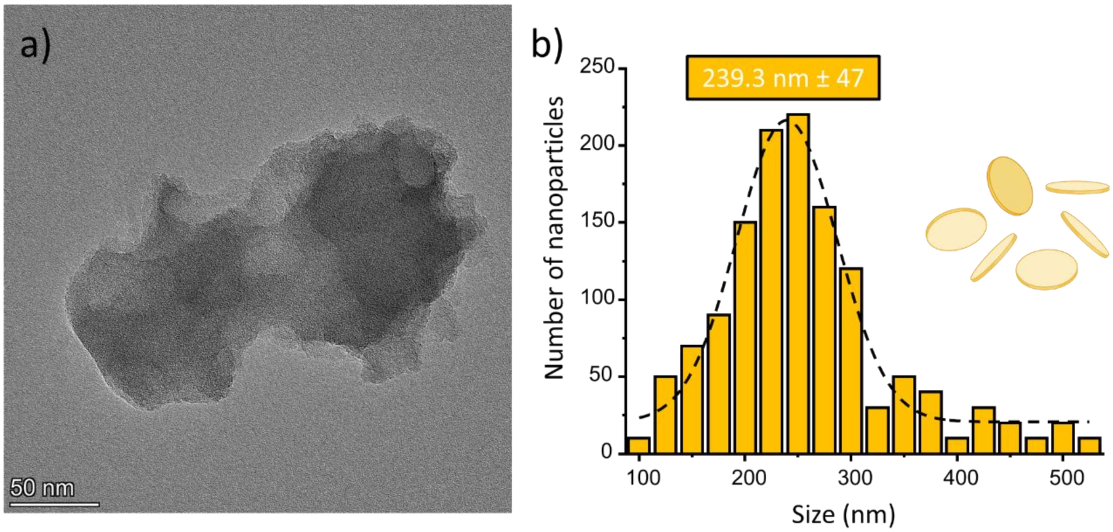

Figure S3. a) Transmission Electron Microscopy (TEM) image of nTG-DFP-COF. b) Particle size distribution of nTG-DFP-COF. The overlaid dashed lines represent Gaussian fit profiles, derived using the mean diameter calculated from the analysis of 500 individual particles.

3.3. Atomic Force Microscopy (AFM)

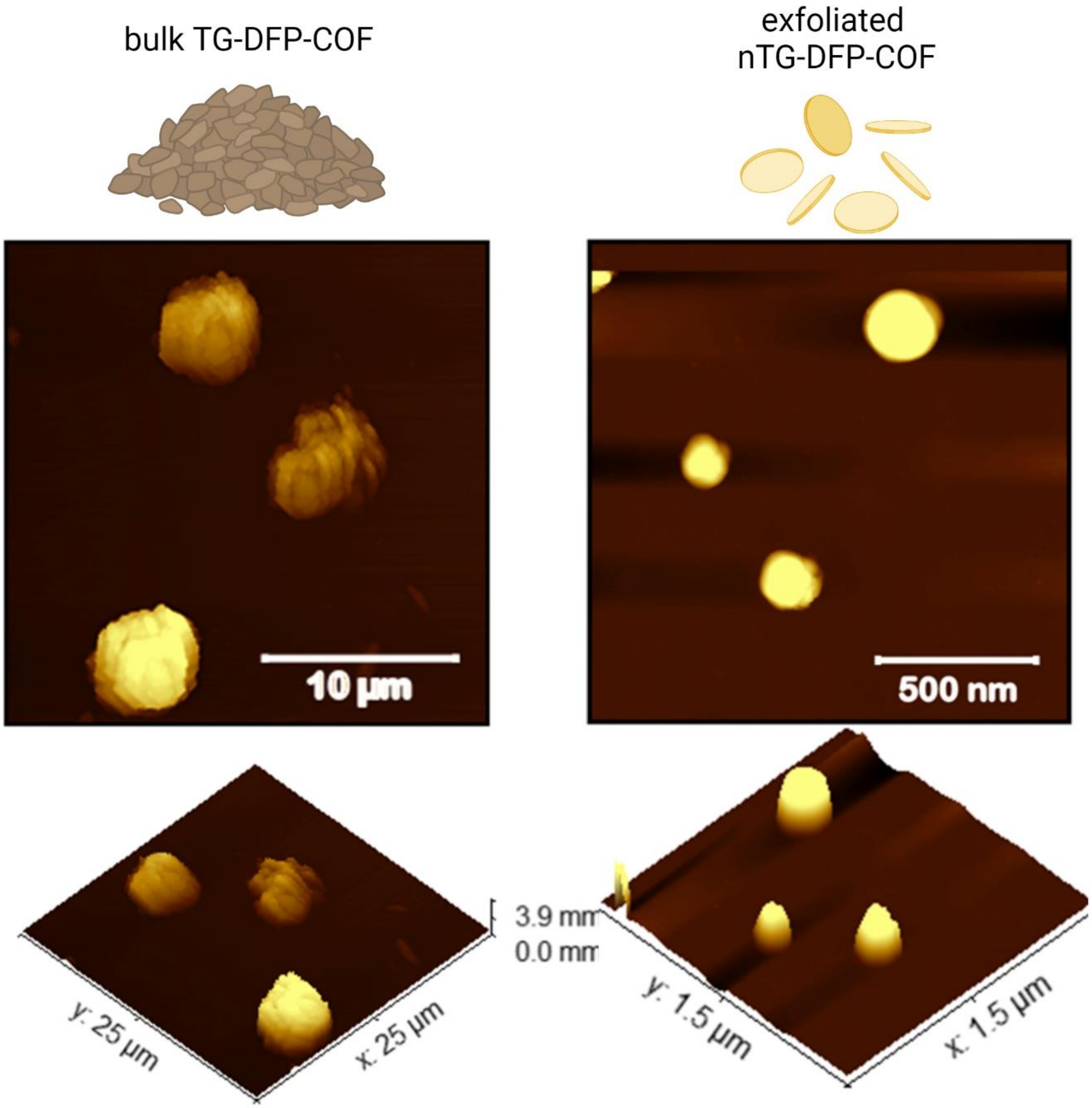

Figure S4. Atomic force microscopy (AFM) images displaying the topographical contrast between the bulk material (left) and the nTG-DFP-COF (right), with a distinct reduction in height and smoother surface profile.

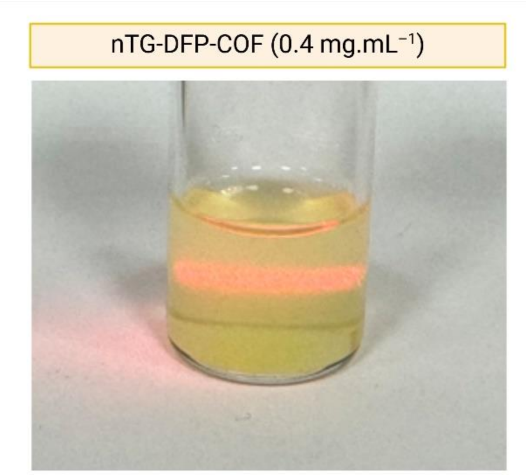

Figure S5. Tyndall Effect Observed in a Diluted nTG-DFP-COF Dispersion. The picture shows the Tyndall scattering effect in a translucent solution of nTG-DFP-COF (0.4 mg·mL^{-1}) when exposed to a laser beam, indicating the presence of highly monodisperse ultrathin nanosheets in water.

3.4. Stability of nTG-DFP-COF After Freezing Process

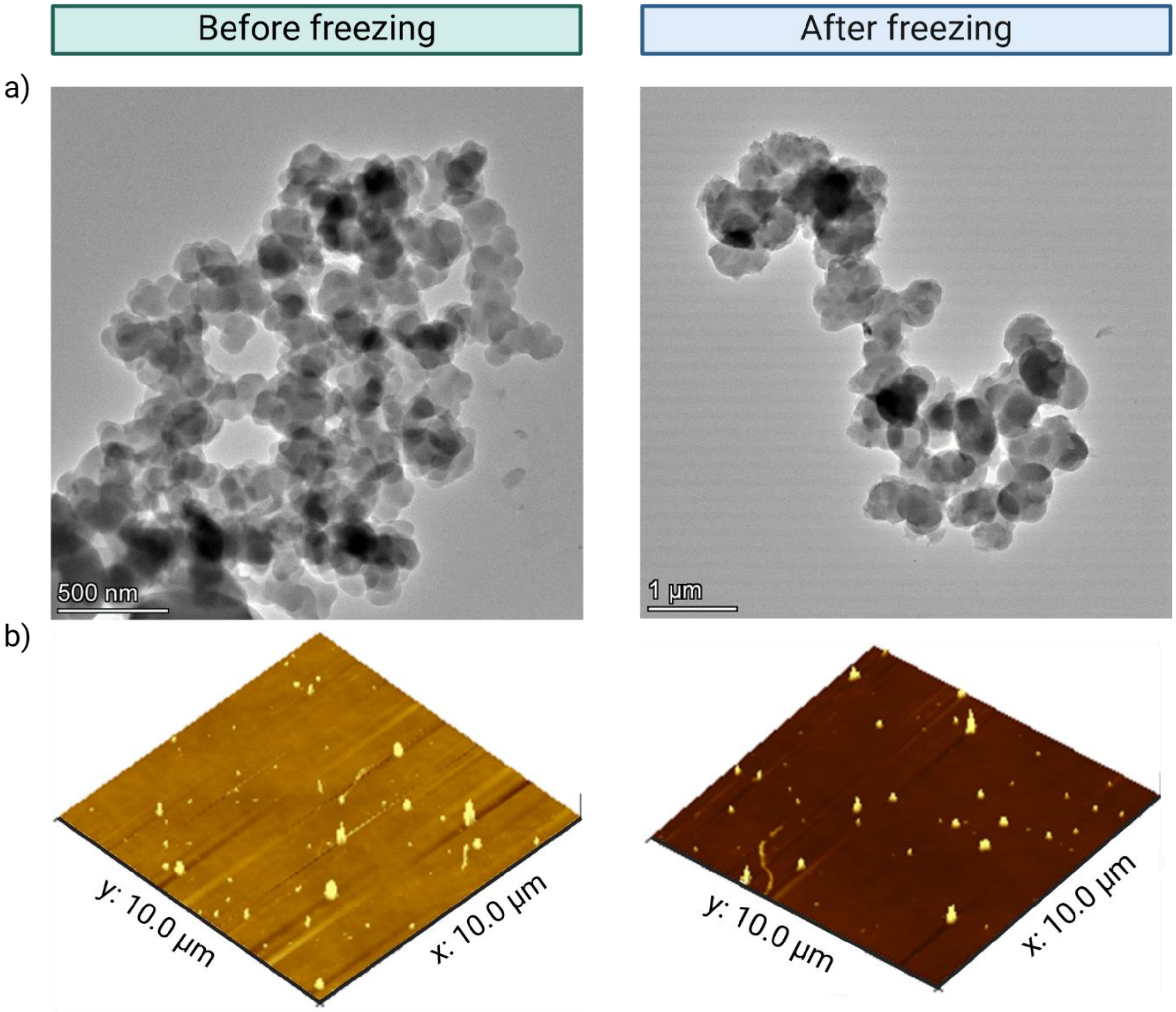

Figure S6. Comparative Analysis of nTG-DFP-COF Nanosheets Before and After Freezing. a) TEM images of nTG-DFP-COF nanosheets before (left) and after (right) undergoing a freezing process, demonstrating no significant changes in morphology or structural integrity. b) AFM images before (left) and after (right) freezing, further confirming the maintenance of surface morphology with no evident changes in the nanosheets' topography. These images collectively illustrate the robustness of nTG-DFP-COF nanosheets under cryogenic conditions, maintaining their structural characteristics after treatment.

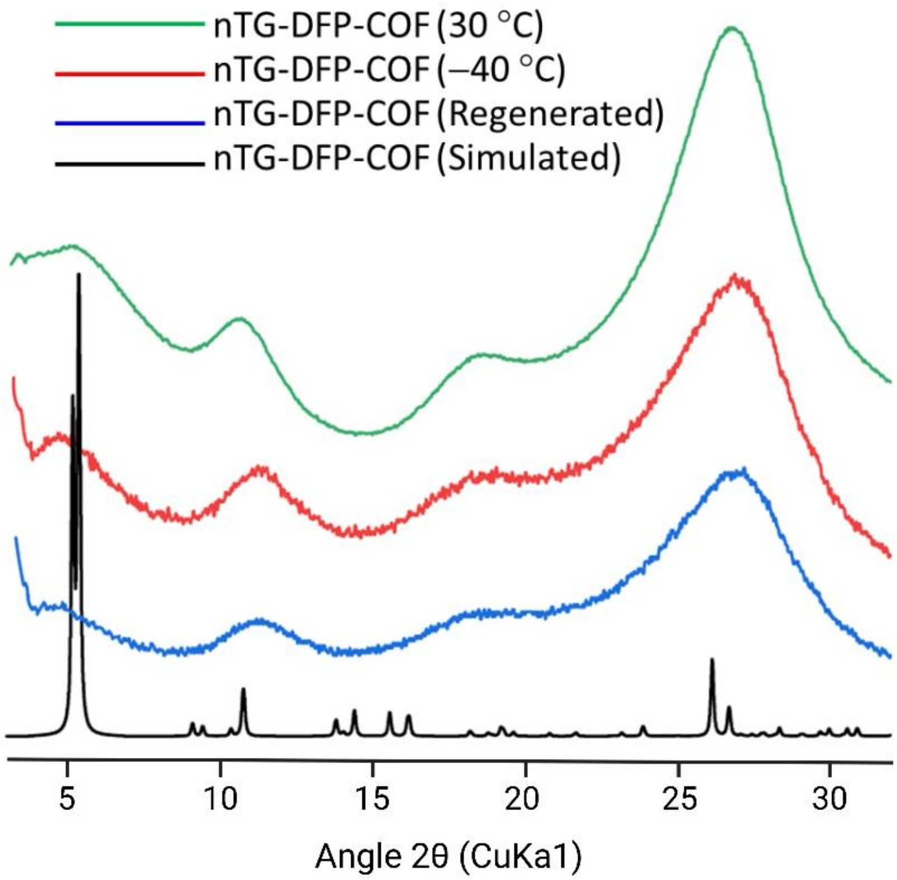

Figure S7. Variable Temperature Powder X-ray Diffraction (VT-PXRD) patterns of nTG-DFP-COF. The spectra depict the structural consistency of nTG-DFP-COF at various temperatures—room temperature (green line), −40 °C (red line), and after regeneration (blue line) as well as calculated (black line) PXRD patterns. The consistency across the spectra confirms the absence of structural changes in the nTG-DFP-COF, confirming its robustness and stability under varying thermal conditions.

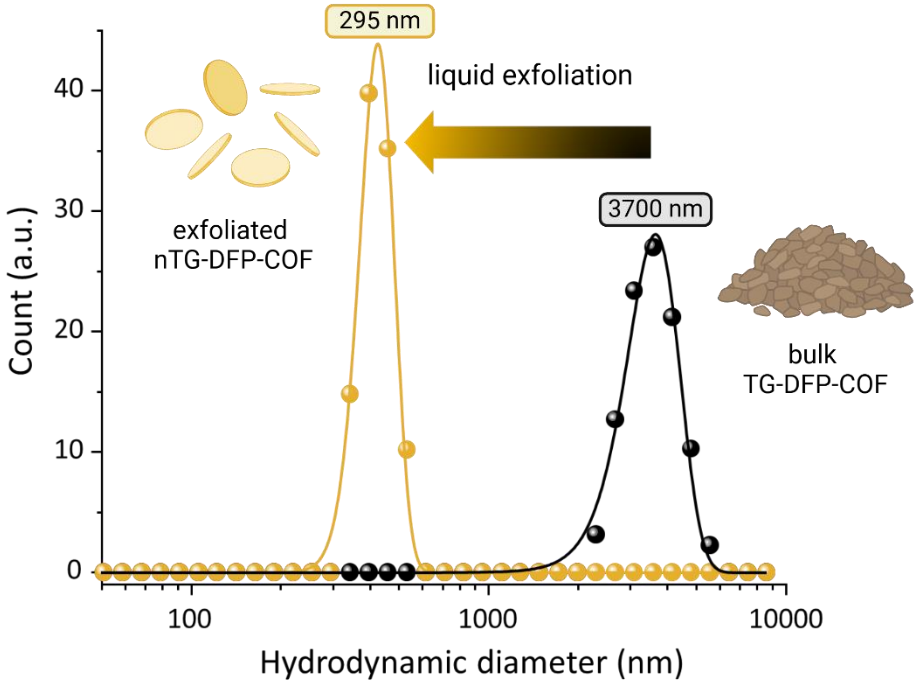

Figure S8. Stability and dispersibility assessments. Measurements of hydrodynamic diameter before and after liquid exfoliation of nTG-DFP-COF in aqueous solution.

3.5. Solid State Temperature-Dependent Luminescent Study

Solid state emission spectra were recorded using an FLS1000 spectrometer (Edinburgh, UK) equipped with an SC-10 as the sample holder with a front-face geometry, which also used a Xenon lamp to excite the sample at 320 and 375 nm with long pass filters (LPFs) at 375 (or 395 nm) and 420 nm, respectively, which were placed between the sample holder and the emission monochromator. A thermoelectrically cooled four-window cuvette holder was used together with an instrument software-powered controller to enable stable control of the sample temperatures, from 263 to 378 K.

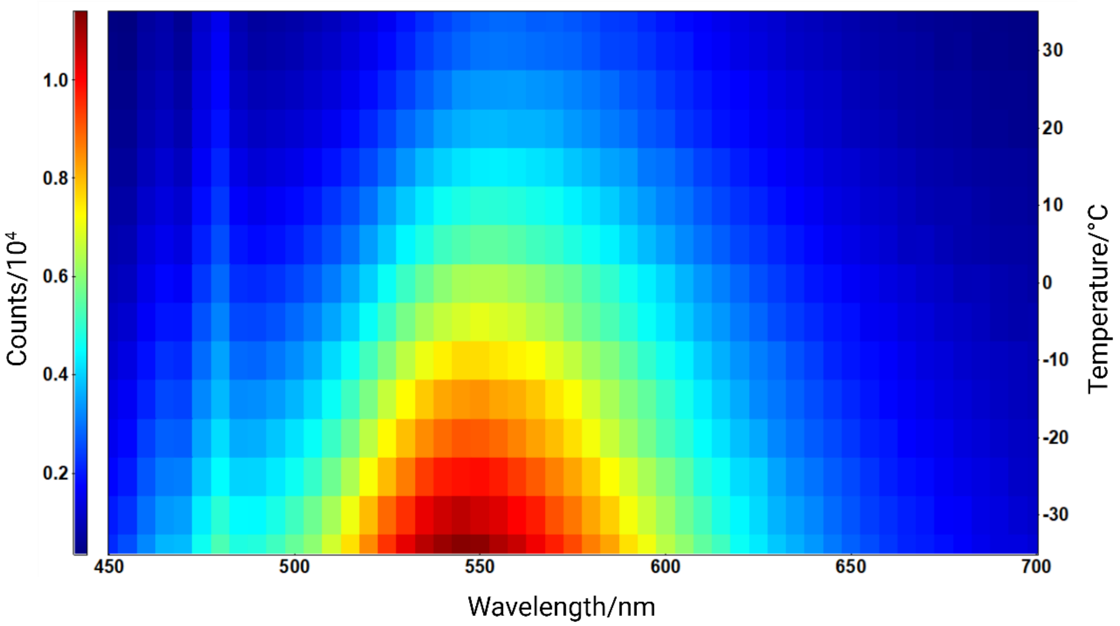

Figure S9. Temperature-dependent fluorescence heatmap of nTG-DFP-COF. The heatmap depicts the fluorescence intensity across a range of temperatures from −35°C to 35°C and wavelengths from 450 nm to 700 nm. This visualization clearly illustrates the thermally induced changes in luminescence, with a peak in intensity observed at lower temperatures (Λ_{ex} = 400 nm).

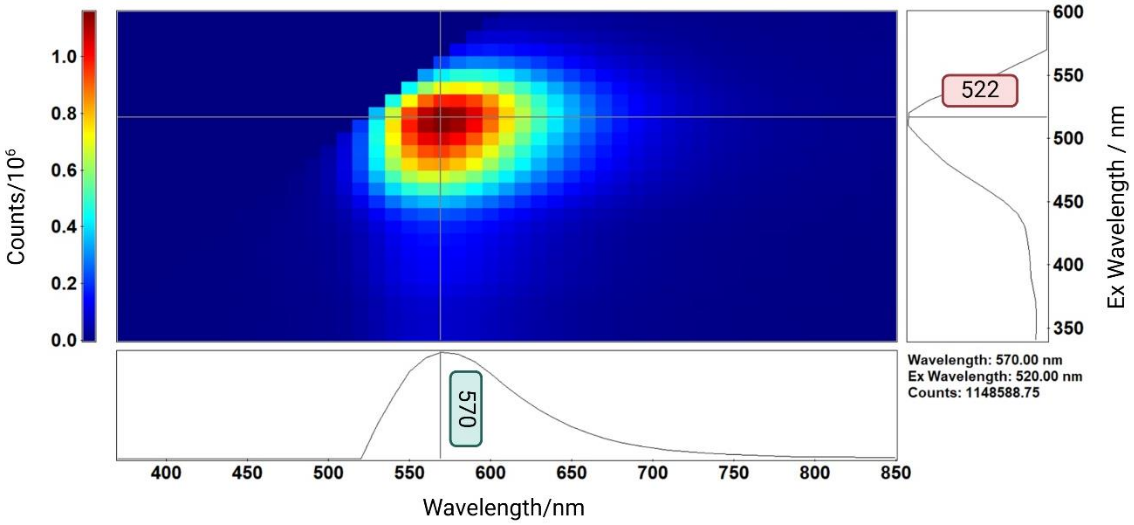

Figure S10. Excitation-Emission Matrix (EEM) of nTG-DFP-COF. This figure displays the EEM fluorescence landscape for nTG-DFP-COF, showcasing the excitation and emission wavelengths. The main fluorescence peak is observed at an excitation wavelength of 520 nm and an emission wavelength of 570 nm, as indicated by the reddest (most intense) area on the map. The color gradient from blue to red represents increasing fluorescence intensity, quantified on the left y-axis, with corresponding wavelength ranges detailed along the x and right y-axes.

3.6. Thermal Imaging

The thermal images were recorded using a FLIR E60bx thermal camera.

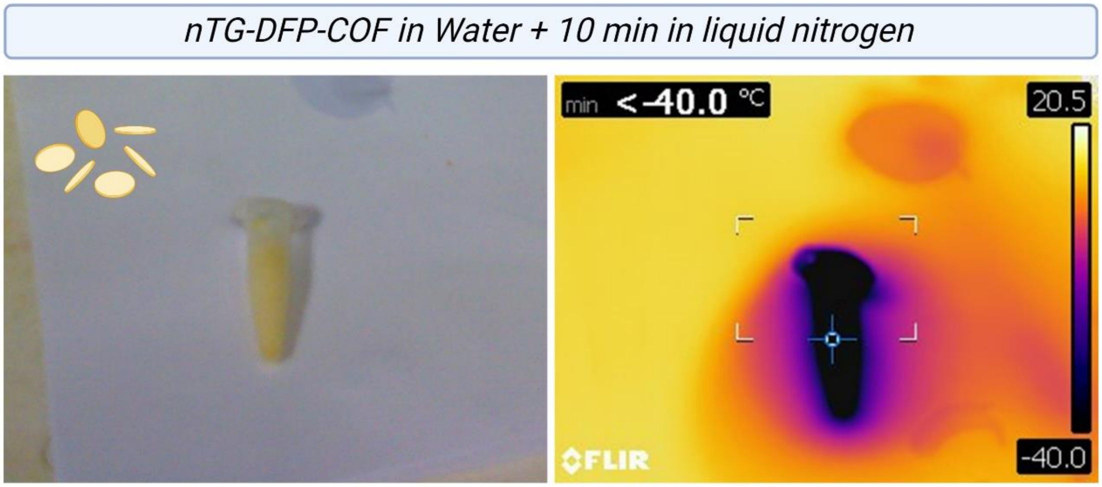

Figure S11. Visual and Thermal Analysis of nTG-DFP-COF in Water. This figure presents both a photographic and a thermal image of nTG-DFP-COF suspended in water at a concentration of 2 mg/mL. The sample was subjected to freezing by immersing the Eppendorf tube in liquid nitrogen

for 10 minutes. The thermal image captures and displays the temperature distribution across the sample immediately following its removal from the liquid nitrogen.

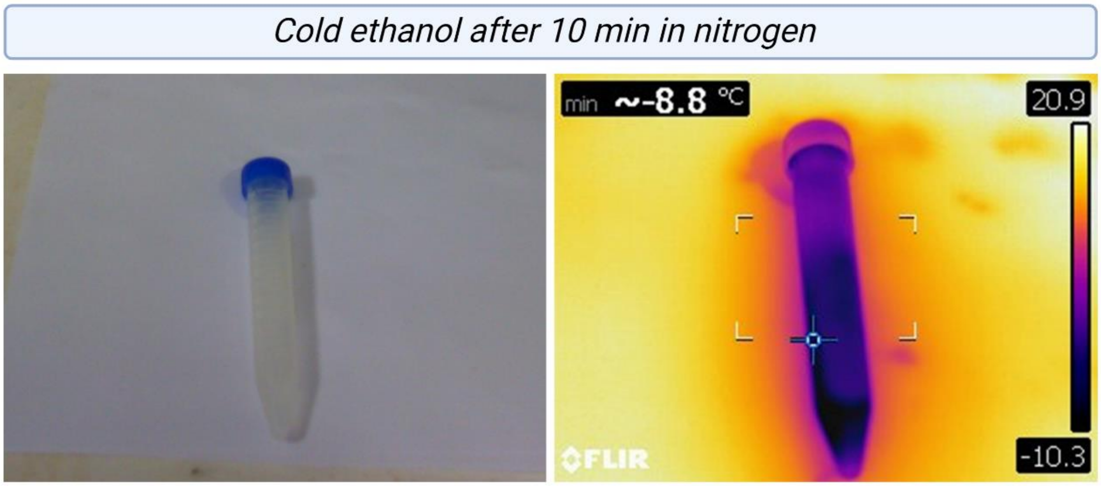

Figure S12. Visual and Thermal Analysis of Cold Ethanol. This figure includes both a photograph and a thermal image of 10 mL of ethanol after cooling. The sample was frozen by immersing the tube in liquid nitrogen for 10 minutes. The thermal image shows the temperature distribution of the ethanol immediately after being removed from the liquid nitrogen.

3.7. *In vitro* Fluorescence Imaging of nTG-DFP-COF

Fluorescence imaging of nTG-DFP-COF at varying concentrations (0.2 mg/mL to 1 mg/mL) was performed using the IVIS Spectrum system (Revvity, USA) with a 465 nm excitation filter and a 540 nm emission filter. The nTG-DFP-COF solutions were first warmed to 37 °C to simulate physiological conditions, then rapidly cooled to −40 °C using liquid nitrogen. Fluorescence images were analyzed by drawing regions of interest (ROIs) of consistent size within the Eppendorf tubes.

3.8. Molecular Dynamics Simulations

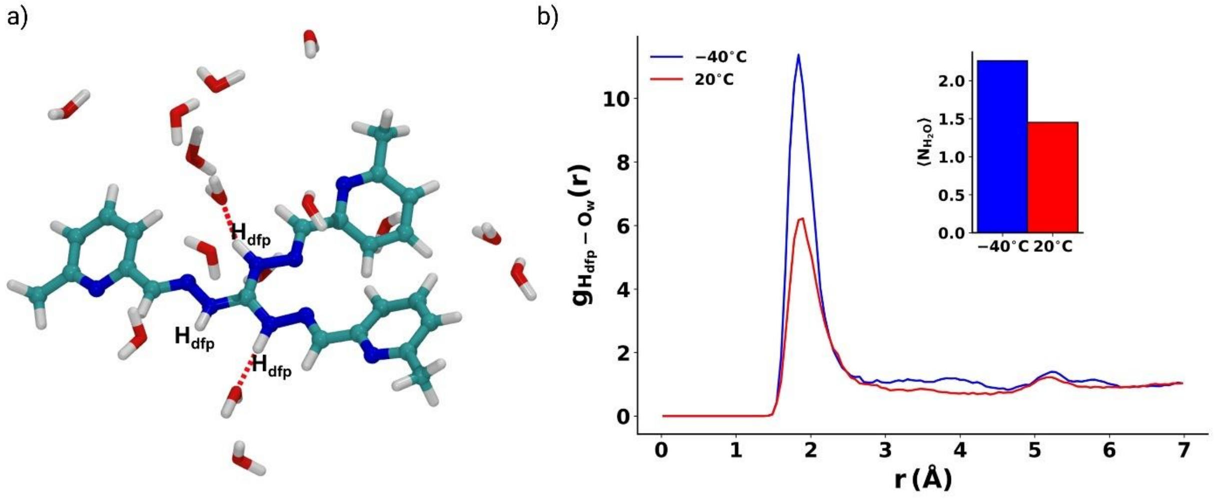

Figure S13. Molecular Dynamics Simulations Illustrating Temperature-Dependent Binding Affinity of Water Molecules with nTG-DFP-COF at −40 °C and 20 °C. a) The simulation system includes a fragment of nTG-DFP-COF along with 15 explicit water molecules to represent hydrogen bonding interactions between the nTG-DFP-COF and water molecules, which are depicted with dashed red lines. b) the graph represents the radial distribution function between the nTG-DFP-COF hydrogen atoms (labeled as H_{dfp} in panel a) and oxygen atoms of all water molecules at −40 °C (blue) and 20 °C (red). Inset: Average number of water molecules $<N_w>$ in the first solvation shell (3 Å from H_{dfp}) at −40 °C (blue) and 20 °C (red).

3.9. Solid-State NMR Spectroscopy

Magic Angle Spinning (MAS) solid-state NMR experiments were carried out on a Bruker Avance-HD 600 MHz spectrometer operating at a static field of 14.1 T using a 4.0 mm MAS probe. Powdered dry samples were packed into 4.0 mm zirconia rotors and were spun at a MAS frequency of 14 kHz. Cross-Polarization Magic Angle Spinning (CP/MAS) experiments were performed using a standard linearly ramped cross-polarization pulse sequence. ^{13}C chemical shifts were externally referenced to the adamantane CH_2 signal at 38.48 ppm on the TMS scale. NMR data were processed using TopSpin software.

3.10. In-Situ -FTIR Spectroscopy

The in-situ FTIR measurement was performant using a homemade in-situ FTIR reactor equipped with CaF_2 windows (PELICAEN cell). 1 wt.% of nTG-DFP-COF was diluted in KBr and prepared as a self-supported pellet (107 Pa/cm², S = 2 cm², m~ 100 mg). The pellet was activated at 40°C under vacuum overnight in order to eliminate the physisorbed water. Then, the measurement was

conducted under He to enhance thermal conductivity, and the temperature of the pellet was controlled using an external cryostat connected to the cell body. IR spectra were recorded with a Nicolet IS50 spectrophotometer (Thermo Fisher Scientific), equipped with a DTGS detector and an extended-KBr beam splitter in the region between 400 and 5500 cm^{-1}.

3.11. X-Ray Photoelectron (XPS) Spectroscopy

X-ray photoelectron spectroscopy (XPS) experiments were carried out on a Kratos Axis Ultra DLD spectrometer under a base pressure of ~ 2×10^{-10} mbar. A monochromated Al Kα X-ray source (1486.69 eV) irradiated samples at room temperature. XPS spectra were recorded from an analysis area of 700 µm × 300 µm. High-resolution XPS data of core levels were obtained with an energy resolution of 0.05 eV. For consistency, XPS measurements were calibrated to C1s (~ 285 eV). Data were analyzed using CasaXPS package with Shirley background subtraction.

Table S2. Binding energy (eV) and peak % for C 1s spectrum deconvolution curves of nTG-DFP-COF at room temperature and −40 °C (instrumental error is ±0.2eV).

	**nTG-DFP-COF**			
**C1s**	**Room Temperature**		**−40 °C**	
**Bond**	**Binding energy (eV)**	**%**	**Binding energy (eV)**	**%**
**C=C**	283.95	18.98 %	283.08	3.87 %
**C–N**	284.90	23.67 %	284.55	77.19 %
**N–C=N**	286.13	51.25 %	286.05	16.87 %
**C=O**	290.16	6.10 %	288.40	2.07 %

Table S3. Binding energy (eV) and peak % for N 1s spectrum deconvolution curves of nTG-DFP-COF at room temperature and −40 °C (instrumental error is ±0.2eV).

	**nTG-DFP-COF**			
**N1s**	**Room Temperature**		**−40 °C**	
**Bond**	**Binding energy (eV)**	**%**	**Binding energy (eV)**	**%**
**C=N Pyridine**	397.99	13.59 %		
**C=N Pyridine + C–N***			398.45	96.94 %
**C–N***	399.64	68.11 %	-	-
**C–NH–N***	401.4	10.8 %	402.01	3.06 %
**N-Oxidized**	401.18	7.5 %	-	-

4. *In vitro* Biological Studies

4.1. Cell Culture

Human malignant cervical carcinoma (HeLa, ATCC No. CCL-2), glioblastoma (U251-MG, ATCC No. 09063001) and non-cancer Human Embryonic Kidney 293 (HEK293; ATCC No. CRL-1573) cell lines were cultured in Dulbecco's Modified Eagle's medium (DMEM) supplemented with 10 % fetal bovine serum (FBS), 1 % penicillin/streptomycin and 20 mL L-glutamine at 5 % CO_2 and 37 °C.

4.2. *In vitro* Biocompatibility Assessment

4.2.1. Cell Viability

Cell viability was assessed using CellTiter-Blue® Cell Viability assay (CTB, Promega). The assay measures the metabolic reduction of a non-fluorescent compound, resazurin, into a fluorescent product, resofurin, in living cells. As non-viable cells rapidly lose their metabolic activity, the amount of the resofurin product can be used to estimate the number of viable cells following treatment. Once produced, resofurin is released from living cells into the surrounding medium. Thus, the fluorescence intensity of the medium is proportional to the number of viable cells present.

96-well plates were seeded with HEK-293, HeLa, and U251-MG (~5,000 cells per well in 100 µL of DMEM) and incubated at 37 °C for 24 hours. The medium was removed and replaced with fresh medium (control) or various concentrations of nTG-DFP-COF and incubated at 37 °C for 48 hours. After that, cells were incubated with 80 µL DMEM and 20 µL of CTB per well for 6 hours at 37 °C. The fluorescence of the resofurin product ($\lambda_{ex/em}$ 560/620) was measured. Untreated wells were used as control.

The percentage of cell viability was calculated using the following formula:

$$\textbf{Viability (\%)} = [(F_{treated} - F_{blank}) / (F_{control} - F_{blank})] \times 100$$

All assays were conducted in triplicate.

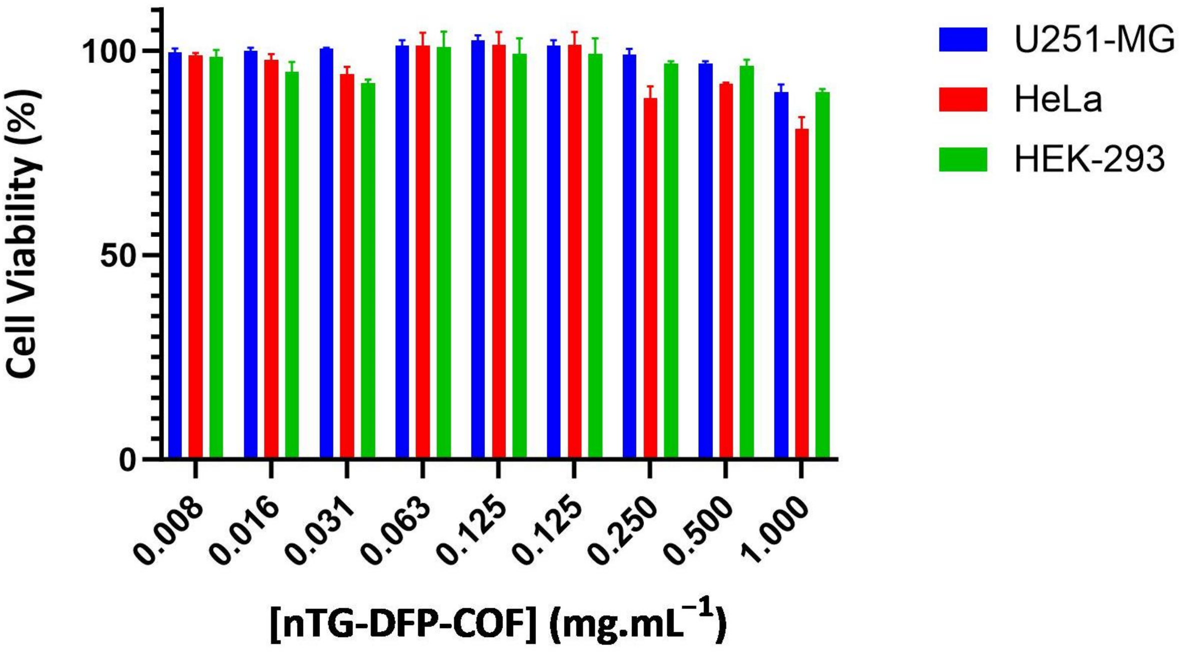

Figure S14. Cell viability of HEK-293 (green), HeLa (red), and U251-MG (blue) after 48-hour treatment with nTG-DFP-COF at concentrations up to 1 mg/mL. Error bars denote the standard deviations from triplicate measurements.

4.2.2. MTS assay

Cells were seeded at a density of 500 cells per well in 96-well tissue culture plates and incubated at 37°C in a humidified 5% CO_2 atmosphere to facilitate cell attachment. After 24 hours, cells were treated with either nTG-DFP-COF (10 µg/mL) or no additive (control). Following 24 hours of treatment, 20 µl of CellTiter 96® AQueous One Solution Reagent was added to each well, which already contained 100 µl of culture medium, and further incubated at 37°C for 1–4 hours in a humidified 5% CO_2 atmosphere. Cell viability was then assessed by measuring the absorbance at 490 nm using a Cytation 5 Microplate Reader (BioTek Instruments, Inc.). Statistical analyses were performed using GraphPad Prism software, with one-way ANOVA utilized to evaluate differences between treatment groups; significance was set at $p < 0.05$. All experiments were conducted in triplicate, and results were presented as mean ± standard deviation.

4.2.3. LDH Release Experiment

To assess the ability of nTG-DFP-COF to disrupt the plasma membrane of the cancer cells, the detection of lactate dehydrogenase (LDH) release from treated cells was performed using LDH-Glo™ Cytotoxicity Assay (Promega #J2380). Cells were incubated for 24 hours with no additives (control) or nTG-DFP-COF (10 µg/mL). After 24 hours of incubation, 5 µL of the medium was

diluted in 50 µL of LDH storage buffer and transferred into a new 96 well plates, and then, 50 µL of LDH detection reagent was added. Luminescence was recorded after 30 min of incubation using a Cytation 5 multimode reader (Biotek)

4.2.4. Cell Growth Analysis

Cells were seeded at a density of 500 cells per well in 96-well tissue culture plates and incubated at 37°C in a humidified 5% CO_2 atmosphere to facilitate cell attachment. After 24 hours, the cells were treated with either nTG-DFP-COF (10 µg/mL) or received no additive as a control. Subsequently, the plates were transferred to a Lionheart FX Automated Microscope (BioTek Instruments, Inc.) for live-cell imaging over a period of 72 hours. Images were captured every 4 hours using 4× brightfield settings to dynamically monitor cell growth.

Cell numbers in each well were quantified using Gen5 software (BioTek Instruments, Inc.) from the acquired images. Growth curves illustrating cell count over time were generated, and the cell growth dynamics were analyzed using the exponential Malthusian growth equation via GraphPad Prism software. All experiments were conducted in triplicate, and the results were reported as the mean ± standard error of the mean (SEM).

Table S4. Population Doubling Times (PDTs) for Control and nTG-DFP-COF Treated Cells. This table presents the population doubling times (PDTs) for HeLa, U251-MG, and HEK-293 cell lines over a three-day period. PDTs, calculated from the slope of the natural log of cell numbers plotted against time, indicate the time required for the cell population to double during the early log growth phase. The data compares PDTs between control groups and cells treated with 10 µg/mL of nTG-DFP-COF. As shown, the PDTs in treated groups are similar to those in control conditions, demonstrating that nTG-DFP-COF does not significantly affect cell proliferation.

PDTs	HeLa	U251-MG	HEK-293
Control	27.91 hours	26.54 hours	15.75 hours
nTG-DFP-COF	25.65 hours	20.94 hours	16.27 hours

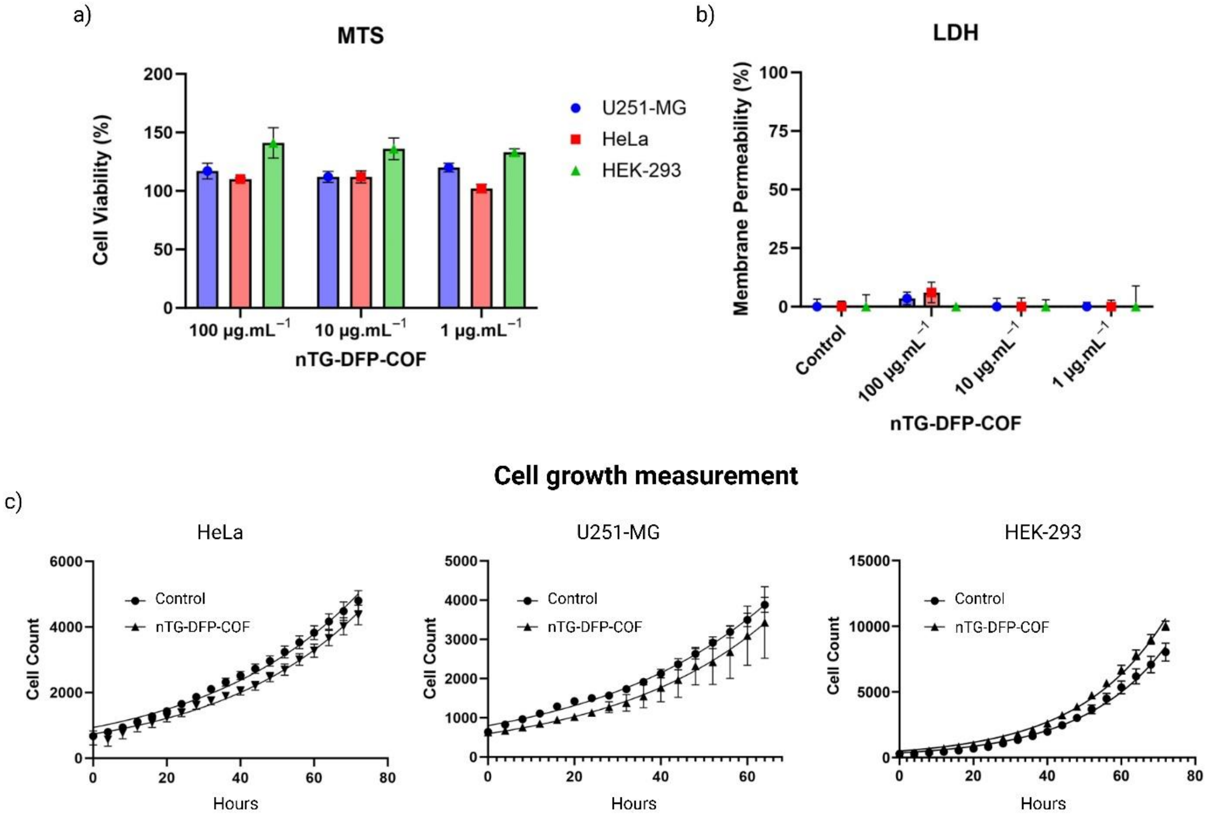

Figure S15. Evaluation of Cytotoxicity in HeLa, U251-MG, and HEK-293 Cell Lines via MTS, LDH, and Cell Growth Assays. a) Mitochondrial activity measured by the MTS assay across three concentrations (1, 10, and 100 µg/mL) of nTG-DFP-COF, highlighting cell viability in U251-MG (blue), HeLa (red), and HEK-293 (green) cell lines. b) Membrane integrity assessed through LDH leakage, indicating minimal perturbation across the same concentrations after 24 hours of exposure. c) Growth curves over 72 hours at 37 °C, showing cell count dynamics in control conditions and following incubation with 10 µg/mL of nTG-DFP-COF for each cell line. Data represent mean ± standard deviation (n=3).

4.3. Hemolysis Assay

When the external membrane of the erythrocytes is destroyed, hemoglobin is released.[9-11] It is possible to estimate the amount of destroyed erythrocytes in a given test by measuring the quantity of hemoglobin in a sample by spectrophotometry.[12]

Human blood was obtained from 3 healthy donors. 2.0 mL of an ethylenediaminetetraacetate-stabilized blood sample was added into 4 mL of physiological saline buffer (PBS), and then red blood cells were isolated by centrifugation (3000 rpm, 8 min). The red blood cells were washed five times with physiological saline and diluted into 2 % red blood cell suspensions.

Subsequently, nTG-DFP-COF (0.5, 1.0 and 2.0 mg.mL^{-1}) was added into the red blood cell suspensions at the predetermined concentration and mixed using a gentle vortex. Meanwhile, physiological saline with or without Triton X-100 (0.3 %) was added into the red blood cell suspensions as negative and positive controls, respectively. Samples were placed in a static condition at 37 °C for 1 h. Finally, all samples were centrifuged at 5000 rpm, and 100 µL of the supernatant was placed into a 96-well plate for detection at the wavelength of 540 nm. The hemolysis ratio (HR) represents the degree of red blood cell membranes destroyed in the samples.

$$\mathbf{HR\ (\%) = \frac{A_{sample} - A_{negative\ control}}{A_{positive\ control} - A_{negative\ control} \times 100}}$$

A_{sample}, $A_{positive\ control}$, and $A_{negative\ control}$ represented the absorbance of the sample, the positive control, and the negative control, respectively. These tests were performed in triplicate.

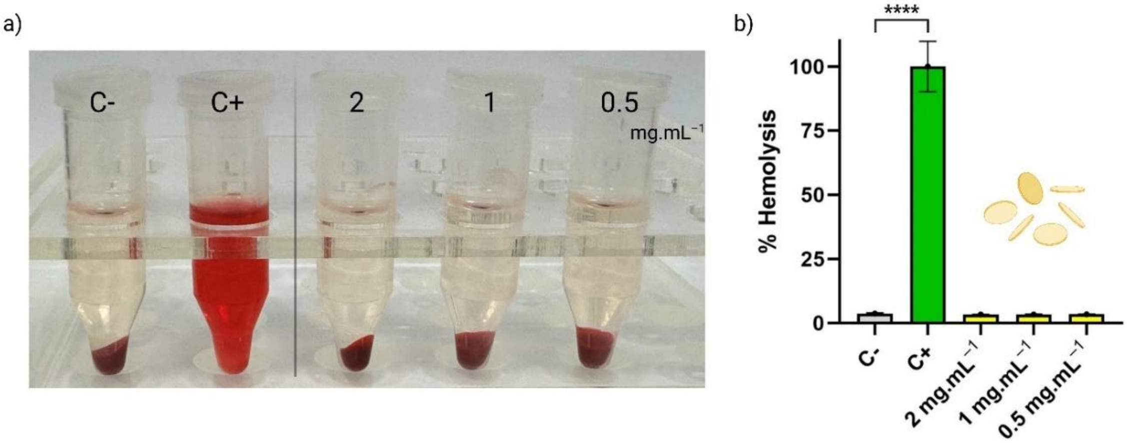

Figure S16. Hemolysis activity of nTG-DFP-COF. a) Photographs after centrifugation of fresh human blood incubated with different concentrations of nTG-DFP-COF up to 2 mg.mL^{-1} for 1 hour. b) Hemolysis rates (%) induced by different concentrations of nTG-DFP-COF up to 2 mg.mL^{-1}. Physiological saline in the absence or the presence of Triton X-100 (0.3 %) were respectively used as negative (C-) and positive (C+) controls. ****p< 0.0001, significantly different from negative control. Error bars represent standard deviations of triplicate measurements.

4.4. Intracellular Distribution Study Using TEM

For TEM analysis, HEK-293, HeLa, and U251-MG cells were seeded in T75 flasks in complete DMEM and incubated for 24 hours with cell-medium alone (control) or nTG-DFP-COF (10 µg/mL) in DMEM. After harvesting, cell pellets were washed twice with PBS. The cells were cryo-fixed within a few milliseconds at a pressure of 2000 bar under liquid nitrogen using a high-pressure freezer (Leica Microsystems, Germany). After freezing, the sample pod was released automatically into a liquid

nitrogen bath. While still in liquid nitrogen, the sample carrier was separated from the specimen pod using precooled fine-tipped tweezers and transferred to the cryo-transfer storage box for the flat specimen carrier, where the samples were stored in preparation for freeze substitution. Freeze substitution was performed using an automatic freeze substitution (AFS) unit (Leica EM AFS2, Heerbrugg, Switzerland) in a 10 mL solution of cold, dry absolute acetone (v/v) containing 1 % osmium tetroxide (w/v), 0.5 % uranyl acetate (w/v) and 5 % distilled water (v/v). The AFS unit was slowly warmed from −90 °C to 0 °C (2 °C/hour), with the temperature being held at both −60 °C and −30 °C for 8 hours. Samples were transferred to room temperature in a closed container to prevent condensation, rinsed with absolute acetone (3 × 5 minutes), and infiltrated with 30, 60, and 100 % Epon resin for 3 hours each. Epon was exchanged, and individual samples were embedded in 1 mL Eppendorf® lids for 24 hours at 60 ˚C. Finally, the samples were sectioned with an ultra-microtome at room temperature using a diamond knife, and the ultrathin sections were examined under TEM (Talos F200X STEM). The experiment was performed in triplicate.

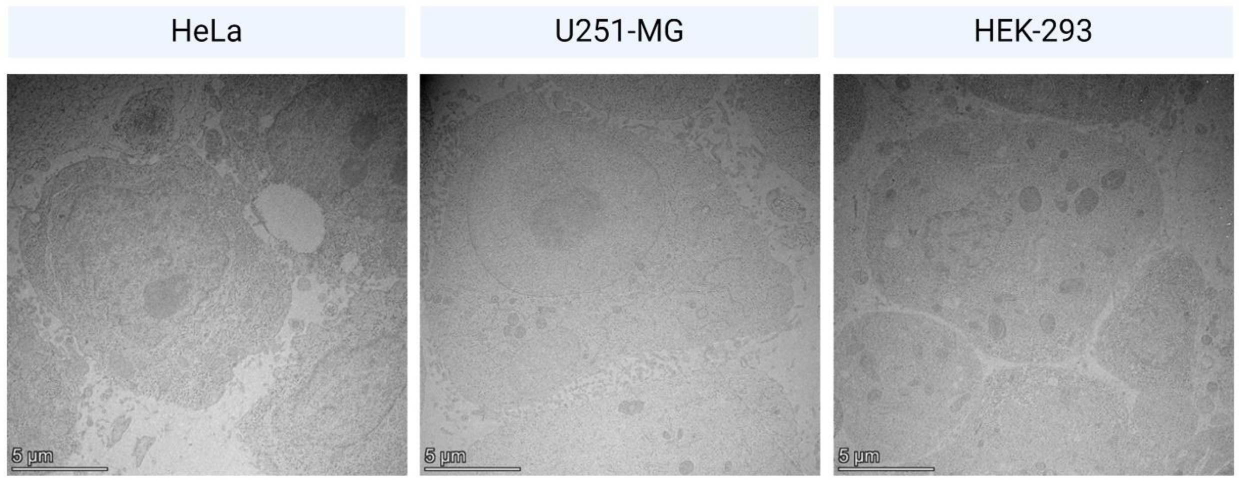

Figure S17. TEM images of HeLa, U251-MG, and HEK-293 control cells.

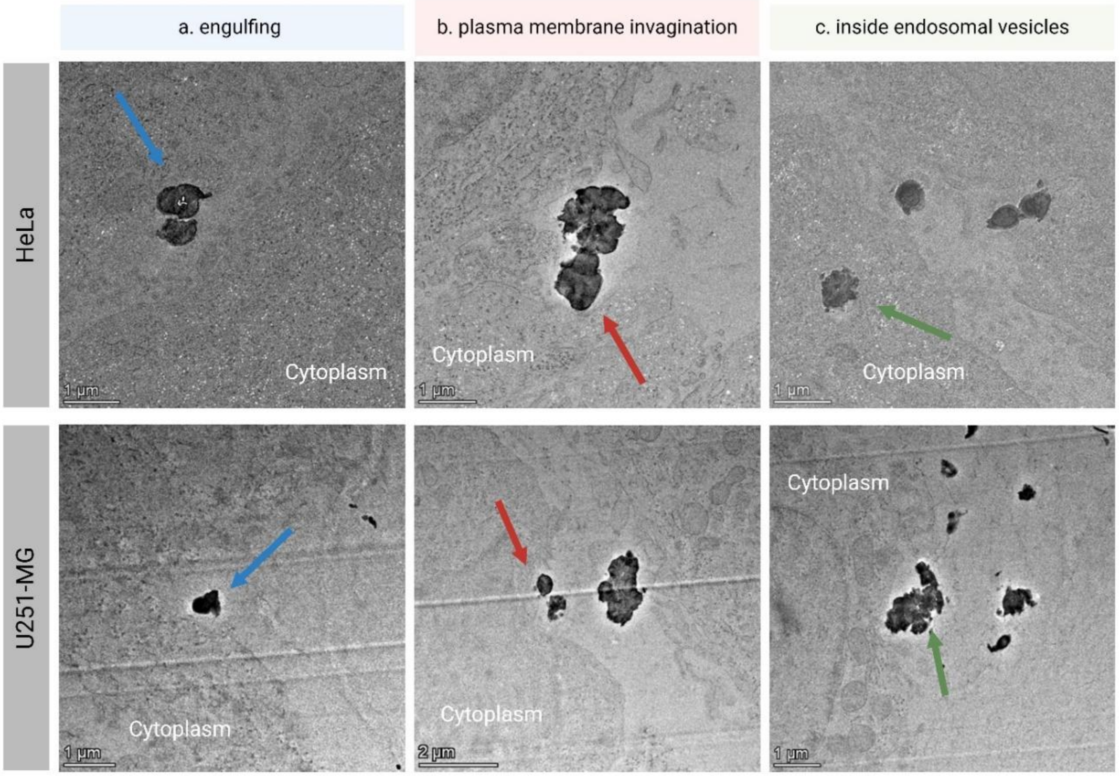

Figure S18. Intracellular Uptake of nTG-DFP-COF in Cancer Cells. Transmission electron microscopy (TEM) images show the interaction of nTG-DFP-COF with HeLa and U251-MG cells after 24 hours of incubation at a concentration of 10 µg/mL. a) Initial contact and engulfment of the nTG-DFP-COF nanosheets by the plasma membrane in both cell lines, indicated by the blue arrows. This interaction indicates the onset of membrane invagination. b) Progression of nTG-DFP-COF aggregates into deeper plasma membrane invaginations in both cell lines, shown by the red arrows, indicative of early endosomal formation. c) Localization of nTG-DFP-COF within endosomal vesicles inside the cytoplasm of both cell lines, indicated by green arrows. These stages illustrate the active internalization of nTG-DFP-COF in cancer cells.

4.5. *In vitro* Internalization Study by Fluorescence Microscopy

HeLa and U251-MG cells were seeded on sterile coverslips in complete DMEM and incubated for 24 hours to allow cell attachment. Cells were then treated either with no additives (control) or with nTG-DFP-COF (10 µg/mL) and incubated at temperatures of 4 °C and 37 °C for 4 hours. After treatment, the cells were washed with PBS, fixed with a 3.7% paraformaldehyde solution for 10 minutes, and then washed three times with PBS. The coverslips were mounted onto microscope slides using mounting medium. The samples were then analyzed using fluorescence microscopy. Each sample was analyzed in duplicate, and the entire experiment was repeated three times.

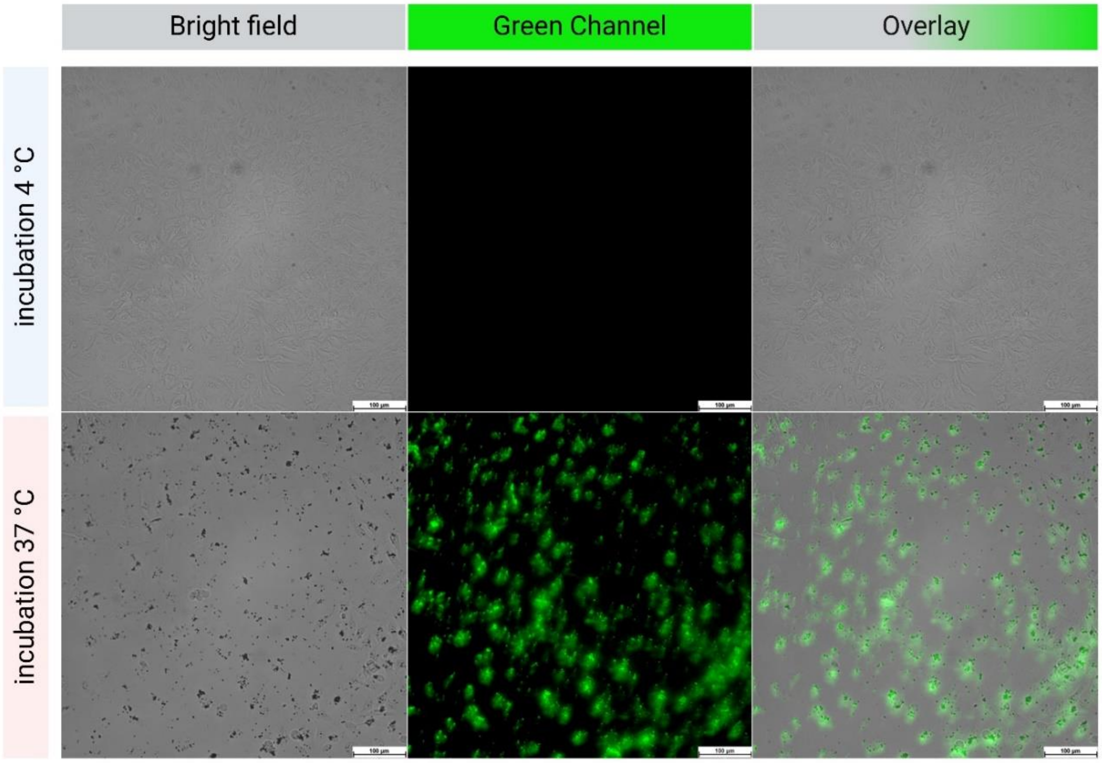

Figure S19. Fluorescence images of HeLa cells incubated for 4 hours with nTG-DFP-COF at 4 °C (top panel) and 37 °C (bottom panel) at a concentration of 10 µg/mL. The left column shows the bright field, the middle column shows the fluorescence in the green channel (λ_{ex} = 420 nm), and the right column is the overlay of both channels.

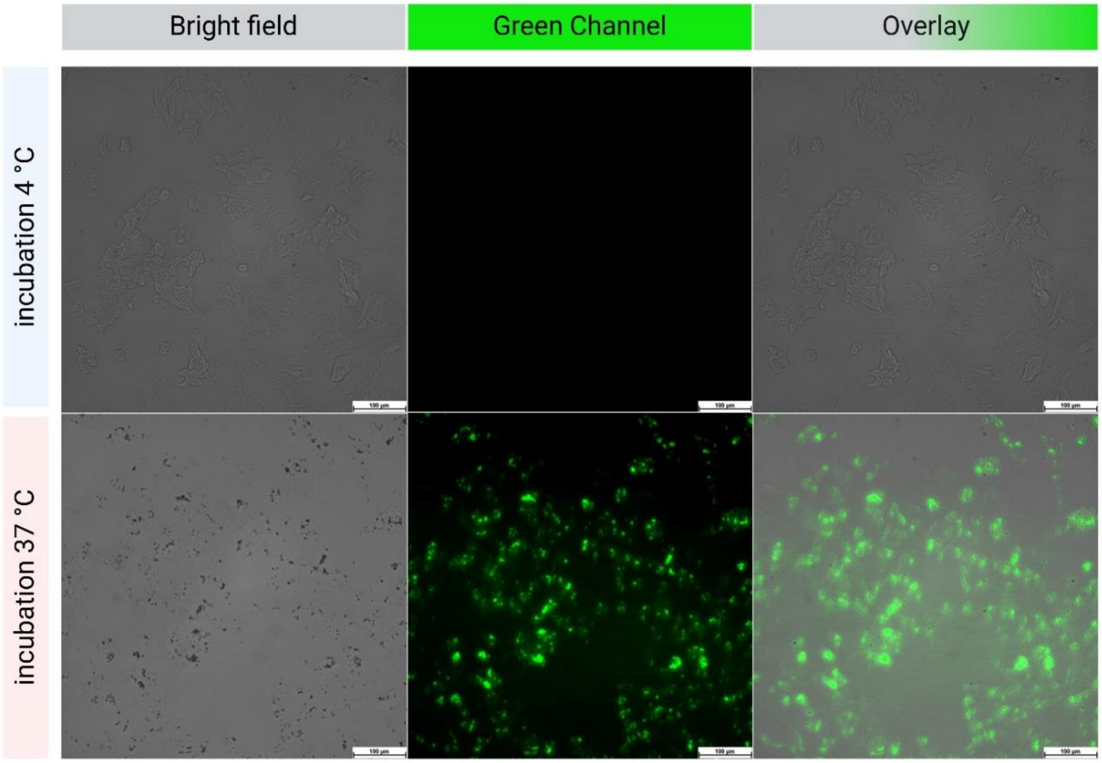

Figure S20. Fluorescence images of U251-MG cells incubated for 4 hours with nTG-DFP-COF at 4 °C (top panel) and 37 °C (bottom panel) at a concentration of 10 µg/mL. The left column shows the bright field, the middle column shows the fluorescence in the green channel (λ_{ex} = 420 nm), and the right column is the overlay of both channels.

4.6. *In vitro* Organelle Co-Localization Study by Confocal Microscopy

HeLa and U251-MG cells were seeded on sterile coverslips in complete DMEM and incubated for 24 hours. Cells were incubated for 4 hours without additives (control) or with nTG-DFP-COF (10 µg/mL). Cells were stained with organelle markers to understand the internalization of nTG-DFP-COF. Cells were incubated for 30 min with either LysoTracker™ 647 Deep Red (labeling lysosomes and endosomes), NucleusTracker™ Deep Red FM (labeling the nucleus), or CellMask™ 647 Deep Red (labeling the membrane), followed by three cycles of PBS washing. Then, for each experiment, the cells were fixed with formaldehyde solution (3.7 %) for 10 min, followed by washing thrice with PBS. The cells were kept for 5 min in PBS during washing cycles. The coverslips were then fixed onto a microscope slide.

The intracellular internalization of nTG-DFP-COF was observed by confocal microscopy (Olympus FV1000MPE), measuring the fluorescence signal of nTG-DFP-COF (λ_{ex} = 410 nm) in the cells and

the fluorescence emission of the 3 organelle markers labeling the plasmic membrane, lysosomes, and mitochondria (λ_{ex} = 561 nm).

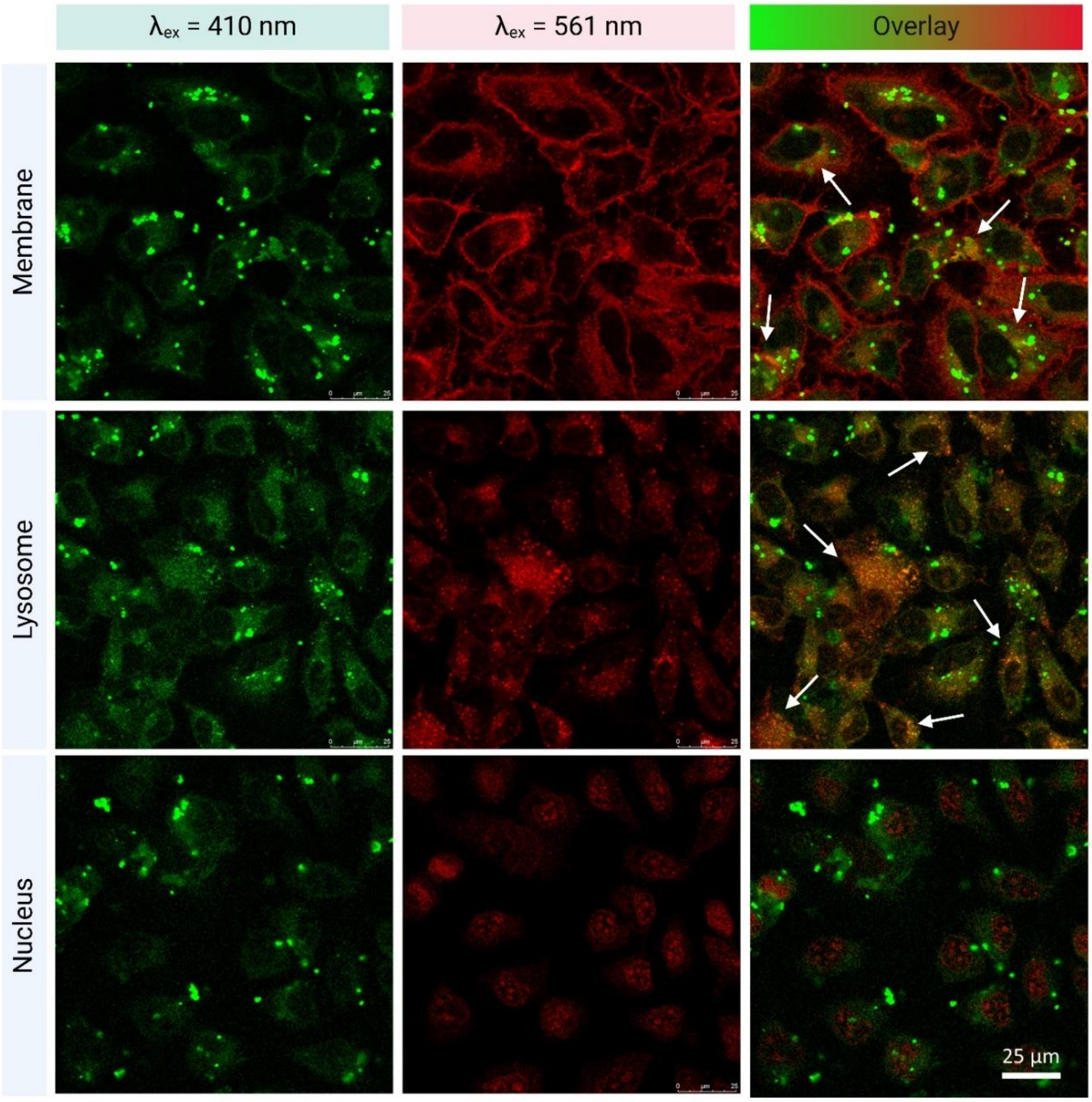

Figure S21. Confocal Laser Scanning Microscopy (CLSM) Images of HeLa Cells Following a 4-hour Treatment with nTG-DFP-COF. Cells were treated with nTG-DFP-COF at a concentration of 10 µg/mL and co-stained using red fluorescent markers to label the plasma membrane, lysosomes, and nucleus. Imaging channels include nTG-DFP-COF (λ_{ex} = 410 nm) and red markers (λ_{ex} = 561 nm). This figure illustrates the subcellular localization and the possible intracellular pathways of nTG-DFP-COF. White arrows show co-localization.

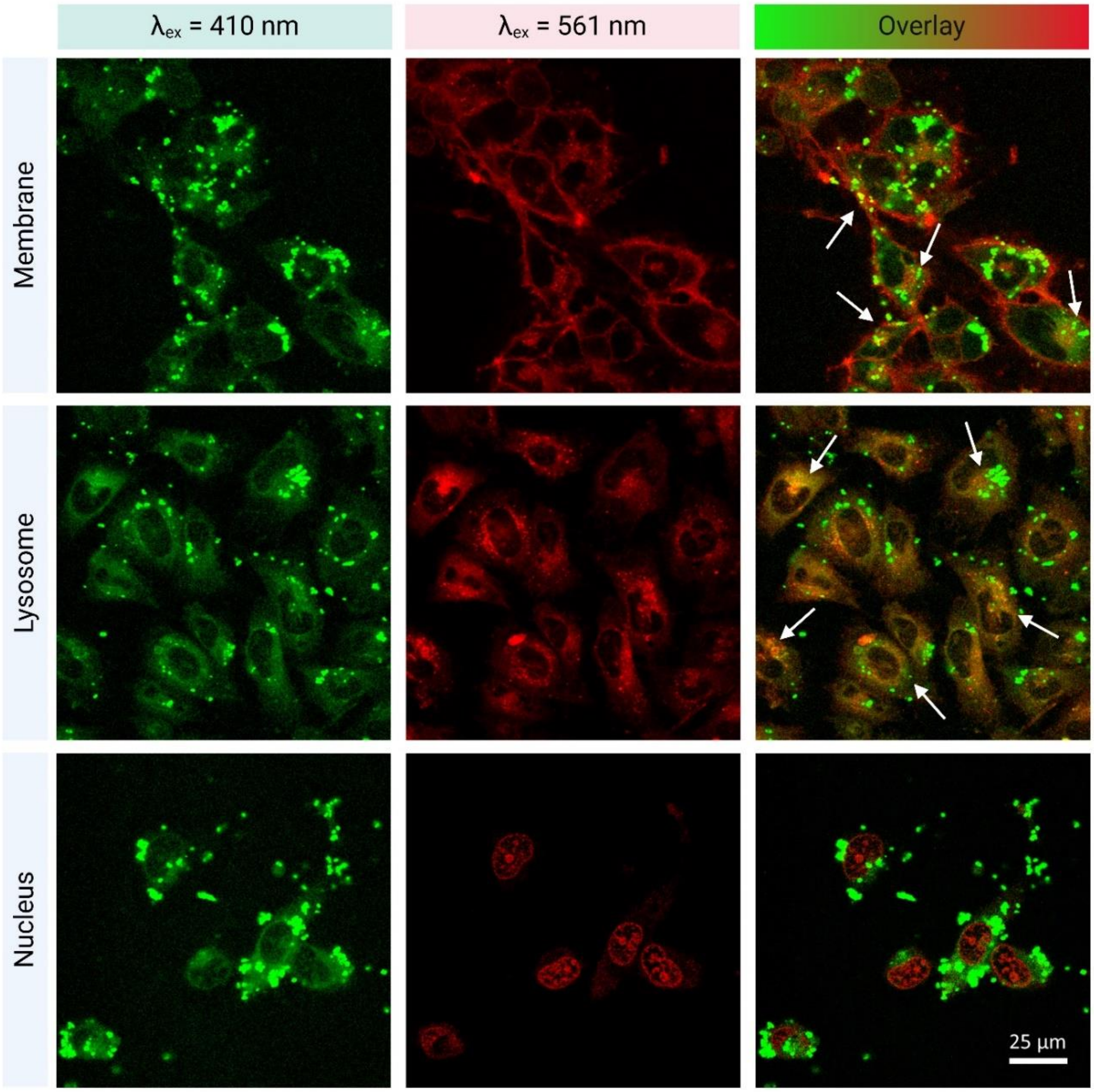

Figure S22. Confocal Laser Scanning Microscopy (CLSM) Images of U251-MG Cells Following a 4-hour Treatment with nTG-DFP-COF. Cells were treated with nTG-DFP-COF at a concentration of 10 µg/mL and co-stained using red fluorescent markers to label the plasma membrane, lysosomes, and nucleus. Imaging channels include nTG-DFP-COF (λ_{ex} = 410 nm) and red markers (λ_{ex} = 561 nm). This figure illustrates the subcellular localization and the possible intracellular pathways of nTG-DFP-COF. White arrows show co-localization.

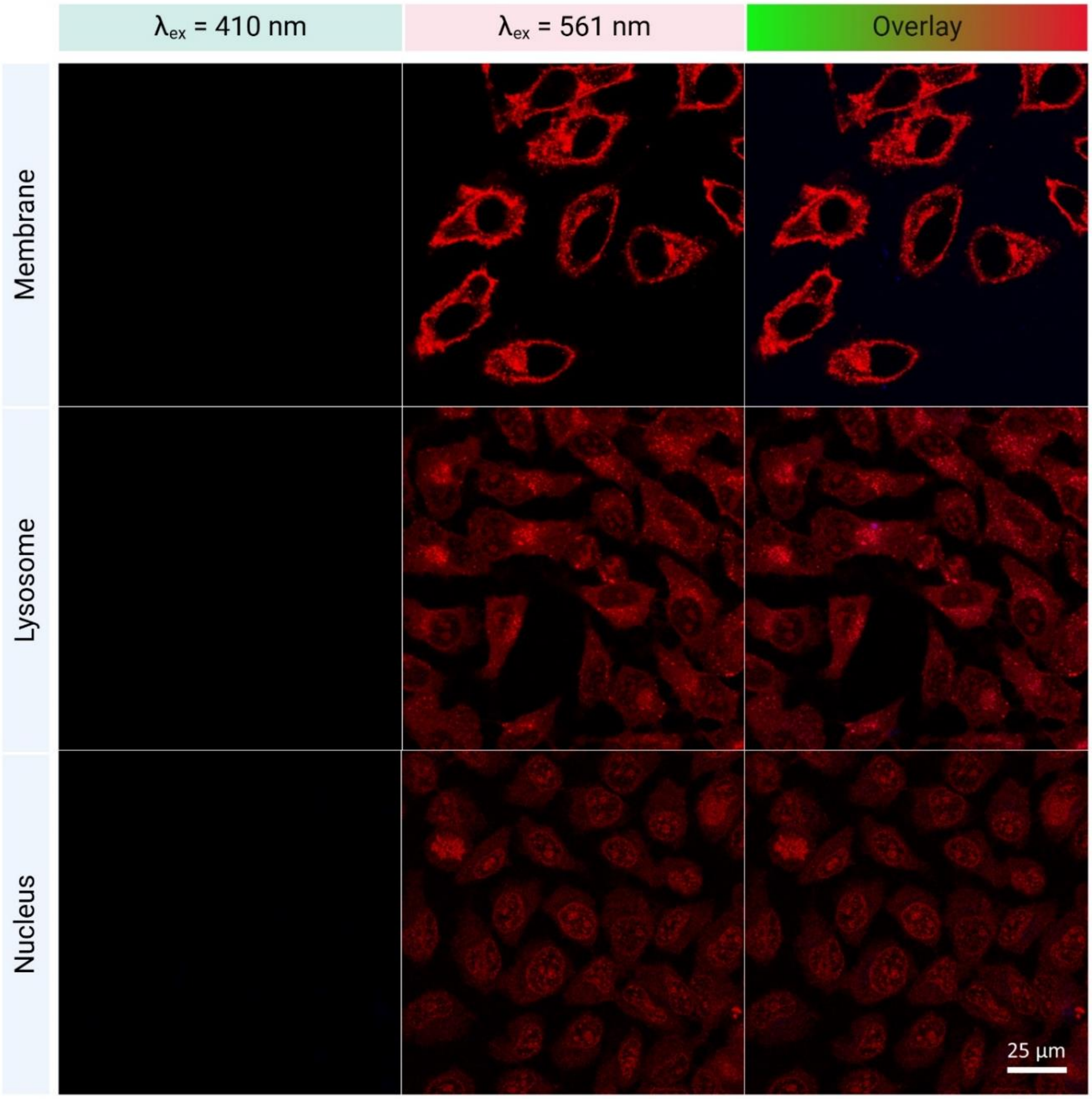

Figure S23. Confocal Laser Scanning Microscopy (CLSM) Images of HeLa Cells. These images display HeLa cells after 4 hours of incubation without additives (control) and co-stained with red fluorescent markers to visualize the plasma membrane, lysosomes, and nucleus. Imaging channels include an excitation wavelength of 410 nm and 561 nm for the red markers.

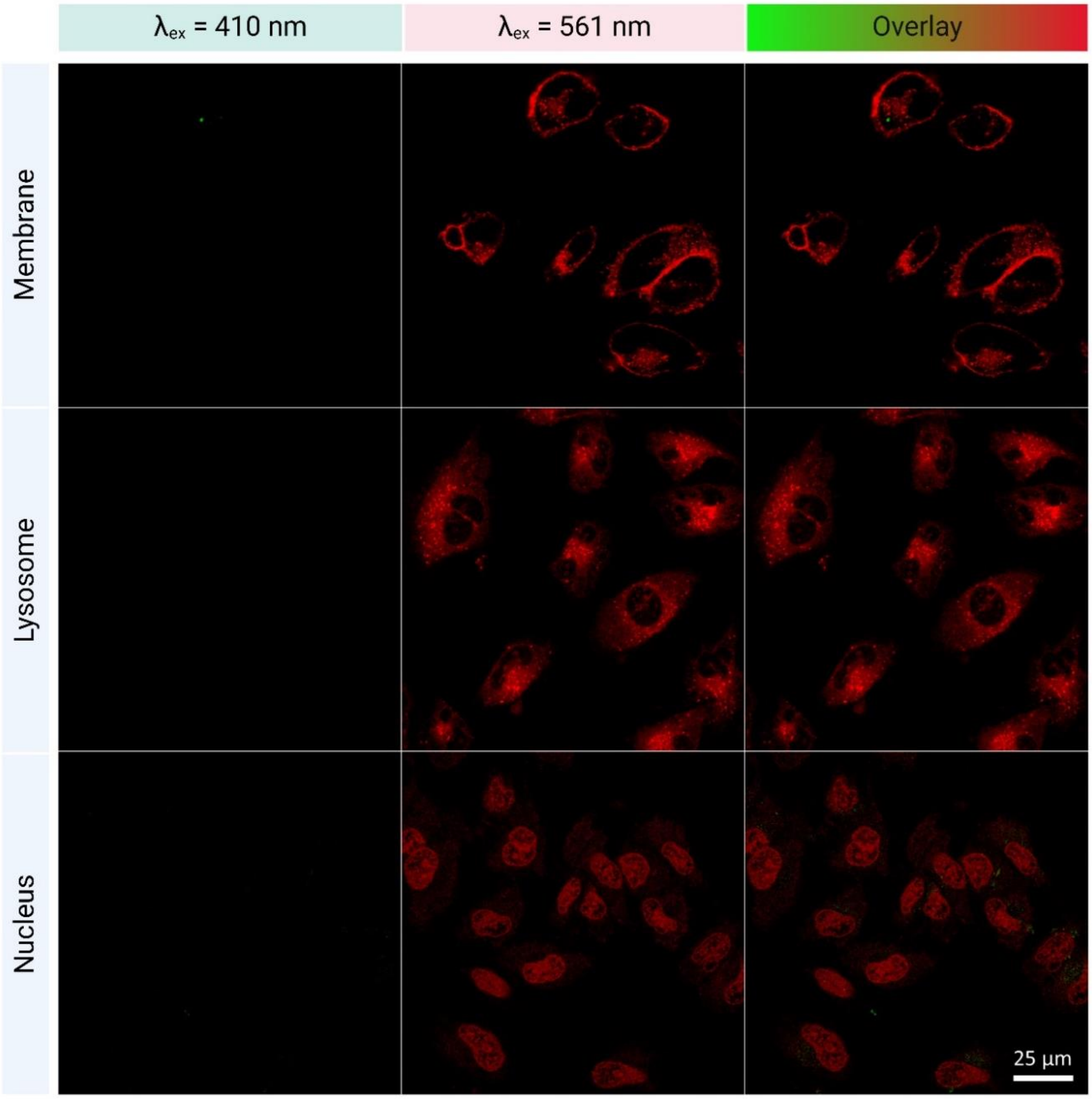

Figure S24. Confocal Laser Scanning Microscopy (CLSM) Images of U251-MG Cells. These images display U251-MG cells after 4 hours of incubation without additives (control) and co-stained with red fluorescent markers to visualize the plasma membrane, lysosomes, and nucleus. Imaging channels include an excitation wavelength of 410 nm and 561 nm for the red markers.

4.7. Bioimaging Experiments

HeLa, U251-MG, and HEK-293 cells were seeded in 6-well plates at a density of 2×10^5 cells per well in complete DMEM and incubated for 24 hours to allow attachment and growth. Following the initial incubation, cells were subjected to two different treatments. For the control group, cells were incubated for an additional 24 hours without any additives. For the treatment group, cells were incubated with nTG-DFP-COF (10 µg/mL) and simultaneously stained with LysoTracker

Red (250 nM) for 15 minutes during the last part of the 24-hour period. After treatment, cells in all groups were washed with PBS and fixed with a 3.7% paraformaldehyde solution for 10 minutes, then washed three times with PBS.

Both treated and control samples were analyzed using a Lionheart FX automated microscope at room temperature and in a frozen state (−10°C, using cold ethanol, with the temperature verified by a thermal camera). We acquired green fluorescence images utilizing a GFP filter cube (Agilent, Part Number: 1225101, excitation wavelength 469/35 nm, emission wavelength 525/39 nm). For imaging with LysoTracker Red, we employed an RPF filter cube (Agilent, Part Number: 1225103, excitation wavelength 531/40 nm, emission wavelength 593/40 nm). Each sample was assayed in duplicate, and the entire experiment was repeated three times to ensure the reproducibility and reliability of the results.

5. *In vivo* Biological Studies

All animal experiments were conducted in compliance with the policies of the New York University Institutional Animal Care and Use Committee (IACUC). Athymic NU/J nude mice and CD-1 mice (4–6 weeks old, approximately 20 g) were housed under standard conditions with 12-hour light/dark cycles and provided ad libitum access to food and water. The study protocols were approved by the Institutional Animal Care and Use Committee of NYUAD, and protocols were conducted in accordance with the guidelines outlined in the National Institute of Health Guide for Care and Use of Laboratory Animals (IACUC protocol numbers: 23-0001 and 24-0005).

5.1. *In Vivo* Toxicity and Biocompatibility Assessment of nTG-DFP-COF in CD-1 Mice

CD-1 mice were randomly divided into two groups (n=3) and treated with either 0.1 mL saline (control) or nTG-DFP-COF (20 mg/kg in 200 µL) by intraperitoneal injection. Body weight was recorded every 2 days for 7 days. No behavioral changes were observed in the treated mice compared to the control group, indicating that no acute toxicity occurred at the administered dose. The survival rate was 100% throughout the study, with no visible signs of irritation at the injection site, such as swelling, redness, pain, or heat. Behavioral observation also confirmed that the treated animals experienced no pain or discomfort.

Seven days after treatment, the mice were sacrificed, and the major organs, such as the liver, spleen, kidneys, and heart, were removed. Each organ was rinsed with PBS. Ex vivo bioluminescence imaging was performed using the IVIS® Spectrum imaging system to investigate possible organ-specific accumulation and toxicity. Images were analyzed with Living Image software, which used a spectral unmixing approach to distinguish the specific fluorescence signal of nTG-DFP-COF from the inherent fluorescence background. The autofluorescence background was quantified for accurate comparison with untreated control mice.

Histopathology: The main organs (liver, spleen, kidneys, and heart) were harvested and processed for histologic examination. The tissue sections were stained with hematoxylin and eosin (H&E) and viewed under an optical microscope (Leica DMI 6000) to assess any histopathological changes.

5.2. Tumor Model Establishment

The U251-MG cancer model was employed as an example of cancer. 5×10^6 U251-MG cells suspended in 200 µL of DMEM medium were injected subcutaneously into the right axillary region of nude mice. Tumor volumes were consistently monitored using calipers every two days post-injection. The volume was calculated using the formula $V=0.5\times length\times(width)^2$. Once the tumors reached a size of approximately 75-100 mm^3—typically about 10 days post-inoculation.

5.3. *Ex vivo* Cryo-imaging

To evaluate the potential of nTG-DFP-COF for image-guided cryosurgery, we conducted an *ex vivo* study utilizing an orthotopic U251-MG glioblastoma model. Upon the tumors reaching a volume of approximately 75-100 mm³, the tumor-bearing mice were humanely euthanized using CO_2, and the tumors were excised for further analysis.

Each excised tumor was then treated with one of two treatments: nTG-DFP-COF at a concentration of 20 mg/kg in 200 µL (n=4) or phosphate-buffered saline (PBS) as a control (n=4). Initial imaging of the tumors was performed at a physiological temperature of 37 °C using the IVIS® Spectrum system (Revvity, USA).

Following the initial imaging, the tumors from each treatment group were rapidly frozen in liquid nitrogen for 10 minutes and imaged again. This step was intended to simulate the effects of cryosurgery.

Ex vivo fluorescence imaging of the excised tumors was carried out both at 37 °C and after the freezing process. Imaging settings on the IVIS® Spectrum were configured as follows: a 465 nm excitation filter, 540 nm emission filter, binning factor of 8, f/stop of 2, field of view set to 'C', and automatic exposure time determined by the system. For quantitative analysis, regions of interest (ROIs) of uniform dimensions were drawn around each tumor, and the total radiance efficiency (normalized data) was calculated.

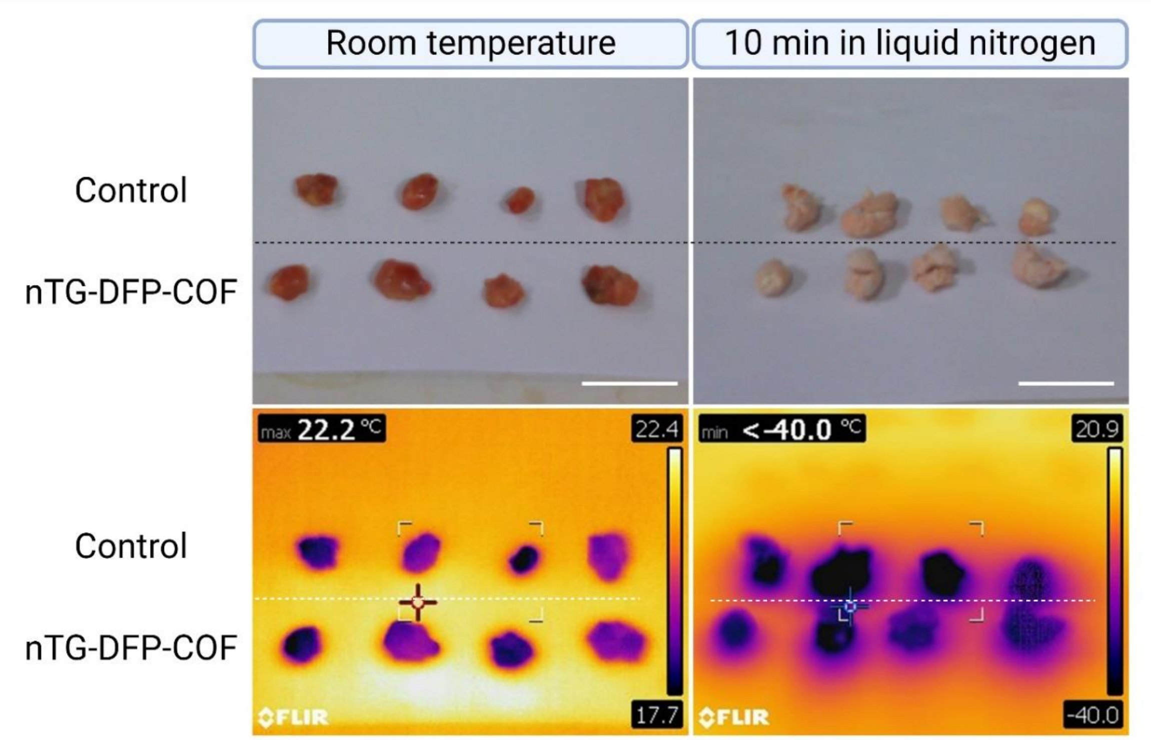

Figure S25. Visual and Thermal Analysis of U251-MG Tumors Treated with nTG-DFP-COF. This figure presents both photographs and thermal images of control U251-MG tumors (untreated) and those treated with nTG-DFP-COF (20 mg/kg, 200 µL) at room temperature and in a frozen state. The tumors were frozen by immersing them in liquid nitrogen for 10 minutes. The thermal images show the temperature distribution of the control tumors and the treated tumors immediately after removal from the liquid nitrogen, highlighting the differences in thermal response. Scale bar: 1 cm.

5.4. Local Cryotherapy in Tumor-Bearing Mice

To investigate the feasibility of local cryotherapy in tumor-bearing mice, we employed a nitrogen freeze spray in post-mortem experiments to cool tumors to −10 °C and carefully monitored them with a thermal imaging camera to ensure precision and safety (Figure S26). This method allowed the targeted application of cold temperatures specifically to tumor sites without affecting the adjacent tissue.

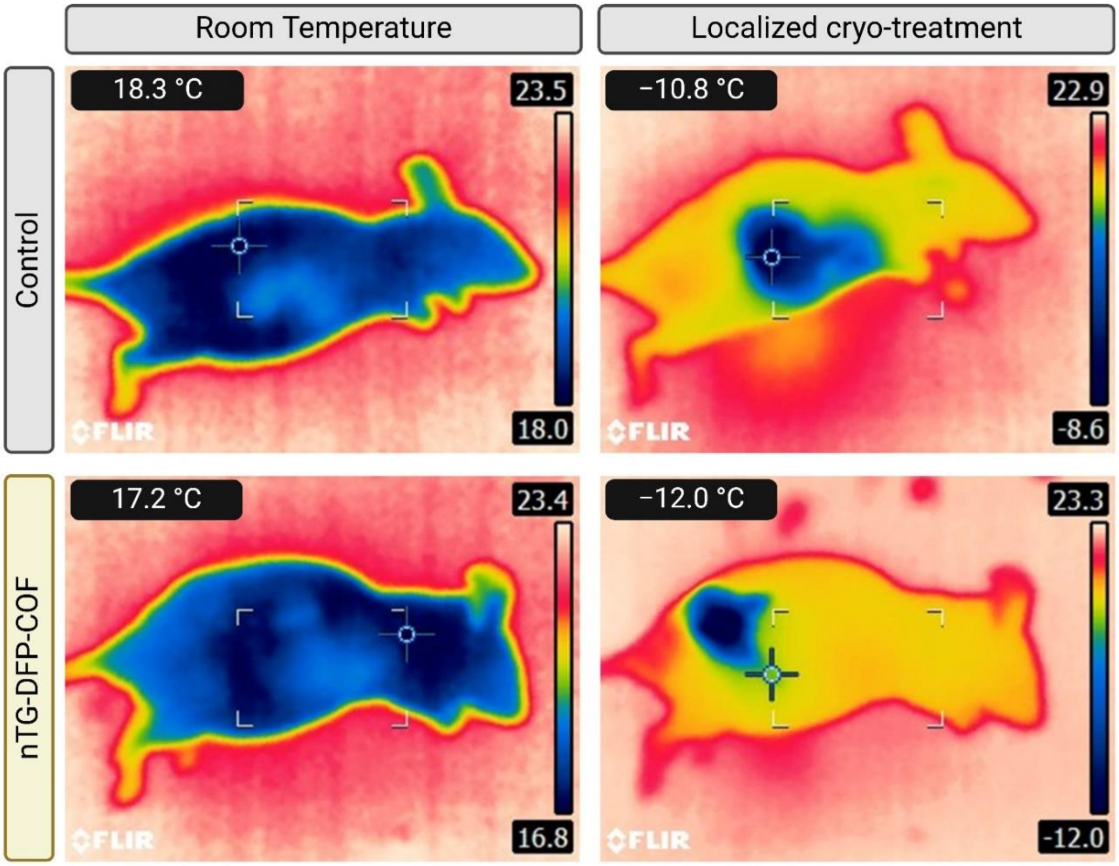

Figure S26. Thermal Imaging Analysis of Mice Subjected to Local Cryo-Treatment with nTG-DFP-COF. This figure shows thermal images of a comparison between a control mouse and a mouse treated with nTG-DFP-COF, both at room temperature and during localized cryo-treatment. The

left panel shows the baseline temperature distribution in the body of each mouse at room temperature. The right panel shows the dramatic temperature change in the tumor regions after local cryo-treatment, which selectively cools the tumors to −10.8 °C in the control mouse and −12.0 °C in the nTG-DFP-COF-treated mouse. These images visually demonstrate the precision of targeted cooling.

5.5. Post-mortem Biodistribution and Cryo-Imaging Study on Tumor-Bearing Mice

To evaluate the tumor-targeting properties of nTG-DFP-COF, tumor-bearing mice were randomly divided into two groups and injected intraperitoneally with 0.2 mL saline (control) or nTG-DFP-COF (20 mg/kg, 200 µL). 0, 24, and 48 hours after injection of nTG-DFP-COF, the animals were humanely sacrificed and subjected to post-mortem fluorescence imaging using the IVIS Spectrum.

To evaluate the tumor cryo-imaging properties of nTG-DFP-COF, tumor-bearing mice were randomly divided into two groups and injected intraperitoneally with 0.2 mL saline (control) or nTG-DFP-COF (20 mg/kg, 200 µL). 24 hours post-injection of nTG-DFP-COF, animals were humanely sacrificed and subjected to post-mortem fluorescence imaging using the IVIS Spectrum at 37 °C, 4 °C, and −20 °C.

Imaging parameters included 430 nm and 465 nm excitation, 500-540 nm emission, a binning factor of 8, f-stop of 2, a field of view set to 'C' and automatic exposure time. Spectral unmixing was performed with Living Image software to isolate the fluorescence signal of the nTG-DFP-COF from tissue autofluorescence. Tumors from untreated control mice were used to establish background autofluorescence. Images were analyzed by drawing regions of interest (ROIs) around the tumors in the unmixed images, and total radiance efficiency (normalized data) was used for quantification.

5.6. Statistical Analysis.

All statistical analysis was performed with GraphPad PRISM 8. All data are expressed as mean ±SD. Data were analyzed using one-way ANOVA with post hoc Tukey tests SPSS (IBM, SPSS Statistics, version 23, USA). ** p <0.05; ** p <0.01; *** p <0.001.*

References

1. He, Z.; Liu, P.; Zhang, S.; Yan, J.; Wang, M.; Cai, Z.; Wang, J.; Dong, Y., A Freezing-Induced Turn-On Imaging Modality for Real-Time Monitoring of Cancer Cells in Cryosurgery. *Angewandte Chemie International Edition* **2019,** *58* (12), 3834-3837.
2. Ou, W.; Stewart, S.; White, A.; Kwizera, E. A.; Xu, J.; Fang, Y.; Shamul, J. G.; Xie, C.; Nurudeen, S.; Tirada, N. P.; Lu, X.; Tkaczuk, K. H. R.; He, X., In-situ cryo-immune engineering of tumor microenvironment with cold-responsive nanotechnology for cancer immunotherapy. *Nature Communications* **2023,** *14* (1), 392.
3. Wang, Z.; He, X.; Yong, T.; Miao, Y.; Zhang, C.; Zhong Tang, B., Multicolor Tunable Polymeric Nanoparticle from the Tetraphenylethylene Cage for Temperature Sensing in Living Cells. *Journal of the American Chemical Society* **2020,** *142* (1), 512-519.
4. Gao, H.; Kam, C.; Chou, T. Y.; Wu, M.-Y.; Zhao, X.; Chen, S., A simple yet effective AIE-based fluorescent nano-thermometer for temperature mapping in living cells using fluorescence lifetime imaging microscopy. *Nanoscale Horizons* **2020,** *5* (3), 488-494.
5. Wang, C.; He, Y.; Xu, Y.; Sui, L.; Jiang, T.; Ran, G.; Song, Q., "Light on" fluorescence carbon dots with intramolecular hydrogen bond-regulated co-planarization for cell imaging and temperature sensing. *Journal of Materials Chemistry A* **2022,** *10* (4), 2085-2095.
6. Das, G.; Benyettou, F.; Sharama, S. K.; Prakasam, T.; Gándara, F.; de la Peña-O'Shea, V. A.; Saleh, N. i.; Pasricha, R.; Jagannathan, R.; Olson, M. A.; Trabolsi, A., Covalent organic nanosheets for bioimaging. *Chemical Science* **2018,** *9* (44), 8382-8387.
7. Mitra, S.; Kandambeth, S.; Biswal, B. P.; Khayum M, A.; Choudhury, C. K.; Mehta, M.; Kaur, G.; Banerjee, S.; Prabhune, A.; Verma, S.; Roy, S.; Kharul, U. K.; Banerjee, R., Self-Exfoliated Guanidinium-Based Ionic Covalent Organic Nanosheets (iCONs). *Journal of the American Chemical Society* **2016,** *138* (8), 2823-2828.
8. Das, G.; Ibrahim, F. A.; Khalil, Z. A.; Bazin, P.; Chandra, F.; AbdulHalim, R. G.; Prakasam, T.; Das, A. K.; Sharma, S. K.; Varghese, S.; Kirmizialtin, S.; Jagannathan, R.; Saleh, N. i.; Benyettou, F.; Roz, M. E.; Addicoat, M.; Olson, M. A.; Rao, D. S. S.; Prasad, S. K.; Trabolsi, A., Ionic Covalent Organic Framework as a Dual Functional Sensor for Temperature and Humidity. *Small n/a* (n/a), 2311064.
9. Dobrovolskaia, M. A.; Aggarwal, P.; Hall, J. B.; McNeil, S. E., Preclinical Studies To Understand Nanoparticle Interaction with the Immune System and Its Potential Effects on Nanoparticle Biodistribution. *Mol. Pharm.* **2008,** *5* (4), 487-495.
10. Dobrovolskaia, M. A.; McNeil, S. E., Understanding the correlation between in vitro and in vivo immunotoxicity tests for nanomedicines. *J. Control. Release* **2013,** *172* (2), 456-466.
11. Morera, D.; MacKenzie, S. A., Is there a direct role for erythrocytes in the immune response? *Vet. Res.* **2011,** *42* (1), 89-89.
12. Macías-Martínez, B. I.; Cortés-Hernández, D. A.; Zugasti-Cruz, A.; Cruz-Ortíz, B. R.; Múzquiz-Ramos, E. M., Heating ability and hemolysis test of magnetite nanoparticles obtained by a simple co-precipitation method. *J. Appl. Res. Technol.* **2016,** *14* (4), 239-244.
